# Promiscuity Guided Evolution of Decarboxylative Aldolases for Synthesis of Tertiary γ‐Hydroxy Amino Acids

**DOI:** 10.1002/anie.202422109

**Published:** 2025-02-05

**Authors:** Meghan E. Campbell, Amanda R. Ohler, Matthew J. McGill, Andrew R. Buller

**Affiliations:** ^1^ Department of Chemistry University of Wisconsin-Madison USA Madison WI 53706

**Keywords:** Protein engineering, directed evolution, noncanonical amino acids, biocatalysis, enamines

## Abstract

Many applications of enzymes benefit from activity on structurally diverse substrates. Here, we sought to engineer the decarboxylative aldolase UstD to perform a challenging C−C bond forming reaction with ketone electrophiles. The parent enzyme had only low levels of activity, portending multiple rounds of directed evolution and a possibility that mutations may inadvertently increase the specificity of the enzyme for a single model screening substrate. We show how to intentionally guide UstD towards generality through multi‐generational directed evolution using substrate‐multiplexed screening (SUMS). Mutations outside of the active site that impact catalytic function were immediately revealed by shifts in promiscuity, even when the overall activity was lower. By re‐targeting these distal residues that couple to the active site with saturation mutagenesis, broadly activating mutations were readily identified. When analyzing active site mutants, SUMS identified both specialist enzymes that would have more limited utility as well as generalist enzymes with complementary activity on diverse substrates. These new UstD enzymes catalyze convergent synthesis of non‐canonical amino acids bearing tertiary alcohol side chains. This methodology is easy to implement and enables the rapid and effective evolution of enzymes to catalyze desirable new functions.

## Graphical abstract

Promiscuity guided evolution of the decarboxylative aldolase, UstD, resulted in an efficient biocatalyst for synthesis of tertiary γ‐hydroxy non‐canonical amino acids. Simultaneous collection of variant activity and promiscuity was enabled by competition screening during directed evolution. Changes in promiscuity effectively identified distal residues that influence catalysis, a longstanding challenge in protein engineering.

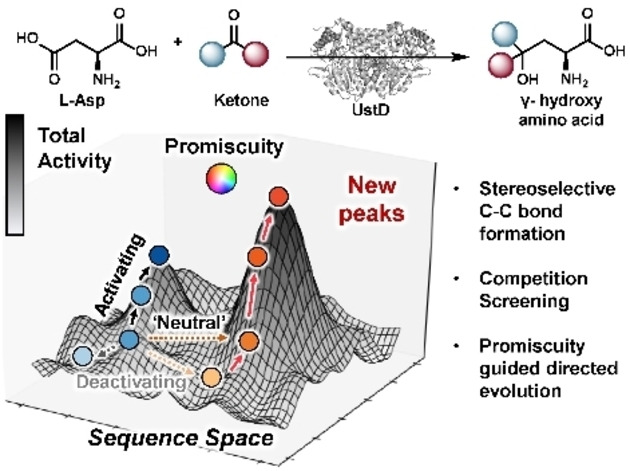

## Introduction

Enzymes are used in myriad settings, including synthesis of pharmaceuticals, fine chemicals, and bioremediation.[^1^, ^2^, ^3^, ^4^] These industrial enzymes primarily perform functional group interconversions, such as ketone reduction and transamination. ^[4,5]^ There have also been notable success in C−C bond formation, but still few enzymes that operate on preparative scales when compared to the wealth of transformations in traditional organic synthesis.[^6^, ^7^, ^8^] There has been significant progress with aldol addition into aldehydes,[^9^, ^10^, ^11^, ^12^, ^13^, ^14^, ^15^] but there are only rare examples of aldol‐addition into ketones.[^6^, ^7^, ^8^] Such reactions yield chiral tertiary alcohols, which are a highly sought motif in medicinal chemistry, due to their presence in biologically active natural products.[^16^, ^17^, ^18^, ^19^] Because ketones are more stable and kinetically slower to react than aldehydes, it is impractical or thermodynamically impossible for many classical aldolases to perform a high‐yielding transformation with ketones.[^20^, ^21^] There remains a need for robust C−C bond forming enzymes capable of intercepting ketone electrophiles for the synthesis of chiral tertiary alcohols.

**Figure 1:**
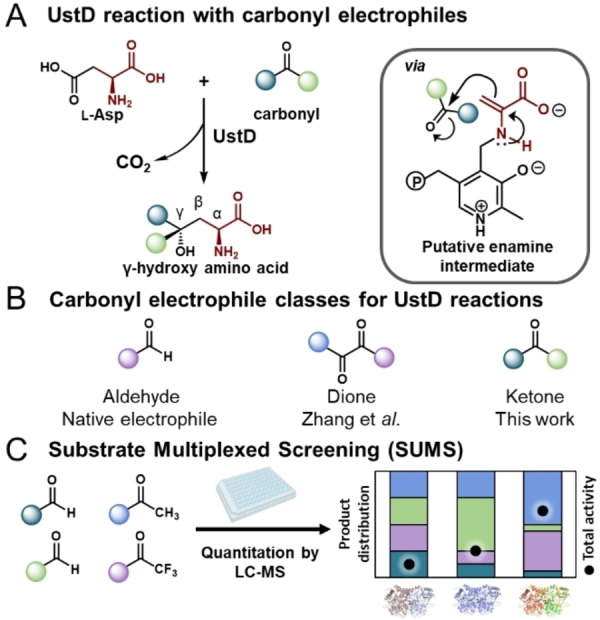
Decarboxylative aldol reaction of UstD and screening methodology with SUMS. A. UstD reaction Scheme depicting the native reactivity with aldehyde electrophiles. B. The electrophile classes compatible with UstD reactions. C. General SUMS procedure.

Directed evolution of the enzyme UstD may offer a path to access non‐standard amino acids bearing a chiral tertiary alcohol motif. UstD catalyzes the final step of ustiloxin B biosynthesis. The enzyme generates an enamine nucleophile that adds into an aldehyde in a convergent C−C bond forming reaction yielding a γ‐hydroxy non‐canonical amino acid (ncAA) (Figure 1A).[^22^, ^23^] This class of ncAAs have been reported as key intermediates in the biosynthesis of bioactive natural products.^[24]^

We reported directed evolution of UstD from *Aspergillus flavus* for improved activity using benzaldehyde as a model substrate.^[11]^ This effort, serendipitously, generated a ‘generalist’ enzyme, dubbed UstD^2.0^, capable of reacting with diverse aldehyde electrophiles to generate chiral secondary alcohols. Subsequently, Zhang et al. utilized a homolog of UstD from *Aspergillus pseudonomius* to demonstrate the broad native activity of *Ap*UstD with benzaldehyde derivatives.^[25]^ Both characterized homologs have low soluble expression in *E. coli* and come from a niche area of secondary metabolism where the native sequence diversity is limited to mesophilic organisms. A recent report by Zhang et al. demonstrated *Ap*UstD can react with di‐ketones. However, the reaction was limited to the activated dione motif and was unable to be extended to simpler ketones lacking significant electronic activation (Figure 1B).^[26]^

Recently, our group explored aldol addition into unactivated ketones using the PLP dependent threonine transaldolase, ObiH.^[27]^ This enzyme generates a resonance‐stabilized glycyl enolate nucleophile through retroaldol cleavage of the Thr sidechain, releasing acetaldehyde. The nucleophile subsequently adds into ketones to yield tertiary alcohols. On its own, this transaldolase reaction is still thermodynamically unfavorable, but coupled reduction of acetaldehyde provided a strong driving force independent of the product formed. Key to this system is the generation of a nucleophile that undergoes C−C bond formation faster than protonation. Detailed mechanistic analysis revealed that this lynchpin kinetic property is intrinsic to the PLP‐glycyl enolate itself, independent of the enzyme, and is not inherent to less conjugated enolates.^[27]^ Whether the enamine nucleophile of UstD could be engineered to react efficiently with structurally diverse ketones, which requires avoiding protonation, is wholly unknown.

We speculated that directed evolution of UstD for aldol addition to ketones would require extensive mutagenesis, since the starting enzymes have only low levels of activity with activated ketones. In standard implementations of directed evolution, a single model substrate is chosen for screening.[^28^, ^29^, ^30^] When activity on a single substrate is the desired goal, this process is uniquely powerful. When broad activity with diverse substrates is the goal, this method can also be successful, although it is typically unknown whether alternative variants that were screened might have activity with other substrates not under selection.[^31^, ^32^, ^33^] Indeed, there are many cases where directed evolution yields a catalyst that has high activity for the model substrate but struggles to react with analogs.[^34^, ^35^, ^36^, ^37^, ^38^] In some cases, intermediates along a directed evolution lineage are more promiscuous and evolution inadvertently limited the scope of the transformation.[^34^, ^35^, ^36^, ^37^, ^38^] More broadly, the inability to efficiently track substrate promiscuity during the initial screening phases hinders the ability to engineer enzymes towards generality. Since the goal of evolution with UstD would be to generate a synthetically useful catalyst with broad promiscuity, we consider alternative engineering strategies to ensure enzyme generality.

There is no colorimetric assay for UstD activity and screening requires relatively slow LC‐MS methods, a limitation that is common in synthetically motivated biocatalysis. Hence, there is an impetus to set up assays so that they are as informative as possible while bound to this relatively slow screening modality. We have been advancing substrate multiplexed screening (SUMS) as a strategy for directed evolution. By screening substrates in competition, changes to total activity and relative product abundance (promiscuity), are measured simultaneously (Figure 1C). Because LC/MS can resolve all such products in a single run, there is no added experimental burden during screening.[^39^, ^40^] Multiplexing methods have a long history of use in enzymology and chemical biology to directly measure substrate specificity.[^41^, ^42^, ^43^, ^44^] Examples of SUMS in protein engineering are sparser. SUMS was used to discover large boosts in activity with defined subclasses of substrates using small active site libraries.[^15^, ^45^, ^46^, ^47^, ^48^] In other instances, SUMS was used to monitor previously existing promiscuity and to ensure mutations that turn on undesired activity are not accumulated during evolution.[^49^, ^50^] In each of these cases, data analysis was focused on apparent increases in activity by treating each product independently or as an average of total‐activity. Here, we consider how information from the relative product abundance, wholly separated from activity changes, might be used to drive decision‐making in the engineering process. Specifically, we hypothesized that SUMS could be leveraged reliably to identify distal mutations that directly impact enzyme promiscuity, a long‐standing challenge in protein engineering.[^40^, ^51^, ^52^]

## Results and Discussion

### Identification of distal ‘hotspots’ from global random mutagenesis

**Figure 2:**
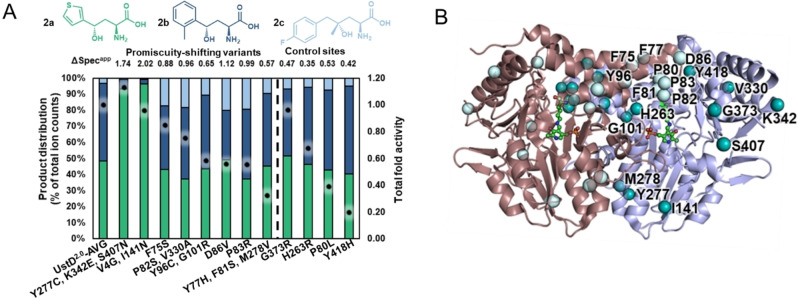
Distal putative promiscuity‐shifting sites identified by global random mutagenesis. A. Screening data for global random mutagenesis from which promiscuity‐shifting variants were identified. UstD^2.0^‐AVG is the average product distribution for the UstD^2.0^ controls from each plate. Total fold activity for all products is represented by dots. Product distribution is represented by bars. See Supporting Information Methods for screening conditions. B. UstD^2.0^ structure showing the location of the distal residues in each monomer. The teal spheres are located on chain B (purple) while the light cyan spheres are located on chain C (pink). The PLP cofactor (green) is shown in the internal aldimine form.

By screening on a substrate mixture, we hypothesized that changes in the relative rates of reactivity would aid in the identification of residues outside the active site that are influencing catalysis. We first considered the design of the substrate space by testing UstD^2.0^ activity against a mixture of substrates with distinct steric and electronic properties (Figure 2A).

Two aldehydes, thiophene‐3‐carboxaldehyde (**1** **a**), *o*‐tolualdehyde (**1** **b**) were chosen as well as a ketone lacking a strong electron withdrawing group adjacent to the carbonyl (**1** **c**). We screened 880 clones from a global random mutagenesis library (see Supporting Information for details). From this screen we identified no variants with general boosts in activity on all substrates. Some variants, however, appeared to have changes in promiscuity. Diverse strategies to quantitate promiscuity have been developed.[^48^, ^53^] Changes in product profiles can be sensitive to reaction conditions and catalyst concentration, as well as underlying shifts in specificity.^[40]^ We calculated the cumulative, apparent specificity change for each variant relative to parent, ΔSpec^app^ (described further in the Supporting Information). Interpretation of calculations that normalize to total product formed can be convoluted by the noise associated with low‐intensity measurements. Such measurements are often characteristic of the early phases of challenging directed evolution efforts (ie. product **2** **c**). We therefore applied a strict cutoff for consideration of ΔSpec^app^ to variants that had at least 20 % total activity with respect to UstD^2.0^. We used ΔSpec^app^ >0.5 as a means of quantitatively describing the shifts in promiscuity that were apparent from visual inspection. Based on these criteria, 8 variants were selected for sequencing, from which we identified 15 mutations (Figure 2A).

However, variants with changes in their product profiles that were close to this threshold were recognized as ‘marginal’ cases. Analysis of the UstD^2.0^ crystal structure showed that no promiscuity‐shifting mutations were in the active site and the alpha carbons (Cα) of the mutations were an average 18 Å away from the cofactor (Figure 2B). We hypothesized that these mutations may be ‘hotspots’ for altering activity. Although these specific mutations are deleterious under the screening conditions, some other mutation at the same site may be beneficial for catalysis, which we tested with site saturation mutagenesis (SSM)

### Mutation of distal promiscuity‐shifting sites reveals activating mutations.

We selected P82 (ΔSpec^app^=0.96) as an initial site to test this ‘hotspot’ hypothesis, as Pro is a unique residue and mutation here seemed conspicuous. We also considered site G373, whose ΔSpec^app^ of 0.47 was marginal, just below the threshold of significance, as a control site. To our surprise, SSM at both positions revealed generally activating mutations. When re‐screened outside of a competition setting on just **1** **c**, the top variants were P82Q, and G373E, which had 9.6, and 1.2‐fold higher activity compared to parent, respectively (Figure S2). When combined these mutations resulted in the new variant, QE, which had a 12‐fold improvement in activity with ketone **1** **c** (Figure S2). As the mutations are distal to the active site, it is difficult to identify the molecular mechanism through which these mutations operate. While we did not use structural information to identify these sites, the X‐ray structure of UstD^2.0^ showed P82 forms a cis‐peptide bond, and it is plausible that P82Q has widespread structural effects. Nevertheless, the increase in activity is significant because UstD^2.0^ activity with **1** **c** was nearly stoichiometric and crossed the threshold to catalytic, albeit a modest 17 turnovers with QE (Figure S3).

### Substrate space redesign can increase sensitivity to changes in reactivity

**Figure 3:**
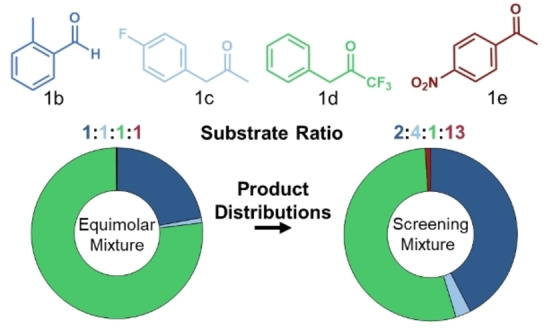
Substrate mixture design. Substrates included in the mixtures are shown above the plots. The plots show the amino acid product distribution from two different substrate mixtures with the color‐coded ratio of substrates displayed above each. On the left, substrates are added in equal amounts (12.5 mM each). On the right, substrates are added in differing amounts according to their electrophilicity (5 mM **1** **b**, 10 mM **1** **c**, 2.5 mM **1** **d**, 32.5 mM **1** **e**). **Conditions**: 50 mM l‐asp, 50 mM total electrophiles, 5 μM PLP, 5 % DMSO, 100 mM NaCl, 100 mM potassium phosphate pH 7.0, QE (0.01 mol % catalyst, 10,000 Max TON), 37 °C, 1 h reaction time.

The initial substrate mixture was chosen for its simplicity. Spurred by the success of the promiscuity‐guided evolution above, we considered whether more information might be accessible from SUMS when using a substrate mixture with more diverse electrophiles. To maintain some continuity between the global random mutagenesis and the SSM screens both **1** **b** and **1** **c** were retained in the new substrate mixture. We introduced two new ketones, 1,1,1‐trifluoro‐3‐phenyl‐2‐propanone (**1** **d**) and 4’‐nitroacetophenone (**1** **e**). In simple mixtures with equimolar concentrations, the activated electrophiles (**1** **b**, **1** **d**) dominated the overall reactivity and the signal for the tertiary alcohol product, **2** **e**, was on the order of experimental noise (Figure 3). We therefore altered the substrate concentrations to ensure reproducible and robust signals for all products via UPLC‐MS. The resulting mixture contained a 9 : 1 ratio of ketone to aldehyde substrates. In this straightforward way, substrate mixtures for multi‐generational evolutionary campaigns can be re‐tuned as reactivity is expanded.

We used QE as the parent enzyme for SSM at distal promiscuity‐shifting sites identified from globally random mutagenesis (Figure 2B). Mutagenesis at eight of 15 sites led to several variants with at least a 1.5‐fold increase in total activity during screening (Figure S5). We observed that mutations D86V and V330A both produced a 2‐fold increase in activity. We also found, serendipitously, that synonymous codon changes at I141 and S371 led to boosts in whole‐cell catalyst activity, presumably by increasing the soluble enzyme expression. We again screened a SSM library at a control site. The Y418H mutation was identified by global random mutagenesis as one that decreased activity with a ΔSpec^app^ value of 0.42. As expected, SSM at Y418 did not lead to increases in activity (Figure S6).

This two‐step process of identifying ‘hotspots’ by promiscuity shifts using ΔSpec^app^ followed by SSM led to activating mutations with no prior information about protein structure, dynamics, or evolution. This strategy is distinct from stereo‐ or regioselectivity‐based screening, which only considers positions whose initial hits provide the desired change in selectivity.[^54^, ^55^] The key addition made by our approach is that *any* shift in promiscuity, not just one in the desired direction, is now recognized as a ‘hit’ for subsequent mutagenesis. While future studies may untangle the basis of these activating effects for this particular enzyme, the present investigation is focused on the development of a practical engineering approach that incorporates promiscuity information. We continued by deploying another common step in enzyme evolution: recombination of activating mutations.

### Identification of cooperative mutational effects in a recombination library

There are many successful strategies for designing and screening recombination libraries.[^56^, ^57^, ^58^, ^59^, ^60^] We considered five different positions (F75, D86, I141, V330, S407) that were distributed across the protein structure. Screening data was used to identify degenerate codons that limit the inclusion of mutations that are deleterious when introduced independently (Figure S5, S7). The resulting library consisted of ~2,800 possible variants (See Table S1). Here we acknowledge a tradeoff between library size and screening intensity. It is not necessary to exhaustively screen all possible combinations, but rather to use a carefully crafted library to efficiently traverse the largest practically, accessible recombination space.

We maintained the substrate mixture from the previous round of evolution (Figure 3) and increased reaction time from one hour to eight hours to reduce the risk of selecting destabilizing mutations or those that accelerate decomposition of product through retro‐aldol cleavage. We screened 704 clones, representing a maximum of ~24 % of the theoretical library space and observed increases in total activity up to 2.6‐fold (Figure S8). The top variants were validated using single substrate reactions leading to the quadruple variant AIIRQ (F75A, D86I, V330R, S407Q). This variant displayed increased activity with all ketone substrates (~1.5–2.5‐fold change) and only a modest decrease in activity with the aldehyde (80 % parent activity, Figure S8). Additionally, AIIRQ had higher soluble expression (~80 mg protein/ L culture) compared to QE and the other recombination variants (~50 mg protein/ L culture). Therefore, AIIRQ was chosen as the new parent enzyme for subsequent evolution.

### Active site engineering reveals two mutants with distinct promiscuity

**Figure 4:**
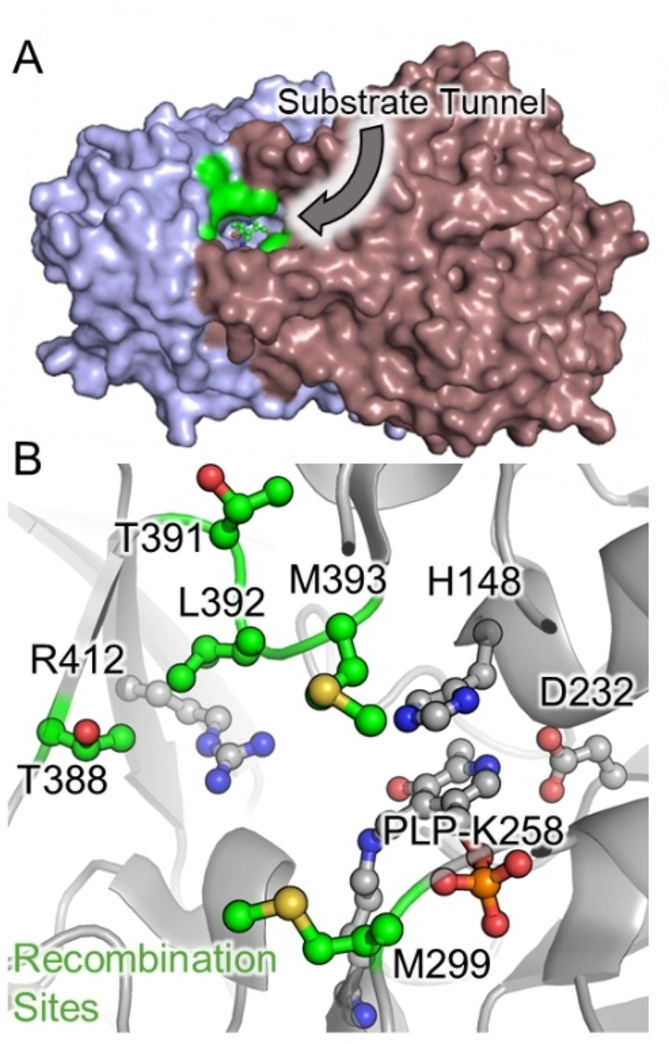
UstD active site targeted for mutagenesis. A. Space filling view of overall UstD structure. Individual monomers are colored purple (chain B) and pink (chain C) while the active site and PLP are shown in green. B. Close up view of UstD active site. The PLP complex and anchoring residues are shown in grey. The residues targeted for mutagenesis are shown in green.

Previously, we identified a loop region in the UstD active site encompassing residues 391–393 where several mutations were found that increased activity on aldehydes.^[11]^ We hypothesized that combinatorial retargeting of these sites would reveal variants with higher activity. Two additional sites, M299 and T388 were also included because both have side chains that protrude into the active site (Figure 4). We had screened a SSM library at M299 with QE as the parent, which showed conservative mutations were generally neutral to activating (Figure S9). With this information, we designed a focused recombination library of 2,300 variants. Cooperative effects are particularly common in enzyme active sites and there is a higher propensity for multiple active site mutations to be deleterious, raising the specter of laborious screening of predominately inactive sequence space.^[59]^ We therefore biased the mutational load from 3.9 to 3.3 mutations per variant during library construction (see Supporting Information for details).

We screened 968 clones within this focused library, representing a maximum 42 % of the theoretical library space. The promiscuity profiles of these variants showed more diversity than earlier screens, as expected (Figure S10). From these libraries we identified seven variants with disparate reactivity profiles (Figure S11). These activated enzymes had distinct amino acid sequences and contained an average of 3.1 mutations. None of these activated variants contained mutation at all 5 sites simultaneously, supporting the choice to decrease the mutational load. One notable variant, 7G04 (M299V, T388I) had much higher activity with **1** **c**, upwards of a 14‐fold boost, but had minimal changes with other substrates. While this activity is impressive, this variant represents a specialist that one would want to *avoid* when the goal is to develop a catalyst with general synthetic utility. We therefore selected two variants with distinct and broad reactivity profiles, 7G11 (M299V, T391S, M393W) and 7B05 (T391S, M393F) for further exploration.

### Lineage analysis of promiscuity guided evolution

Characterizing variants in an evolutionary lineage is often used to show how successive rounds of mutagenesis affect activity.[^37^, ^61^] Such retrospective analyses are typically limited to the single transformation under selective pressure, with the notable exception of evolution that features a substrate walking strategy.^[5]^ We chose to assess variant activity on substrates that were both under selective pressure (**1** **c, 1** **d**) and those that were not (**1** **g, 1** **f**), to study how evolution impacted reactivity more broadly. Prior to lineage analysis of UstD, we performed a brief survey of reaction conditions using the variant 7G11. Increasing the concentration of l‐Asp from 50 to 250 mM increased yields of **2** **c** and **2** **d** (see Supporting Information for details, Figure S12). While such high concentrations are not ideal, the amino acid is cheap and commercially available. Additional equivalents of PLP relative to enzyme (between 10–50‐fold excess) were also beneficial for both substrates. To understand how promiscuity changed through evolution, we performed a lineage analysis with a small set of substrates without competition under these optimized conditions

**Figure 5:**
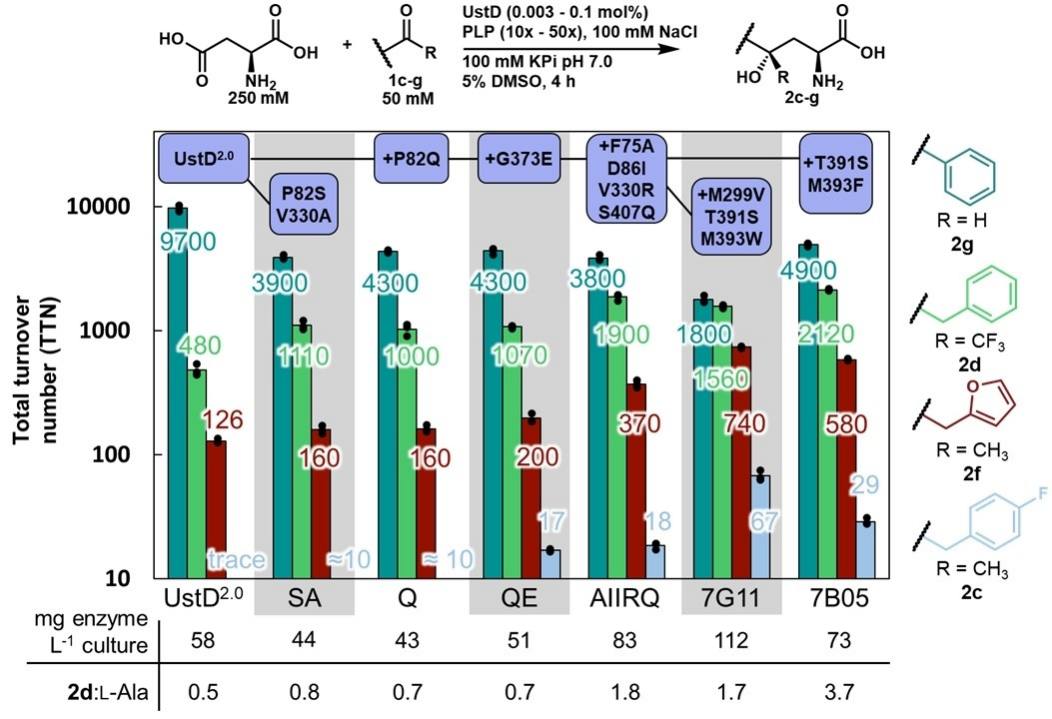
Lineage analysis of promiscuity guided evolution. Reactions were performed with single substrates in triplicate. The substrates are displayed to the right of the chart. The total turnover (TTN) number is displayed on the y‐axis on a logarithmic scale. The bar represents the average of the technical replicates, and the dots represent the TTN measurement of each individual replicate. The color of each bar corresponds to its product. The mutations associated with each variant are displayed at the top of the graph with the connecting lines representing the relationship between them. Below the chart, the yield of protein per liter of culture is displayed for each variant. The ratio of **2** **d** to the shunt pathway product, l‐Ala, is displayed below the chart to demonstrate that the ratio changes over the lineage.

We observed steady increases in activity with substrates that were under direct selective pressure (**1** **c** and **1** **d**, light blue and green), leading to the two enzymes 7B05 and 7G11 (Figure 5). The variant 7B05 performed 2,120 turnovers with **1** **d**, representing a ~4‐fold increase in activity. The activity of UstD^2.0^ with **1** **c** was negligible. The final variant, 7G11, performed a modest 67 turnovers with **1** **c**. While this reaction moved from stoichiometric to catalytic, further improvements could come from additional rounds of evolution.

We probed the effects of evolution on substrates not under selective pressure and found unexpected results. The parent enzyme, UstD^2.0^, was previously evolved for activity with benzaldehyde (**1** **g**) on which it performed ~9,700 turnovers.^[11]^ However, UstD activity with **1** **g** *decreased* steadily throughout the evolution (Figure 5, teal bars). Because aldehydes are intrinsically more reactive than ketones, we had originally anticipated that any molecular changes that accelerate the C−C bond forming step could be expected to work with both classes of substrate. In contrast, activity with furylacetone (**1** **f**) increased ~6‐fold (Figure 5, red bars), indicating that evolution enhanced activity on a substrate not under direct selective pressure.

We considered how the activity might increase with ketone substrates, but not an aldehyde. One possibility is that distal mutations increased activity by tuning the lifetime of the reactive enamine nucleophile (Figure 1B). Once the enamine forms, it experiences a kinetic competition between protonation, which quenches the nucleophile to form l‐alanine (Ala), and C−C bond formation with an electrophile.^[22]^ With highly reactive electrophiles, changes to this competition would have a negligible effect. We measured the ratio of protonation vs C−C bond formation with **1** **d** and found that protonation was favored by a factor of 2 in the parent enzyme, consistent with the challenging nature of the C−C bond formation. This rate of C−C bond formation was enhanced with the AIIRQ variant, such that C−C bond formation was preferred (Figure 5, Figure S42). This ratio further shifted with the active site recombinant 7B05, which had lower Ala formation and favored C−C bond formation by ~4‐fold.

Sequence‐activity relationships indicate that complex molecular effects are influencing activity. The variants 7B05 and 7G11 share the T391S mutation and differ in residues 299 and 393 (Figure 4). Evidence for cooperativity within the active site comes from the recombination library, where we serendipitously acquired sequence‐function data that corresponds to a stepwise mutational walk between the two activated enzymes. The two possible intermediate mutants have <15 % of parent activity (Figure S13). Hence, active site recombination captured epistatic interactions between these sites. Further assessment of enzyme mechanism and the impacts of particular mutations or collections thereof may be fertile ground for future inquiry.

### Convergent biocatalytic synthesis of chiral tertiary alcohols

**Figure 6:**
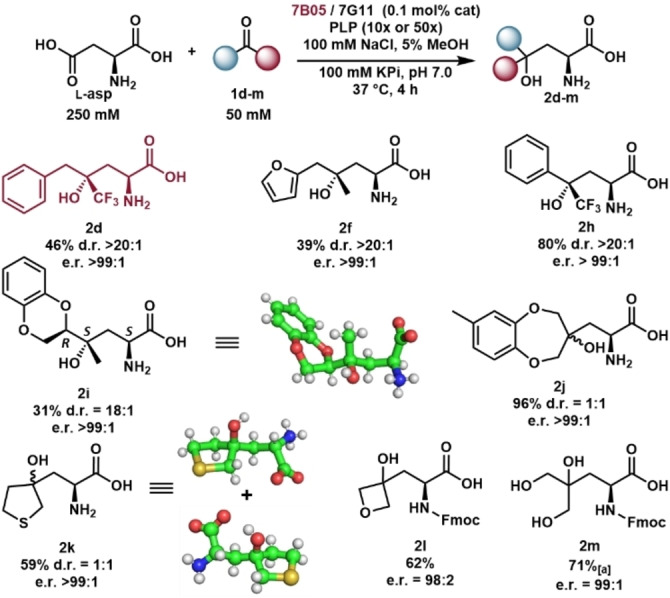
Preparative scale biocatalytic reactions. Crystal structures of **2** **i** and **2** **k** are displayed using ball and stick models. Only a single diastereomer of **2** **i** was observed. Both diastereomers were present in equal amounts for **2** **k**. [a]Catalyst loading is 0.4 mol %.

The synthetic utility of 7G11 and 7B05 was assessed on both analytical scale and preparative scale. Beginning with analytical scale analysis, we observed 7B05 had higher activity for some highly activated trifluoromethyl ketone substrates (Figure S14). In most others, 7G11 was the more proficient catalyst (Figure S14). A subset of these amino acids was chosen for preparative‐scale reactions in order to demonstrate isolation strategies for chemically diverse products. Efficient isolation of aromatic amino acids was possible using reverse‐phase flash chromatography (Figure 6, see Supporting Information for further details) and stereoselectivity was assessed using Marfey's analysis.[^62^, ^63^]

Trifluoroacetophenone (**1** **h**) reacted efficiently with 7G11 and **2** **h** was isolated in 80 % yield and excellent diastereoselectivity (>20 : 1). 7B05 converted **1** **d**, one of the substrates under selection during evolution, to **2** **d** in 46 % yield with excellent d.r. (>20 : 1). Excitingly, the heterocyclic amino acid, **2** **f** was isolated in 39 % yield and excellent d.r. (>20 : 1). Product **2** **i** was isolated in 31 % yield and high d.r. (18 : 1). A crystal structure of **2** **i** revealed the absolute configuration as 2*S*,4*S*,5*R*. Hence, 7G11 maintains the same configurational preference shown with aldehyde substrates wherein the α‐amine and γ‐hydroxy are *anti* to one another in a standard linear depiction (Figure 5, S15, S16). When the ketone is positioned within a ring, we observed exceptional activity and **2** **j** was isolated in 96 % yield as a 1 : 1 mixture of diastereomers.

The aliphatic ncAA, **2** **k** was sufficiently hydrophobic to be isolated using reverse phase chromatography in good yield (59 %) as a 1 : 1 mixture of diastereomers. Both diastereomers were structurally characterized by small molecule crystallography (Figure S17). The more hydrophilic amino acids **2** **l** and **2** **m** were isolated by addition of Fmoc in a telescoped fashion. The protected amino acids were subsequently isolated using normal‐phase chromatography. This process led to **2** **l** in 62 % yield and excellent e.r. (98 : 2). Yield of the trihydroxyleucine **2** **m**, derived from reaction with 1,3‐dihydroxyacetone (**1** **m**), was initially low (36 %). Higher yield was straightforward to obtain by increasing the catalyst loading to 0.4 mol % 7G11, which gave **2** **m** in a 71 % isolated yield (99 : 1 e.r.) as the Fmoc‐protected amino acid. Trihydroxyleucine is found in a fungal natural product^[64]^ and was previously synthesized in four steps and isolated in 32 % yield as the lactone.^[65]^ For all other amino acids, UstD enabled the first reported synthesis.

## Conclusion

We report a multigenerational protein engineering campaign that uses changes in promiscuity and activity information together to guide evolution. Distal residues that were coupled to the active site were discovered in high‐throughput via SUMS, without the need for detailed structural or mechanistic studies. This strategy represents a generalizable strategy for experimental identification of distal sites that influence catalysis, a long‐standing challenge in protein engineering.[^51^, ^52^] While recent advances in computational design and machine learning are making rapid strides in the identification of stabilizing mutations, strategies to identify mutations outside the active site that directly alter catalytic activity are more limited.[^11^, ^66^, ^67^, ^68^, ^69^, ^70^, ^71^] There are many cases where distal sites are known to increase specificity, even if activity is compromised.[^54^, ^55^] Previous methods to identify putative ‘hotspots’ rely on detailed sequence analysis,^[72]^ structural data,^[73]^ or kinetics assays using fluorescence reporters with specialized microfluidic devices.[^74^, ^75^, ^76^] The SUMS approach is a simple experimental extension of plate‐based LC–MS screening and capable of pinpointing these distal residues at a rate of 1 : 100 with nothing more than global random mutagenesis, making it immediately translatable to many engineering workflows.

Promiscuity‐guided evolution also enabled discrimination between specialist and generalist active site variants. We characterized two variants with complementary increases in activity that we used to synthesize a small set of chemically diverse amino acids. These new decarboxylative aldolases represent a significant advance as they provide a new strategy for the convergent synthesis of ncAAs containing gamma‐ tertiary alcohols. Future work will encompass mechanistic investigations into the specific molecular determinants for UstD activity and the application of promiscuity‐guided evolution to new classes of enzymes.

While SUMS engineering provides benefits traditional engineering cannot, it also has drawbacks. There is modestly more reaction optimization prior to screening and the data analysis is more complex. Promiscuity profiles allow researchers to directly observe reaction scope, a boon when pursuing generalists. However, it becomes more difficult to define the “best” variant, as activated variants can encompass changes in promiscuity, activity, or both. We emphasize, however, that such tradeoffs have always been occurring in directed evolution, researchers were simply blind to these effects during screening.

## Supporting Information

The authors have cited additional references within the Supporting Information.[^77^, ^78^, ^79^]

## Conflict of Interests

The authors are inventors on a patent related to the synthetic applications of UstD.

1

## Supporting information

As a service to our authors and readers, this journal provides supporting information supplied by the authors. Such materials are peer reviewed and may be re‐organized for online delivery, but are not copy‐edited or typeset. Technical support issues arising from supporting information (other than missing files) should be addressed to the authors.

Supporting Information

## Data Availability

The data that support the findings of this study are available from the corresponding author upon reasonable request.
