# Supplementary material for: Promiscuity Guided Evolution of Decarboxylative Aldolases for Synthesis of Tertiary γ‐Hydroxy Amino Acids
Source: Angew Chem Int Ed Engl. 2025 Feb 5;64(15):e202422109. doi: 10.1002/anie.202422109 (PMC11976203; doi:10.1002/anie.202422109)
Supplement: Supplementary file 1 — Supporting Information [file ANIE-64-e202422109-s001.pdf]

Angewandte Chemie
Eine Zeitschrift der Gesellschaft Deutscher Chemiker

Supporting Information

Promiscuity Guided Evolution of Decarboxylative Aldolases for Synthesis of Tertiary γ-Hydroxy Amino Acids

*M. E. Campbell, A. R. Ohler, M. J. McGill, A. R. Buller**

Supporting Information for

Promiscuity Guided Evolution of Decarboxylative Aldolases for Synthesis of Tertiary γ-Hydroxy Amino Acids.

Meghan E. Campbell, Amanda R. Ohler, Matthew J. McGill, Andrew R. Buller*

*Andrew R. Buller
Email: arbuller@wisc.edu

This PDF file includes:

Supporting text
Figures S1 to S40
Tables S1 to S7
SI References

Supporting Information Text

Materials and methods

General materials and methods. All chemicals and reagents were purchased from commercial suppliers (Sigma-Aldrich, VWR, Chem-Impex International, Alfa Aesar, Combi-blocks, Oakwood Products) at the highest quality available and used without further purification unless stated otherwise. Genes were purchased as gBlocks from Integrated DNA Technologies (IDT). *E. coli* cells were electroporated with an BioRad MicroPulser E-porator at 2500 V. New Brunswick I26R shaker incubators (Eppendorf) were used for cell growth. Cell disruption via sonication was performed with a Sonic Dismembrator (Fisherbrand) sonicator. Optical density measurements were collected using an optical density reader (Amersham Biosciences). Ultra-high pressure liquid chromatography-mass spectrometry (UPLC-MS) data were collected on an Acquity UPLC (Waters) equipped with an Acquity PDA and QDA MS detector using either a BEH C18 column (Waters) for aromatic substrates, or an Intrada Amino Acid column (Imtakt) for aliphatic substrates. Preparative column separations were performed on an Isolera One Flash Purification system (Biotage). NMR data were collected on Bruker 400 or 500 MHz spectrometers equipped with BBFO and DCH cryoprobes, respectively. All NMR chemical shifts were referenced either to a residual solvent peak or TMS internal standard. Spectra recorded using CD_3OD were referenced to the CH_3OH residual solvent peak at 3.31 ppm for ^1H and 49.00 ppm for ^{13}C NMR. Spectra recorded using D_2O:MeOD-d^4 as the solvent were referenced to the residual H_2O signal at 4.79 ppm for ^1H and absolute referenced to the ^1H spectrum for ^{13}C NMR. Spectra recorded using D_2O as the solvent were referenced to the residual H_2O signal at 4.79 ppm for ^1H and absolute referenced to the ^1H spectrum for ^{13}C NMR. Spectra recorded using DMSO-d^6 were referenced to the DMSO residual solvent peak at 2.5 ppm for ^1H and 39.52 ppm for ^{13}C NMR. Signal positions were recorded in ppm with the abbreviations s, d, t, q, dd, and m, denoting singlet, doublet, triplet, quartet, doublet of doublets, and multiplet respectively. All coupling constants *J* are measured in Hz. High resolution mass spectrometry data were collected with a Q Extractive Plus Orbitrap (NIH 1S10OD020022-1) instrument with samples ionized by ESI.

Plasmid Preparations. A 5-mL overnight culture of *E. coli* harboring the plasmid of interest was grown overnight at 37 ^oC with shaking at 200 rpm. The plasmid was isolated and purified using Zymo Plasmid Miniprep or Macherey-Nagel Nucleospin Plasmid Mini kits and sequenced through Functional Biosciences.

DNA sequences. Sequences with synonymous and missense mutations relative to UstD$^{2.0}$ have the target mutation bolded. All enzymes were expressed with a C-terminal 6xHis tag shown in blue.

DNA sequence of ustD$^{2.0}$
ATGAAGAGCGTAGCGACGAGTTCCCTTGATGACGTAGATAAAGATTCCGTCCCCCTGGGCA
GTTCGATCAATGGCACTGCACAAGCGGAAACTCCGCTGGAGAATGTGATCGACGTCGAATC
AGTGCGCTCACATTTCCCGGTATTAGGGGGGGAAACGGCCGCGTTTAACAATGCATCAGGA
ACCGTAGTTTTGAAGGAGGCAATTGAATCGACTTCAAATTTCATGTATAGCTTTCCTTTTCCC
CCGGGTGTTGACGCTAAGTCAATGGAGGCTATTACCGCATATACGGGGAATAAGGGCAAGG
TTGCGGCATTTATCAATGCACTTCCTGATGAAATTACATTCGGGCAGTCCACAACTGCCCTG
TTCCGTTTATTAGGTCTGTCGCTTAAACCTATGCTGAATAACGATTGCGAAATCGTATGCTCA
ACATTATGTCACGAAGCAGCAGCTTCCGCATGGATTCATTTAAGTCGCGAATTAGGAATTAC
CATTAAGTGGTGGAGCCCAACTACTACACCGAATAGTCCCGATGATCCAGTTCTGACGACTG
ACTCATTGAAGCCCTTGCTTAGTCCAAAAACGCGCCTTGTTACATGTAATCACGTGTCGAAT
GTTGTAGGAACCATCCACCCTATTCGTGAGATTGCCGACGTGGTACATACCATTCCTGGAGC
GATGCTTATCGTTGACGGTGTGGCAAGCGTCCCGCATCGTCCAGTTGATGTTAAAGAATTGG
ATGTAGATTTTTACTGCTTTTCCTGGTACAAGTTGTTCGGACCGCATCTTGGAACCCTGTATG
CTTCCCGCAAAGCCCAAGACCGCTATATGACCTCAATTAACCATTACTTCGTCTCATCGTCG
AGCCTTGATGGTAAGCTGGCATTAGGCATGCCGTCCTTTGAACTGCAGTTGATGTGCTCTCC

AATTGTTTCGTATTTGCAAGATACGGTGGGCTGGGACCGTATCGTGCGCCAAGAGACTGTG
CTGGTAACTATTTTGTTGGAGTATTTACTTAGCAAGCCATCTGTATATCGTGTGTTCGGACGT
CGTAATTCTGATCCCAGTCAGCGTGTAGCAATCGTAACTTTTGAAGTCGTGGGACGTAGTTC
CGGGGATGTGGCAATGCGCGTAAATACGCGTAATCGCTTCCGCATTACCTCTGGAACCTTAA
TGGCACCGCGCCCGACATGGGACGTCTTGAAACCGAAGAGTAGCGACGGACTTGTTCGCG
TCAGCTTTGTACATTACAACACGGTTGAGGAAGTGCGTGCGTTCTGCAGCGAGTTAGACGA
GATTGTGACACGCGACACCCTCGAGCACCATCACCATCACCATTGA

DNA sequence of $ustD^{SA}$
ATGAAGAGCGTAGCGACGAGTTCCCTTGATGACGTAGATAAAGATTCCGTCCCCCTGGGCA
GTTCGATCAATGGCACTGCACAAGCGGAAACTCCGCTGGAGAATGTGATCGACGTCGAATC
AGTGCGCTCACATTTCCCGGTATTAGGGGGGGAAACGGCCGCGTTTAACAATGCATCAGGA
ACCGTAGTTTTGAAGGAGGCAATTGAATCGACTTCAAATTTCATGTATAGCTTTCCTTTT**TCC**
CCGGGTGTTGACGCTAAGTCAATGGAGGCTATTACCGCATATACGGGGAATAAGGGCAAGG
TTGCGGCATTTATCAATGCACTTCCTGATGAAATTACATTCGGGCAGTCCACAACTGCCCTG
TTCCGTTTATTAGGTCTGTCGCTTAAACCTATGCTGAATAACGATTGCGAAATCGTATGCTCA
ACATTATGTCACGAAGCAGCAGCTTCCGCATGGATTCATTTAAGTCGCGAATTAGGAATTAC
CATTAAGTGGTGGAGCCCAACTACTACACCGAATAGTCCCGATGATCCAGTTCTGACGACTG
ACTCATTGAAGCCCTTGCTTAGTCCAAAAACGCGCCTTGTTACATGTAATCACGTGTCGAAT
GTTGTAGGAACCATCCACCCTATTCGTGAGATTGCCGACGTGGTACATACCATTCCTGGAGC
CATGCTTATCGTTGACGGTGTGGCAAGCGTCCCGCATCGTCCAGTTGATGTTAAAGAATTGG
ATGTAGATTTTTACTGCTTTTCCTGGTACAAGTTGTTCGGACCGCATCTTGGAACCCTGTATG
CTTCCCGCAAAGCCCAAGACCGCTATATGACCTCAATTAACCATTACTTCGTCTCATCGTCG
AGCCTTGATGGTAAGCTGGCATTAGGCATGCCGTCCTTTGAACTGCAGTTGATGTGCTCTCC
AATTGTTTCGTATTTGCAAGATACGGTGGGCTGGGACCGTATCGTGCGCCAAGAGACT**GCG**
CTGGTAACTATTTTGTTGGAGTATTTACTTAGCAAGCCATCTGTATATCGTGTGTTCGGACGT
CGTAATTCTGATCCCAGTCAGCGTGTAGCAATCGTAACTTTTGAAGTCGTGGGACGTAGTTC
CGGGGATGTGGCAATGCGCGTAAATACGCGTAATCGCTTCCGCATTACCTCTGGAACCTTAA
TGGCACCGCGCCCGACATGGGACGTCTTGAAACCGAAGAGTAGCGACGGACTTGTTCGCG
TCAGCTTTGTACATTACAACACGGTTGAGGAAGTGCGTGCGTTCTGCAGCGAGTTAGACGA
GATTGTGACACGCGACACCCTCGAGCACCATCACCATCACCATTGA

DNA sequence of $ustD^{Q}$
ATGAAGAGCGTAGCGACGAGTTCCCTTGATGACGTAGATAAAGATTCCGTCCCCCTGGGCA
GTTCGATCAATGGCACTGCACAAGCGGAAACTCCGCTGGAGAATGTGATCGACGTCGAATC
AGTGCGCTCACATTTCCCGGTATTAGGGGGGGAAACGGCCGCGTTTAACAATGCATCAGGA
ACCGTAGTTTTGAAGGAGGCAATTGAATCGACTTCAAATTTCATGTATAGCTTTCCTTTT**CAG**
CCGGGTGTTGACGCTAAGTCAATGGAGGCTATTACCGCATATACGGGGAATAAGGGCAAGG
TTGCGGCATTTATCAATGCACTTCCTGATGAAATTACATTCGGGCAGTCCACAACTGCCCTG
TTCCGTTTATTAGGTCTGTCGCTTAAACCTATGCTGAATAACGATTGCGAAATCGTATGCTCA
ACATTATGTCACGAAGCAGCAGCTTCCGCATGGATTCATTTAAGTCGCGAATTAGGAATTAC
CATTAAGTGGTGGAGCCCAACTACTACACCGAATAGTCCCGATGATCCAGTTCTGACGACTG
ACTCATTGAAGCCCTTGCTTAGTCCAAAAACGCGCCTTGTTACATGTAATCACGTGTCGAAT
GTTGTAGGAACCATCCACCCTATTCGTGAGATTGCCGACGTGGTACATACCATTCCTGGAGC
CATGCTTATCGTTGACGGTGTGGCAAGCGTCCCGCATCGTCCAGTTGATGTTAAAGAATTGG
ATGTAGATTTTTACTGCTTTTCCTGGTACAAGTTGTTCGGACCGCATCTTGGAACCCTGTATG
CTTCCCGCAAAGCCCAAGACCGCTATATGACCTCAATTAACCATTACTTCGTCTCATCGTCG
AGCCTTGATGGTAAGCTGGCATTAGGCATGCCGTCCTTTGAACTGCAGTTGATGTGCTCTCC
AATTGTTTCGTATTTGCAAGATACGGTGGGCTGGGACCGTATCGTGCGCCAAGAGACTGTG
CTGGTAACTATTTTGTTGGAGTATTTACTTAGCAAGCCATCTGTATATCGTGTGTTCGGACGT
CGTAATTCTGATCCCAGTCAGCGTGTAGCAATCGTAACTTTTGAAGTCGTGGGACGTAGTTC
CGGGGATGTGGCAATGCGCGTAAATACGCGTAATCGCTTCCGCATTACCTCTGGAACCTTAA
TGGCACCGCGCCCGACATGGGACGTCTTGAAACCGAAGAGTAGCGACGGACTTGTTCGCG
TCAGCTTTGTACATTACAACACGGTTGAGGAAGTGCGTGCGTTCTGCAGCGAGTTAGACGA
GATTGTGACACGCGACACCCTCGAGCACCATCACCATCACCATTGA

DNA sequence of ustD^{QE}
ATGAAGAGCGTAGCGACGAGTTCCCTTGATGACGTAGATAAAGATTCCGTCCCCCTGGGCA
GTTCGATCAATGGCACTGCACAAGCGGAAACTCCGCTGGAGAATGTGATCGACGTCGAATC
AGTGCGCTCACATTTCCCGGTATTAGGGGGGGAAACGGCCGCGTTTAACAATGCATCAGGA
ACCGTAGTTTTGAAGGAGGCAATTGAATCGACTTCAAATTTCATGTATAGCTTTCCTTTT**CAG**
CCGGGTGTTGACGCTAAGTCAATGGAGGCTATTACCGCATATACGGGGAATAAGGGCAAGG
TTGCGGCATTTATCAATGCACTTCCTGATGAAATTACATTCGGGCAGTCCACAACTGCCCTG
TTCCGTTTATTAGGTCTGTCGCTTAAACCTATGCTGAATAACGATTGCGAAATCGTATGCTCA
ACATTATGTCACGAAGCAGCAGCTTCCGCATGGATTCATTTAAGTCGCGAATTAGGAATTAC
CATTAAGTGGTGGAGCCCAACTACTACACCGAATAGTCCCGATGATCCAGTTCTGACGACTG
ACTCATTGAAGCCCTTGCTTAGTCCAAAAACGCGCCTTGTTACATGTAATCACGTGTCGAAT
GTTGTAGGAACCATCCACCCTATTCGTGAGATTGCCGACGTGGTACATACCATTCCTGGAGC
CATGCTTATCGTTGACGGTGTGGCAAGCGTCCCGCATCGTCCAGTTGATGTTAAAGAATTGG
ATGTAGATTTTTACTGCTTTTCCTGGTACAAGTTGTTCGGACCGCATCTTGGAACCCTGTATG
CTTCCCGCAAAGCCCAAGACCGCTATATGACCTCAATTAACCATTACTTCGTCTCATCGTCG
AGCCTTGATGGTAAGCTGGCATTAGGCATGCCGTCCTTTGAACTGCAGTTGATGTGCTCTCC
AATTGTTTCGTATTTGCAAGATACGGTGGGCTGGGACCGTATCGTGCGCCAAGAGACTGTG
CTGGTAACTATTTTGTTGGAGTATTTACTTAGCAAGCCATCTGTATATCGTGTGTTCGGACGT
CGTAATTCTGATCCCAGTCAGCGTGTAGCAATCGTAACTTTTGAAGTCGTGGGACGTAGTTC
C**GAG**GATGTGGCAATGCGCGTAAATACGCGTAATCGCTTCCGCATTACCTCTGGAACCTTAA
TGGCACCGCGCCCGACATGGACGTCTTGAAACCGAAGAGTAGCGACGGACTTGTTCGCG
TCAGCTTTGTACATTACAACACGGTTGAGGAAGTGCGTGCGTTCTGCAGCGAGTTAGACGA
GATTGTGACACGCGACACCCTCGAGCACCATCACCATCACCATTGA

DNA sequence of ustD^{AIIRQ}
ATGAAGAGCGTAGCGACGAGTTCCCTTGATGACGTAGATAAAGATTCCGTCCCCCTGGGCA
GTTCGATCAATGGCACTGCACAAGCGGAAACTCCGCTGGAGAATGTGATCGACGTCGAATC
AGTGCGCTCACATTTCCCGGTATTAGGGGGGGAAACGGCCGCGTTTAACAATGCATCAGGA
ACCGTAGTTTTGAAGGAGGCAATTGAATCGACTTCAAAT**GCA**ATGTATAGCTTTCCTTTT**CAG**
CCGGGTGTT**ATC**GCTAAGTCAATGGAGGCTATTACCGCATATACGGGGAATAAGGGCAAGG
TTGCGGCATTTATCAATGCACTTCCTGATGAAATTACATTCGGGCAGTCCACAACTGCCCTG
TTCCGTTTATTAGGTCTGTCGCTTAAACCTATGCTGAATAACGATTGCGAA**ATT**GTATGCTCA
ACATTATGTCACGAAGCAGCAGCTTCCGCATGGATTCATTTAAGTCGCGAATTAGGAATTAC
CATTAAGTGGTGGAGCCCAACTACTACACCGAATAGTCCCGATGATCCAGTTCTGACGACTG
ACTCATTGAAGCCCTTGCTTAGTCCAAAAACGCGCCTTGTTACATGTAATCACGTGTCGAAT
GTTGTAGGAACCATCCACCCTATTCGTGAGATTGCCGACGTGGTACATACCATTCCTGGAGC
CATGCTTATCGTTGACGGTGTGGCAAGCGTCCCGCATCGTCCAGTTGATGTTAAAGAATTGG
ATGTAGATTTTTACTGCTTTTCCTGGTACAAGTTGTTCGGACCGCATCTTGGAACCCTGTATG
CTTCCCGCAAAGCCCAAGACCGCTATATGACCTCAATTAACCATTACTTCGTCTCATCGTCG
AGCCTTGATGGTAAGCTGGCATTAGGCATGCCGTCCTTTGAACTGCAGTTGATGTGCTCTCC
AATTGTTTCGTATTTGCAAGATACGGTGGGCTGGGACCGTATCGTGCGCCAAGAGACT**CGA**
CTGGTAACTATTTTGTTGGAGTATTTACTTAGCAAGCCATCTGTATATCGTGTGTTCGGACGT
CGTAATTCTGATCCCAGTCAGCGTGTAGCAATCGTAACTTTTGAAGTCGTGGGACGTAGTTC
C**GAG**GATGTGGCAATGCGCGTAAATACGCGTAATCGCTTCCGCATTACCTCTGGAACCTTAA
TGGCACCGCGCCCGACATGGACGTCTTGAAACCGAAGAGT**CAA**GACGGACTTGTTCGCG
TCAGCTTTGTACATTACAACACGGTTGAGGAAGTGCGTGCGTTCTGCAGCGAGTTAGACGA
GATTGTGACACGCGACACCCTCGAGCACCATCACCATCACCATTGA

DNA sequence of ustD^{7B05}
ATGAAGAGCGTAGCGACGAGTTCCCTTGATGACGTAGATAAAGATTCCGTCCCCCTGGGCA
GTTCGATCAATGGCACTGCACAAGCGGAAACTCCGCTGGAGAATGTGATCGACGTCGAATC
AGTGCGCTCACATTTCCCGGTATTAGGGGGGGAAACGGCCGCGTTTAACAATGCATCAGGA
ACCGTAGTTTTGAAGGAGGCAATTGAATCGACTTCAAAT**GCA**ATGTATAGCTTTCCTTTT**CAG**
CCGGGTGTT**ATC**GCTAAGTCAATGGAGGCTATTACCGCATATACGGGGAATAAGGGCAAGG
TTGCGGCATTTATCAATGCACTTCCTGATGAAATTACATTCGGGCAGTCCACAACTGCCCTG
TTCCGTTTATTAGGTCTGTCGCTTAAACCTATGCTGAATAACGATTGCGAA**ATT**GTATGCTCA

ACATTATGTCACGAAGCAGCAGCTTCCGCATGGATTCATTTAAGTCGCGAATTAGGAATTAC
CATTAAGTGGTGGAGCCCAACTACTACACCGAATAGTCCCGATGATCCAGTTCTGACGACTG
ACTCATTGAAGCCCTTGCTTAGTCCAAAAACGCGCCTTGTTACATGTAATCACGTGTCGAAT
GTTGTAGGAACCATCCACCCTATTCGTGAGATTGCCGACGTGGTACATACCATTCCTGGAGC
CATGCTTATCGTTGACGGTGTGGCAAGCGTCCCGCATCGTCCAGTTGATGTTAAAGAATTGG
ATGTAGATTTTTACTGCTTTTCCTGGTACAAGTTGTTCGGACCGCATCTTGGAACCCTGTATG
CTTCCCGCAAAGCCCAAGACCGCTATATGACCTCAATTAACCATTACTTCGTCTCATCGTCG
AGCCTTGATGGTAAGCTGGCATTAGGCATGCCGTCCTTTGAACTGCAGTTGATGTGCTCTCC
AATTGTTTCGTATTTGCAAGATACGGTGGGCTGGGACCGTATCGTGCGCCAAGAGACT**CGA**
CTGGTAACTATTTTGTTGGAGTATTTACTTAGCAAGCCATCTGTATATCGTGTGTTCGGACGT
CGTAATTCTGATCCCAGTCAGCGTGTAGCAATCGTAACTTTTGAAGTCGTGGGACGTAGTTC
C**GAG**GATGTGGCAATGCGCGTAAATACGCGTAATCGCTTCCGCATTACCTCTGGA**TCC**TTA**T**
TTGCACCGCGCCCGACATGGGACGTCTTGAAACCGAAGAGT**CAA**GACGGACTTGTTCGCGT
CAGCTTTGTACATTACAACACGGTTGAGGAAGTGCGTGCGTTCTGCAGCGAGTTAGACGAG
ATTGTGACACGCGACACCCTCGAGCACCATCACCATCACCATTGA

DNA sequence of ustD^{7G11}
ATGAAGAGCGTAGCGACGAGTTCCCTTGATGACGTAGATAAAGATTCCGTCCCCCTGGGCA
GTTCGATCAATGGCACTGCACAAGCGGAAACTCCGCTGGAGAATGTGATCGACGTCGAATC
AGTGCGCTCACATTTCCCGGTATTAGGGGGGGAAACGGCCGCGTTTAACAATGCATCAGGA
ACCGTAGTTTTGAAGGAGGCAATTGAATCGACTTCAAAT**GCA**ATGTATAGCTTTCCTTTT**CAG**
CCGGGTGTT**ATC**GCTAAGTCAATGGAGGCTATTACCGCATATACGGGGAATAAGGGCAAGG
TTGCGGCATTTATCAATGCACTTCCTGATGAAATTACATTCGGGCAGTCCACAACTGCCCTG
TTCCGTTTATTAGGTCTGTCGCTTAAACCTATGCTGAATAACGATTGCGAA**ATT**GTATGCTCA
ACATTATGTCACGAAGCAGCAGCTTCCGCATGGATTCATTTAAGTCGCGAATTAGGAATTAC
CATTAAGTGGTGGAGCCCAACTACTACACCGAATAGTCCCGATGATCCAGTTCTGACGACTG
ACTCATTGAAGCCCTTGCTTAGTCCAAAAACGCGCCTTGTTACATGTAATCACGTGTCGAAT
GTTGTAGGAACCATCCACCCTATTCGTGAGATTGCCGACGTGGTACATACCATTCCTGGAGC
CATGCTTATCGTTGACGGTGTGGCAAGCGTCCCGCATCGTCCAGTTGATGTTAAAGAATTGG
ATGTAGATTTTTACTGCTTTTCCTGGTACAAGTTGTTCGGACCGCATCTTGGAACCCTGTATG
CTTCCCGCAAAGCCCAAGACCGCTATATGACCTCAATTAACCATTACTTCGTCTCATCGTCG
AGCCTTGATGGTAAGCTGGCATTAGGC**GTG**CCGTCCTTTGAACTGCAGTTGATGTGCTCTCC
AATTGTTTCGTATTTGCAAGATACGGTGGGCTGGGACCGTATCGTGCGCCAAGAGACT**CGA**
CTGGTAACTATTTTGTTGGAGTATTTACTTAGCAAGCCATCTGTATATCGTGTGTTCGGACGT
CGTAATTCTGATCCCAGTCAGCGTGTAGCAATCGTAACTTTTGAAGTCGTGGGACGTAGTTC
C**GAG**GATGTGGCAATGCGCGTAAATACGCGTAATCGCTTCCGCATTACCTCTGGA**TCC**TTA**T**
GGGCACCGCGCCCGACATGGGACGTCTTGAAACCGAAGAGT**CAA**GACGGACTTGTTCGCG
TCAGCTTTGTACATTACAACACGGTTGAGGAAGTGCGTGCGTTCTGCAGCGAGTTAGACGA
GATTGTGACACGCGACACCCTCGAGCACCATCACCATCACCATTGA

Primer Sequences: All primers were purchased from Integrated DNA Technologies

Protein	Forward Primer (5' to 3')	Reverse Primer (5' to 3')
pET22b(+)-UstD$^{2.0}$	GAAATAATTTTGTTTAACTTTAAG AAGGAGATATACATATG	GCCGGATCTCAATGGTGATGGT GATGGTGCTCGAG
D86X	TTTCCTTTTCAGCCGGGTGTT**XX** **X**GCTAAGTCAATGGAGGC	AACACCCGGCTGAAAAGGAA
F75X	GGCAATTGAATCGACTTCAAAT**X** **XX**ATGTATAGCTTTCCTTTTCCC CC	ATTTGAAGTCGATTCAATTGCCT CCTTC
G101X	ACCGCATATACGGGGAATAAG**X** **XX**AAGGTTGCGGCATTTATCAAT GC	CTTATTCCCCGTATATGCGGTAA TAGCCTC
G373X	GAAGTCGTGGGACGTAGTTCC**X** **XX**GATGTGGCAATGCGCGTAAAT ACG	GGAACTACGTCCCACGACTTCAA AAGTTA

I141X	CTATGCTGAATAACGATTGCGAA**XXX**GTATGCTCAACATTATGTCACGAAG	TTCGCAATCGTTATTCAGCATAGGTTTAA
K342X	GTTGGAGTATTTACTTAGC**XXX**CCATCTGTATATCGTGTGTTCGG	GCTAAGTAAATACTCCAACAAAATAGTTACC
M299X	GATGGTAAGCTGGCATTAGGC**XXX**CCGTCCTTTGAACTGCAGTTGA	GCCTAATGCCAGCTTACCATCAAGGC
P80X	CAAATTTCATGTATAGCTTT**XXX**TTTCCCCCGGGTGTTGAC	AAAGCTATACATGAAATTTGAAGTCGATTC
P82X	CATGTATAGCTTTCCTTTT**XXX**CCGGGTGTTGACGCTAAGTC	AAAAGGAAAGCTATACATGAAATTTGAAGTCG
P83X	TTTCATGTATAGCTTTCCTTTTCAG**XXX**GGTGTTGACGCTAAGTCAATGGAGGC	CTGAAAAGGAAAGCTATACATGAAATTTGAAGTCG
S407X	CGTCTTGAAACCGAAGAGT**XXX**GACGGACTTGTTCGCGTCAG	ACTCTTCGGTTTCAAGACGTCC
V330X	GTATCGTGCGCCAAGAGACT**XXX**CTGGTAACTATTTTGTTGGAG	AGTCTCTTGGCGCACGATACGGTCCC
Y277X	CCCGCAAAGCCCAAGACCGC**XXX**ATGACCTCAATTAACCATTACTTCG	GCGGTCTTGGGCTTTGCGGG
Y418X	GTTCGCGTCAGCTTTGTACAT**XXX**AACACGGTTGAGGAAGTGCG	ATGTACAAAGCTGACGCGAACAAG
Y96X	GTCAATGGAGGCTATTACCGCA**XXX**ACGGGGAATAAGGGCAAGGT	TGCGGTAATAGCCTCCATTG
F75[GYA]	GGCAATTGAATCGACTTCAAAT**GYA**ATGTATAGCTTTCCTTTTCAGCC	ATTTGAAGTCGATTCAATTGCCTCCTTC
D86[RWC]	GCTTTCCTTTTCAGCCGGGTGTT**RWC**GCTAAGTCAATGGAGGCTATTAC	AACACCCGGCTGAAAAGGAA
I141[RTK]	CCTATGCTGAATAACGATTGCGAA**RTK**GTATGCTCAACATTATGTCACGAAGC	TTCGCAATCGTTATTCAGCATAGGTTTAA
V330[SNA]	CCGTATCGTGCGCCAAGAGACT**SNA**CTGGTAACTATTTTGTTGGAGTATTTAC	AGTCTCTTGGCGCACGATACGGTCCC
S407[SAA]	CGTCTTGAAACCGAAGAGT**SAA**GACGGACTTGTTCGCGTCAGC	ACTCTTCGGTTTCAAGACGTCC
S407[DCA]	CGTCTTGAAACCGAAGAGT**DCA**GACGGACTTGTTCGCGTCAGC	ACTCTTCGGTTTCAAGACGTCC
TTLM_4 mutation	CGCGTAATCGCTTCCGCATT**RYA**TCTGGA**HYCDYAWKK**GCACCGCGCCCGACATGGGACG	AATGCGGAAGCGATTACGCGTATTTACGC
TTLM_3 mutation_T388	CGCGTAATCGCTTCCGCATTACCTCTGGA**HYCDYAWKK**GCACCGCGCCCGACATGGGACG	AATGCGGAAGCGATTACGCGTATTTACGC
TTLM_3 mutation_T391	CGCGTAATCGCTTCCGCATT**RYA**TCTGGAACC**DYAWKK**GCACCGCGCCCGACATGGGACG	AATGCGGAAGCGATTACGCGTATTTACGC
TTLM_3 mutation_L392	CGCGTAATCGCTTCCGCATT**RYA**TCTGGA**HYC**TTA**WKK**GCACCGCGCCCGACATGGGACG	AATGCGGAAGCGATTACGCGTATTTACGC

TTLM_3 mutation_M393	CGCGTAATCGCTTCCGCATT**RYA**TCTGGA**HYCDYA**ATGGCACCGCGCCCGACATGGGACG	AATGCGGAAGCGATTACGCGTATTTACGC
M299 [DTG]	GATGGTAAGCTGGCATTAGGC**DTG**CCGTCCTTTGAACTGCAGTTG	GCCTAATGCCAGCTTACCATC
H263X	CCTGGTACAAGTTGTTCGGACCG**XXX**CTTGGAACCCTGTATGCTTCCCGC	CGGTCCGAACAACTTGTACCAGGAAAAGC
H283X	CCAAGACCGCTATATGACCTCAATTAAC**XXX**TACTTCGTCTCATCGTCGAGCCTTG	
F285X	GCTATATGACCTCAATTAACCATTAC**XXX**GTCTCATCGTCGAGCCTTGATGGTAAGCT	GTAATGGTTAATTGAGGTCATATAGCGG
P300X-7B05	GATGGTAAGCTGGCATTAGGCATG**XXX**TCCTTTGAACTGCAGTTGATGTGCTC	CATGCCTAATGCCAGCTTACCATCA
P300X-7G11	GATGGTAAGCTGGCATTAGGCGTG**XXX**TCCTTTGAACTGCAGTTGATGTGCTC	CACGCCTAATGCCAGCTTACCATCA
S389X-7B05	TACGCGTAATCGCTTCCGCATTACC**XXX**GGATCCTTATTTGCACCGCGCCCG	GGTAATGCGGAAGCGATTACGCGTA
S389X-7G11	TACGCGTAATCGCTTCCGCATTACC**XXX**GGATCCTTATGGGCACCGCGCCCG	GGTAATGCGGAAGCGATTACGCGTA

* **XXX** indicates a 22-codon library made as a mixture of 3 degenerate codon primers (NDT, VHG, TGG), as described by Kille, S. et al.[1]

Protein Expression & Purification

Optimized Expression of UstD$^{2.0}$ and variants. An overnight culture of *E. coli* BL21(DE3) harboring a pET-22b(+) plasmid encoding a given UstD$^{2.0}$ variant was created by inoculating 10 mL of TB_{amp} media with a single colony. This culture was shaken at 37 ^oC and 200 rpm for ~16 h. 10 mL of overnight culture was then used to inoculate 1 L of TB_{amp}, which was shaken at 37 ^oC and 200 rpm for approximately 2.5 h or until an optical density (OD) of 0.4-0.6 was reached. Cultures were removed from the incubator and cooled on ice for 30 min, followed by induction with 100 µM IPTG. The cultures were allowed to continue growing for an additional ~16 h at 20 ^oC with shaking at 180 rpm. Cells were then harvested by centrifugation (4 ^oC, 30 min, 4,000 *xg*), and the resulting cell pellets were stored at -20 ^oC overnight.

Protein Purification. To purify UstD, cell pellets were thawed at room temperature and then resuspended in lysis buffer, comprised of enzyme storage buffer (100 mM potassium phosphate buffer pH 7.0, 100 mM sodium chloride), 20 mM imidazole, 1 mg/mL Hen Egg White Lysozyme (GoldBio), 0.2 mg/mL DNase (GoldBio), 1 mM $MgCl_2$, and 0.5 mg/g cell pellet pyridoxal 5'-phosphate (PLP). A ratio of 4 mL lysis buffer per gram of wet cell pellet was used. Cells lysis began by shaking for 1 h at 37 ^oC and 200 rpm. The resuspended cells were subsequently sonicated (30 s per g cell pellet, 2 s on, 2 s off, 40% amplitude). The resulting lysate was then clarified at 48,384 x*g* for 20 min. The resulting supernatant was purified using Ni/NTA beads pre-equilibrated in storage buffer. 1-2 mL of resin were used per 50 mL of lysis. The flow-through was re-passed once prior to washing. The collected beads were washed with 5 column volumes each of storage buffer containing 20 mM, 40 mM, and 60 mM imidazole. Protein was eluted with 3 column volumes of storage buffer containing 250 mM imidazole and the flow-through was collected until the eluent was no longer yellow (color due to the enzymatically bound PLP

cofactor). Imidazole was then removed using a PD10 salt exchange column pre-equilibrated in storage buffer.

Protein Characterization and Storage

Concentration measurement. Enzyme concentration was determined by Bradford assay, using bovine serum albumin (BSA) for a standard concentration curve.

Gel Electrophoresis. Protein purity was analyzed by sodium dodecyl sulfate-polyacrylamide gel electrophoresis (SDS-PAGE) using 12% polyacrylamide gels.

Storage. Purified enzyme was flash frozen in pellet form by pipetting enzyme dropwise into a crystallization dish filled with liquid nitrogen. The enzyme was transferred to a plastic conical and stored at -80 ^{o}C until further use. Frozen pellets were thawed at room temperature and centrifuged before use.

Library Generation for Directed Evolution

Production of UstD random mutagenesis libraries. Random mutagenesis was carried out via error-prone PCR. Reaction conditions were optimized to generate 1-2 coding mutations per plasmid. Reactions were set-up by adding the following to a PCR tube: 5 µL 10x Taq buffer (New England Biolabs), 1 µL 10 mM dNTP mix, 1 µL 10 µM 22b-intF, 1 µL 10 µM 22b-intR, 1 µL ~100 ng/µL parent plasmid, 5.5 µL 50 µM $MgCl_2$, 7.5 µL 100 µM $MnCl_2$, 1 µL DMSO, 0.5 µL Taq polymerase (New England Biolabs) and diluted to a total volume of 50 µL with milliQ H_2O. Reactions were carried out in a thermocycler according to the following scheme:

Thermocycler program
Step 1: 95 °C 2 min 30 s
Step 2: 95 °C 15 s
Step 3: 54 °C 20 s
Step 4: 68 °C 1 min 45 s
Step 5: 68 °C 5 min
Extension steps 2 – 4 were performed for 30 cycles.

The PCR product was purified using a preparative agarose gel. The purified DNA fragment was inserted into a pET-22b(+) vector by the Gibson Assembly method.[2] BL21 (DE3) *E. coli* cells were subsequently transformed with the resulting cyclized DNA product via electroporation. After 45 min of recovery in Terrific Broth (TB) media at 37 °C, 200 rpm, cells were plated onto LB plates with 100 µg/mL Ampicillin (amp) and incubated overnight. Single colonies were used to inoculate 5 mL TB + 100 µg/mL amp (TB_{amp}), which were grown overnight at 37 °C, 200 rpm. Colonies were sequenced and there was an average of 2 coding mutations.

Production of UstD degenerate codon libraries. Primers containing degenerate codons were purchased from IDT and are listed above. Mutagenesis was carried out via overlap-extension PCR. Reactions were set-up by adding the following to a PCR tube: 10 µL 5x HF buffer (New England Biolabs), 1 µL 10 mM dNTP mix, 1 µL 10 µM forward primer, 1 µL 10 µM reverse primer, 1 µL ~100 ng/µL parent plasmid, 1 µL DMSO, 1 µL Phusion polymerase (New England Biolabs) and diluted to a total volume of 50 µL with milliQ H_2O. Reactions were carried out in a thermocycler according to the following scheme:

Thermocycler program
Step 1: 98 °C 1 min
Step 2: 98 °C 15 s
Step 3: 54 °C 20 s
Step 4: 72 °C 1 min
Step 5: 72 °C 5 min

Extension steps 2 – 4 were performed for at least 30 cycles.

The PCR product was purified using a preparative agarose gel. The purified DNA fragment was inserted into a pET-22b(+) vector by the Gibson Assembly method as described above.[2]

Enzymatic Activity Experiments

General procedure for library generation and screening. Mutagenized plasmid DNA was generated and transformed into electrocompetent BL21(DE3) *E. coli* cells as described above. A 96-well plate containing 600 µL of TB_{amp} per well was inoculated with single colonies from the transformed library. Each 96-well plate included parent positive controls (from a fresh transformation), negative controls and a sterile control that was not inoculated. These starter plates were grown overnight at 37 °C, 200 rpm. Glycerol stocks of each starter plate were made following overnight growth using 150 µL of the remaining culture and 100 µL of 50% sterile glycerol to ensure the sequence of any mutants of interest could be determined.

Expression plates were prepared with 600-610 µL of TB_{amp} per well and inoculated with 6-20 µL of overnight culture. The expression plates were grown at 37 °C, 200 rpm for 3 h. Expression plates were then placed on ice for 30 min. Cultures were induced with a final concentration of 0.1 mM IPTG. The expression culture was grown overnight at 20 °C, 200 rpm. Following overnight growth, the plate was centrifuged (4,000 x*g,* 30 min, 4 ^{o}C) and all media was removed by striking plates against a paper towel on a table. Expression plates were stored at -20 °C until further use.

A reaction master mix containing a final concentration of 50 mM L-asp, 50 µM PLP, and buffer (100 mM KPi + NaCl, pH 7.0) was added to thawed expression pellets using an Opentrons OT-2 liquid handling robot. The pellets were resuspended by vortexing. Then, 50 mM final concentration of electrophile mix (substrates varied throughout evolution), was added to the reaction mixture by Opentrons OT-2 robot and reactions were allowed to incubate at 37 °C, 200 rpm for the desired reaction time (1-8 h). Subsequently, reactions were quenched with 300 µL (1 reaction volume) acetonitrile using Opentrons OT-2 robot and clarified at 4,000 x*g* for 30 min. The supernatant was transferred to a 0.2 µm centrifuge filter plate (PALL) and filtered at 1,500 rpm for 10 min into a clean Waters 96-well UPLC plate for analysis by UPLC-MS.

Specific library generation and screening conditions for global random mutagenesis and F75X, P80X, P82X, and G373X site saturation libraries. Library generation and screening follows the general procedure laid out above. The parent enzyme was **UstD$^{2.0}$** for these libraries. Expression plates were prepared with **600 µL** of TB_{amp} per well and inoculated with **6 µL** of overnight culture. After induction, all expression plate wells had a final volume of **700 uL**. The enzymatic reaction time was **1 h** at 37 °C, 200 rpm with a 50 mM final concentration electrophile mix consisting of **4.2 mM 1a**, **4.2 mM 1b**, **and 41.6 mM 1c**. The ketone:aldehyde ratio is **5:1** for these libraries (Figure 2A, S18, S19).

Specific library generation and screening conditions for P83X, D86X, Y96X, G101X, I141X, Y277X, M299X, V330X, K342X, S407X, Y418X site saturation libraries. Library generation and screening follows the general procedure laid out above. The parent enzyme was **QE** for these libraries. Expression plates were prepared with **600 µL** of TB_{amp} per well and inoculated with **6 µL** of overnight culture. After induction, all expression plate wells had a final volume of **700 uL**. The enzymatic reaction time was **1 h** at 37 °C, 200 rpm with a 50 mM final concentration electrophile mix consisting of **5 mM 1b, 10 mM 1c, 2.5 mM 1d, 32.5 mM 1e**. The ketone:aldehyde ratio is **9:1** for these libraries (Figure S5-6, S9).

Specific library generation and screening conditions for distal recombination library. Library generation and screening follows the general procedure laid out above. The parent enzyme was **QE** for these libraries. Expression plates were prepared with **600 µL** of TB_{amp} per well and inoculated with **6 µL** of overnight culture. After induction, all expression plate wells had a final volume of **700 uL**. The enzymatic reaction time was **8 h** at 37 °C, 200 rpm with a 50 mM final

concentration electrophile mix consisting of **5 mM 1b, 10 mM 1c, 2.5 mM 1d, 32.5 mM 1e**. The ketone:aldehyde ratio is **9:1** for these libraries (Figure S8, Table S1).

Specific library generation and screening conditions for active site mutagenesis libraries. Library generation and screening follows the general procedure laid out above. For DNA generation, an equimolar primer mix of TTLM_4 mutation, TTLM_3 mutation_T388, TTLM_3 mutation_T391, TTLM_3 mutation_L392, TTLM_3 mutation_M393 was used as a doping strategy to lower the mutagenesis rate to 3.3 mutations per variant. All other primers were used in the standard fashion associated with overlap extension PCR. The parent enzyme was **AIIRQ** for these libraries. Expression plates were prepared with **610 µL** of TB_{amp} per well and inoculated with **20 µL** of overnight culture. After induction, all expression plate wells had a final volume of **700 uL**. The enzymatic reaction time was **6 h** at 37 °C, 200 rpm with a 50 mM final concentration electrophile mix consisting of **5 mM 1b, 10 mM 1c, 2.5 mM 1d, 32.5 mM 1e**. The ketone:aldehyde ratio is **9:1** for these libraries (Figure S10, Table S2).

Analysis of library screens. The relative amount of product formation compared to the parent reactions were measured by single ion retention mass analysis via UPLC-MS. To be considered a "hit" a variant usually displayed at least a 1.5-fold increase in product formation or a shift of $\Delta Spec^{app}$ (described below) of 0.5.

Discussion and calculation of $\Delta Spec^{app}$. The global random mutagenesis libraries revealed variants with changes in promiscuity, but not increases in total activity. Therefore, analysis of the libraries was based on changes in promiscuity. This analysis was originally performed 'by eye' and we subsequently constructed a simple metric that captures changes in reactivity.
While there are many was that can be used to calculate promiscuity of an enzyme, we found they had limited utility when applied directly to our data.[3,4] Therefore, we opted for a simple calculation of the changes in the "apparent specificity" of the variants compared to parent, as follows:

Starting from the traditional Michaelis-Menten equation (Eq. 1):

$$v_0 = \frac{k_{cat} * [E_T] * [S]}{K_M + [S]}$$ **Eq. 1**

Where v_0 = initial velocity, $[E_T]$ = total enzyme concentration, $[S]$ = concentration of substrate, and K_M = Michaelis-Menten constant. For a unimolecular, irreversible reaction (Eq. 2), if $k_2 << k_1$, we can approximate $k_{cat} = k_2$.

$$E + S \underset{k_{-1}}{\overset{k_1}{\rightleftharpoons}} ES \xrightarrow{k_2}$$ **Eq. 2**

For unimolecular reactions under initial velocity conditions with equimolar substrates (S_A and S_B) in competition (Eq. 3), the observed product abundances (P_A and P_B) are directly proportional to the catalytic efficiencies (Eq. 4).[5]

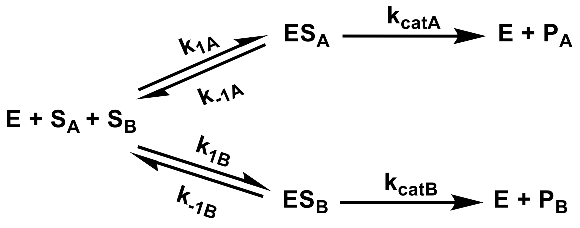

Eq. 3

$$\frac{v_{PA}}{v_{PB}} = \frac{[S_A] * \frac{k_{catA}}{K_{MA}}}{[S_B] * \frac{k_{catB}}{K_{MB}}} \qquad \textbf{Eq. 4}$$

Therefore, the enzyme specificity (Spec) for P_A can be calculated (Eq. 5) when S_A and S_B are present in equimolar amounts.

$$Spec\ P_A = \frac{P_A}{P_A + P_B} \qquad \textbf{Eq. 5}$$

However, screening conditions can deviate far from initial velocity and first order conditions. We therefore pursued calculation of an apparent specificity with the following assumptions. For reactions conducted here, reactions were saturated in aspartate and therefore pseudo-first order in each electrophile. For the global random mutagenesis screens, three substrates were added in different concentrations, roughly inversely to their electrophilicity. We therefore normalized for these differences and calculated: (Eq. 6).

$$P_A = \frac{I_{PA}}{[S_A]} \qquad \textbf{Eq. 6}$$

Where I_{PA} is the integrated ion count associated with product A obtained from the UPLC. Initial velocity conditions were not maintained during screening since any change in sequence or expression level has the potential to change the initial velocity regime. We calculated each enzyme's apparent specificity ($Spec^{app}$) for each of the three possible products (Eq. 7).

$$Spec^{app}\ P_A = \frac{P_A}{P_A + P_B + P_c} \qquad \textbf{Eq. 7}$$

The $Spec^{app}$ is a lower bound on enzyme specificity, since the distribution of products at high conversion tends towards the stoichiometry of the substrates. The ratios of the variant activity compared to parent activity were calculated for each product (Eq. 8).

$$Ratio\ P_A = \frac{Variant\ Spec^{app} P_A}{Parent\ Spec^{app} P_A} \qquad \textbf{Eq. 8}$$

We note this calculation makes no assumption about the relative ionization efficiencies of the products. The ratio here mitigates much of the potential deviation between substrates, as the ionization efficiency for a given substrate is present in both the numerator and denomenter. Finally, the change in the apparent specificity ($\Delta Spec^{app}$) was calculated for each enzyme (Eq. 9).

$$\Delta Spec^{app} = |\log(Ratio\ P_A)| + |\log(Ratio\ P_B)| + |\log(Ratio\ P_C)| \qquad \textbf{Eq. 9}$$

By using the log of the ratio for each product, we ensure that increases and decreases in activity are weighted equally. Using this metric, we determined that any variant with $\Delta Spec^{app} > 0.5$ had altered promiscuity. However, this type of metric was very noisy when applied to low-intensity measurements and we therefore excluded from our promiscuity analysis any variants with less than 20% of total parent activity. In the future, for other directed evolution campaigns, these cutoffs will doubtlessly vary based on assay sensitivity and the number of substrates.

Validations

Every validated mutant of interest was heterologous expressed and used as whole cell catalyst, cell lysate or Ni-NTA purified enzyme. Reactions were performed on single electrophile substrates in duplicate or triplicate. A complete overview of all the rounds of directed evolution can be found in Table S3.

Validations of F75A. A reaction master mix (225 µL) containing a final concentration of 50 mM L-asp, 50 µM PLP, and buffer (100 mM KPi + NaCl, pH 7.0) was added to epi tubes. **1c** (15 µL, 50 mM final concentration, 5% DMSO in reaction mixture) was added to the epi tubes. Frozen cell stocks of *E. coli* cells expressing UstD$^{2.0}$+F75A were thawed to room temperature. The reaction was initiated upon addition of 60 µL of cell suspension (20 mg/mL final concentration). The reactions were allowed to incubate at 37 °C, 200 rpm for 1 h. Subsequently, reactions were quenched with 300 µL (1 reaction volume) acetonitrile and clarified at 16160 x*g* for 10 min. The supernatant was transferred to a UPLC vial and analyzed by UPLC-MS. The reactions were done in triplicate technical replicates at the same time as parent to determine the fold change most accurately (Figure S20).

Validations of G373E, P82Q, QE. A reaction master mix containing a final concentration of 50 mM L-asp, 5 µM PLP, and buffer (100 mM KPi + NaCl, pH 7.0) was added to epi tubes. The electrophile **1c** (30 µL, 50 mM final concentration, 5% DMSO in reaction mixture) was added to the epi tubes. The enzyme catalyst was thawed to room temperature and clarified at 16160 x*g* for 3 min. The reaction was initiated upon addition of enzyme (0.01 mol% catalyst, 10,000 Max TON). The reactions were allowed to incubate at 37 °C overnight. Subsequently, reactions were quenched with 300 µL (1 reaction volume) acetonitrile and clarified at 16160 x*g* for 10 min. The supernatant was transferred to a UPLC plate and analyzed by UPLC-MS. The reactions were done in duplicate technical replicates at the same time as parent to determine the fold change most accurately (Figure S2).

Validations of P83T, P83Y, P83V, P83G, D86I, D86V, Y96V, Y96F, G101R, G101Q, G101A+ΔH445, Y277H, Y277F+W399C. A reaction master mix containing a final concentration of 50 mM L-asp, 50 µM PLP, and buffer (100 mM KPi + NaCl, pH 7.0) was added to epi tubes. The electrophile (15 µL, 50 mM final concentration, 5% DMSO in reaction mixture) was added to the epi tubes. The reaction was initiated upon addition of lysate (60 µL, 40 - 50 mg/mL final concentration). The reactions were allowed to incubate at 37 °C overnight. Subsequently, reactions were quenched with 300 µL (1 reaction volume) acetonitrile and clarified at 4300 rpm for 15 min. The supernatant (250 µL) was transferred to a filter plate (0.2 µm filter) and centrifuged at 1000 rpm into a UPLC plate and analyzed by UPLC-MS. The reactions were done in duplicate or triplicate technical replicates at the same time as parent to determine the fold change most accurately (Figure S7).

Validations of distal recombination and active site variants. A reaction master mix containing a final concentration of 50 mM L-asp, 5 µM PLP, and buffer (100 mM KPi, pH 7.0, 100 mM NaCl) was added to epi tubes. The electrophile (10 µL, 50 mM final concentration, 5% DMSO in reaction mixture) was added to the epi tubes. The enzyme catalyst was thawed to room temperature and clarified at 16160 x*g* for 3 min. The reaction was initiated upon addition of enzyme (0.01 mol% catalyst, 10,000 Max TON). The reactions were allowed to incubate at 37 °C for 16 h. Subsequently, reactions were quenched with 100 µL (1 reaction volume) acetonitrile and clarified at 4000 x*g* for 15 min. The supernatant was diluted and filtered through a 0.2 µm PALL filter into a UPLC plate and analyzed by UPLC-MS. The reactions were done in triplicate technical replicates at the same time as parent to determine the fold change most accurately. To analyze each reaction by UPLC, the enzymatic reactions were diluted. The **1b** reactions were diluted 50x. **1c** reactions were diluted 3.3x. **1e** reactions were diluted 3.3x. **1d** reactions were diluted 100x (Figure S8, S11).

Reaction Condition Optimization

We optimized reaction conditions by observing the progress curve of a **1c** reaction with 7G11 in varying conditions. It was known to us that PLP is degraded over the course of the reaction via off-pathway deamination of L-alanine which is formed as a shunt product via protonation of the nucleophilic enamine intermediate. We hypothesized that increasing the amount of PLP in the reaction would allow for higher yield of the product. Therefore, we observed

the reaction progress at varying amounts of PLP (10x, 20x, 50x compared to enzyme loading). While each reaction appeared to reach the same yield, the 50x PLP reaction achieved the max yield and then **2c** was degraded in the reaction time. This result indicated to us that there was enough PLP in the reaction to form and degrade the desired product indicating a sufficient excess for enzyme catalysis. Therefore, we elected to run reactions at 50x PLP for unactivated ketones. Next, we varied the concentration of L-asp (50 mM, 100 mM, 250 mM) in the reaction mixture to attempt to competitively inhibit product re-entry and degradation. Any amount of additional L-asp increased the yield of the amino acid product and kept degradation from occurring (Figure S12).

We repeated the same experiments using **1d** as the substrate and observed a worse yield with 50x PLP compared to 10x PLP. Therefore, we chose to use 10x PLP for highly activated ketone substrates. Addition of 250 mM L-asp resulted in higher yield of **2d**. Therefore, we determined our standard conditions to be 250 mM L-asp, 50 mM ketone, 10x PLP for activated substrates, 50x PLP for unactivated substrates, 4 hour reaction time, 37 °C, 5% co-solvent, 100 mM KPi pH 7.0, 100 mM NaCl (Figure S12).

Lineage analysis

General Procedure. All reactions were done in triplicate on analytical scale (200 µL). The buffer used for all enzymatic reactions was 100 mM KPi, pH 7.0, 100 mM NaCl. PLP and L-aspartate stock solutions were made in water. Electrophile stock solutions were made in DMSO and each reaction only has a single electrophile. The concentration of all UstD variants were quantified by Bradford assay prior to reactions and all enzyme stocks were diluted to the same concentration. All samples were analyzed following Marfey's derivatization by Waters Acquity UPLC-MS using a BEH C18 column (Waters). To correct for small deviations in injection volume, an internal derivatization standard was included (0.098 mM L-arginine). Derivatized amino acid product quantitation was performed by integrating chromatograph peaks at 340 nm and corrected using the internal standard peak area. To calculate product concentrations, a standard curve was generated by subjecting stock solutions of L-phenylalanine (0.4 mM–50 mM) in water to the identical procedure used to process and derivatize enzymatic reaction solutions. These curves were used to calculate the concentrations of UstD products in solution, and subsequently total turnover numbers (TTN).

Enzymatic reactions. Electrophile (10 µmol, 1 equiv., 50 mM final concentration, 5% DMSO final concentration) was added to each well of a 96-well plate. A reaction master mixture containing L-aspartate (50 µmol, 5 equiv., 250 mM final concentration), pyridoxal-5'-phosphate (10 or 50-molar equivalents to final enzyme concentration, see Table S4) and buffer was aliquoted into each of the wells. The plate was vortexed gently to mix. Reactions were initiated by addition of UstD (0.1-0.003 mol% catalyst, 1,000-30,000 max turnover number, see Table S4) to bring the total reaction volume to 200 µL. The 96-well plate was sealed with a silicon lid and placed at 37 °C for 4 h. Reactions were quenched with 200 µL of acetonitrile (1 reaction volume) and diluted with 200 µL of 1:1 ACN:DI H_2O to homogenize reaction solutions. Denatured enzyme was removed by passing the supernatant through a 0.2 µm PALL filter plate by centrifugation at 2000 rpm for 10 min. Marfey's derivatization of the clarified enzymatic reactions was performed to quantify amino acid yield, results shown in Figure 5, Table S5, S21-24.

Marfey's derivatization procedure. To a fresh 96-well plate, 10 µL of quenched reaction mixture (2.7 mM total amines in reaction, 1 equiv., ~0.8 µmol total amines in reaction) 140 µL of 10.41 mM $NaHCO_3$ with 0.21 mM of L-arginine as an internal standard (9.7 mM $NaHCO_3$ final concentration, 3.5 equiv. base, 2.9 µmol $NaHCO_3$), and 10 mM L-FDAA (5 mM final concentration, 1.8 equiv., 1.5 µmol) were added in each well. The derivatization reaction was allowed to proceed at 37 °C for 18 h. The reactions were quenched with 300 µL of 60 mM HCl in acetonitrile (1 reaction volume) and analyzed via UPLC-MS no later than 24 h after quenching. Note that the amino acid products are susceptible to form dehydration products such as a lactone or alkene upon addition of the acid required to quench the reactions. This results in product peaks with masses differing by 18 mass units. The linear amino acid product will display major ions for both the molecular ion and a dehydrated ion (-18 mass units), while the dehydration products will

only display a major ion associated with dehydration (-18 mass units). Turnover numbers were calculated based on the total integration of linear and dehydrated amino acid product peaks at 340 nm.

Analytical substrate scope

General Procedure. All reactions were done only once on analytical scale (200 µL) and followed the general procedure used for the lineage analysis above.

Enzymatic reactions. An Eppendorf tube was charged with electrophile (10 µmol, 1 equiv., 50 mM final concentration, 5% DMSO final concentration). Then, L-aspartate (50 µmol, 5 equiv., 250 mM final concentration), pyridoxal-5'-phosphate (trifluoromethyl ketones reactions used 10-molar equivalents all others used 50-molar equivalents to final enzyme concentration), and buffer were aliquoted into the tube. Reactions were initiated by addition of enzyme (0.1 mol% catalyst, 1,000 max turnover number) to bring the total reaction volume to 200 µL. The reactions were placed at 37 °C for 4 h. Reactions were quenched with 200 µL of acetonitrile (1 reaction volume) and diluted with 200 µL of 1:1 ACN:DI H_2O to homogenize reaction solutions. Denatured enzyme was removed by centrifugation at 16160 *xg* for 5 min. Marfey's derivatization of the clarified enzymatic reactions were performed to quantify amino acid yield as described above (Figure S14).

Preparative Scale *in vitro* Biocatalytic Reactions

Procedure P1, preparative scale production of unprotected γ-hydroxy amino acids. A 100-mL round bottom flask was charged with a given ketone (0.5 mmol, 1.0 equiv, 50 mM final concentration), which was then dissolved in an appropriate amount of MeOH (5% v/v final concentration). This solution was then diluted with 100 mM potassium phosphate buffer (pH 7.0) containing 100 mM sodium chloride. L-aspartate sodium salt monohydrate (2.5 mmol, 5.0 equiv, 250 mM final concentration) and 10-50 molar equivalents of pyridoxal-5'-phosphate (PLP) relative to final enzyme concentration were then added, followed by addition of 7G11 or 7B05 (0.1% mol cat). The total reaction volume was 10 mL. Note, we recommend this specific order of addition for the substrates for best results. The reaction flask was placed in the dark at 37 ^{o}C for 4 h. Product formation was monitored by UPLC-MS. After reaction completion, the reaction mixture was quenched with an equivalent volume of acetonitrile (ACN) and centrifuged (4,000 rpm, 15 min) to remove aggregated protein. The decanted supernatant was then concentrated to ~2 mL by rotary evaporation and loaded onto a preparative reverse-phase C18 column pre-equilibrated at 1% methanol:water. Purification was performed via gradient elution on an Isolera One Flash Purification system (Biotage). Fractions bearing product (confirmed by UPLC-MS sampling of fraction tubes) were pooled and dried by rotary evaporation. The product was then resuspended in a minimal quantity of water, transferred to a pre-weighed 20-mL vial, frozen, and lyophilized. The d.r. and e.e. were evaluated by Marfey's derivatization as shown in Figs. S25-35.

Procedure P2, preparative scale production of Fmoc-protected 2l. A 100-mL round bottom flask was charged with **1l** (0.5 mmol, 1.0 equiv, 50 mM final concentration), which was then dissolved in an appropriate amount of MeOH (5% v/v final concentration). This solution was then diluted with 100 mM potassium phosphate buffer (pH 7.0) containing 100 mM sodium chloride. L-aspartate sodium salt monohydrate (2.5 mmol, 5.0 equiv, 250 mM final concentration) and 50 molar equivalents of pyridoxal-5'-phosphate (PLP) relative to final enzyme concentration were then added, followed by addition of 7G11 (0.1% mol cat). The total reaction volume was 10 mL. Note, we recommend this specific order of addition for the substrates for best results. The reaction flask was placed in the dark at 37 ^{o}C for 4 h. Product formation was monitored by UPLC-MS. After reaction completion, the reaction mixture was quenched with an equivalent volume of acetonitrile (ACN) and centrifuged (4,000 rpm, 15 min) to remove aggregated protein. The supernatant was collected in a 250-mL round bottom and basified to pH ~10 using 6 M NaOH. Then Fmoc-Cl (3.7 mmol, 1.5 equiv of total amines) was dissolved in 10 mL ACN and added to the round bottom. The reaction was allowed to stir at room temperature for 4 h. The ACN was removed from the reaction by rotary evaporation. The resulting aqueous layer was acidified to pH

~ 3 using HCl. An extraction of the resulting solution was performed with EtOAc (3x25 mL). The combined organic layers were washed with saturated $NaHCO_3$ (2x25 mL), then saturated NaCl (2x25 mL), and finally dried over $MgSO_4$. The organic layer was gravity filtered then concentrated to dryness and redissolved in a minimum amount of DCM. The resulting solution was loaded onto a preparative normal-phase silica column. Purification was performed via gradient elution (hexane:EtOAc) on an Isolera One Flash Purification system (Biotage). Fractions bearing product (confirmed by UPLC-MS sampling of fraction tubes) were pooled and dried by rotary evaporation. The product was transferred to a pre-weighed vial, and dried on high vacuum. The e.e. was evaluated by Marfey's derivatization as shown in Figs. S36-37.

Procedure P3, preparative scale production of Fmoc-protected 2m. A 100-mL round bottom flask was charged with **1m** (0.5 mmol, 1.0 equiv, 50 mM final concentration), which was then dissolved in an appropriate amount of MeOH (5% v/v final concentration). This solution was then diluted with 100 mM potassium phosphate buffer (pH 7.0) containing 100 mM sodium chloride. L-aspartate sodium salt monohydrate (5 mmol, 10.0 equiv, 250 mM final concentration) and 50 molar equivalents of pyridoxal-5'-phosphate (PLP) relative to final enzyme concentration were then added, followed by addition of 7G11 (0.4% mol cat). The total reaction volume was 20 mL. Note, we recommend this specific order of addition for the substrates for best results. The reaction flask was placed in the dark at 37 °C for 4 h. Product formation was monitored by UPLC-MS. After reaction completion, the reaction mixture was quenched with an equivalent volume of acetonitrile (ACN) and centrifuged (4,000 rpm, 15 min) to remove aggregated protein. The supernatant was collected in a 250-mL round bottom and basified to pH ~10 using 300 µL of 6 M NaOH. Then Fmoc-Cl (3.7 mmol, 0.74 equiv of total amines, 93.7 mM final concentration) was added and the reaction was allowed to stir at room temperature for 4 h, final pH = 6. The ACN was removed from the reaction by rotary evaporation. The resulting aqueous layer was acidified to pH ~ 4 using HCl which caused the formation of a gel. Addition of EtOAc (100 mL) to the heterogeneous solution and vigorous stirring completely dissolved the solid. An extraction of the resulting solution was performed with EtOAc (3x50 mL). The combined organic layers were concentrated to dryness and redissolved in a minimum amount of DCM. The resulting solution was loaded onto a preparative normal-phase silica column. Purification was performed via gradient elution (hexane:EtOAc) on an Isolera One Flash Purification system (Biotage). Fractions bearing product (confirmed by UPLC-MS sampling of fraction tubes) were pooled and dried by rotary evaporation. The product was transferred to a pre-weighed vial, and dried on high vacuum. The e.e. was evaluated by Marfey's derivatization as shown in Figs. S38-39.

Characterization of γ-hydroxy amino acid products

2d – Synthesis of (2*S*,4*R*)-2-amino-4-benzyl-5,5,5-trifluoro-4-hydroxypentanoic acid

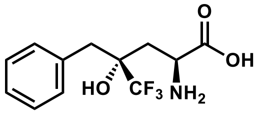

Prepared from **1d** using procedure **P1**. **Enzyme:** 7B05 **Isolated yield:** 46%, d.r. >20:1, e.r >99:1
^{1}H NMR (500 MHz, Deuterium Oxide) δ 7.45 – 7.35 (m, 5H), 3.56 (dd, *J* = 7.7, 6.0 Hz, 1H), 3.19 (d, *J* = 14.1 Hz, 1H), 3.08 (d, *J* = 14.1 Hz, 1H), 2.17 (dd, *J* = 15.1, 6.3 Hz, 1H), 2.03 (dd, *J* = 15.2, 7.7 Hz, 1H).**^{13}C NMR** (126 MHz, Deuterium Oxide) δ 178.9, 135.0, 131.1, 128.4, 127.3, 126.8 (q, *J* = 286.3 Hz), 75.1 (q, *J* = 26.6 Hz), 51.7, 40.2, 34.4.**^{19}F NMR** (377 MHz, D_2O) δ -79.58. **HRMS (ESI):** $[M-H]^-$ calcd. for $C_{11}H_{12}F_3NO_3$, 262.0697; found, 262.0696.

2f – Synthesis of (2*S*,4*S*)-2-amino-5-(furan-2-yl)-4-hydroxy-4-methylpentanoic acid

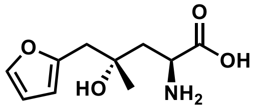

Prepared from **1f** using procedure **P1**. **Enzyme:** 7G11 **Isolated yield:** 39%, d.r. >20:1, e.r. >99:1 **^{1}H NMR** (500 MHz, Deuterium Oxide) δ 7.50 (d, *J* = 1.2 Hz, 1H), 6.47 (dd, *J* = 3.2, 1.9 Hz, 1H), 6.30 (d, *J* = 3.1 Hz, 1H), 3.83 (dd, *J* = 8.3, 4.8 Hz, 1H), 2.96 (dd, *J* = 16.9, 2.1 Hz, 2H), 2.13 (dd, *J* = 15.0, 4.8 Hz, 1H), 1.86 (dd, *J* = 15.0, 8.4 Hz, 1H), 1.29 (s, 3H). **^{13}C NMR** (126 MHz, D_2O) δ 177.9, 152.0, 142.1, 110.6, 108.5, 72.7, 52.6, 41.9, 39.1, 26.7. **HRMS (ESI):** $[M-H]^-$ calcd. for $C_{10}H_{15}NO_4$, 212.0928; found, 212.0925.

2h – Synthesis of (2*S*,4*S*)-2-amino-5,5,5-trifluoro-4-hydroxy-4-phenylpentanoic acid

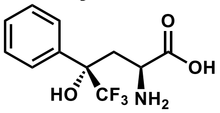

Prepared from **1h** using procedure **P1**. **Enzyme:** 7G11 **Isolated yield:** 80%, d.r. >20:1, e.r. >99:1 **^{1}H NMR** (500 MHz, Deuterium Oxide) δ 7.74 (d, *J* = 7.6 Hz, 1H), 7.62 – 7.50 (m, 2H), 3.31 – 3.24 (d, *J* = 9.0 Hz, 1H), 2.84 – 2.76 (d, *J* = 14.9 Hz, 1H), 2.35 (dd, *J* = 14.8, 11.0 Hz, 1H). **^{13}C NMR** (126 MHz, Deuterium Oxide) δ 177.93, 136.39, 129.07, 128.82, 126.75, 125.11 (q, *J* = 285.1) , 77.77 (q, *J* = 28.3 Hz), 52.09, 35.74. **^{19}F NMR** (377 MHz, Deuterium Oxide) δ -80.02. **HRMS (ESI):** $[M-H]^-$ calcd. for $C_{12}H_{14}F_3NO_3$, 276.0853; found, 276.0855. **Note:** Isolated yield is corrected for minor, known impurities.

2i – Synthesis of (2*S*,4*S*)-2-amino-4-((*R*)-2,3-dihydrobenzo[*b*][1,4]dioxin-2-yl)-4-hydroxypentanoic acid

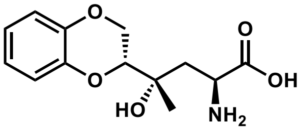

Prepared from **1i** using procedure **P1**. **Enzyme:** 7G11 **Isolated yield:** 31%, d.r. = 18:1, e.r. >99:1 **^{1}H NMR** (500 MHz, Deuterium Oxide:MeOH-*d^{4}*) δ 7.02 – 6.96 (m, 1H), 6.95 – 6.87 (m, 3H), 4.53 (dd, *J* = 10.8, 1.6 Hz, 1H), 4.14 – 4.01 (m, 2H), 3.76 (dd, *J* = 8.2, 5.2 Hz, 1H), 2.33 (dd, *J* = 14.9, 5.2 Hz, 1H), 1.81 (dd, *J* = 14.9, 8.2 Hz, 1H), 1.32 (s, 3H). **^{13}C NMR** (126 MHz, Deuterium Oxide:MeOH-*d^{4}*) δ 179.7, 144.3, 143.8, 123.0, 122.7, 118.3, 117.8, 78.8, 73.2, 65.6, 53.1, 40.7, 23.4. **HRMS (ESI):** $[M-H]^-$ calcd. for $C_{13}H_{17}NO_5$, 266.1034; found, 266.1034.

2j – Synthesis of (2*S*)-2-amino-3-(3-hydroxy-7-methyl-3,4-dihydro-2*H*-benzo[*b*][1,4]dioxepin-3-yl)propanoic acid

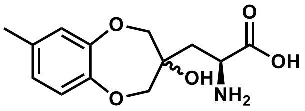

Prepared from **1j** using procedure **P1**. **Enzyme:** 7G11 **Isolated yield:** 96%, d.r. = 1:1, e.r. >99:1 **^{1}H NMR** (500 MHz, Deuterium Oxide:MeOH-*d^{4}*) δ 6.90 (dd, *J* = 8.0, 3.9 Hz, 1H), 6.86 – 6.80 (m, 2H), 4.18 – 4.02 (m, 4H), 3.69 (dd, *J* = 8.7, 4.8 Hz, 1H), 2.24 (s, 3H), 2.21 (dd, *J* = 15.0, 4.8 Hz, 1H), 1.87 (dd, *J* = 15.0, 8.5 Hz, 1H). **^{13}C NMR** (126 MHz, Deuterium Oxide:MeOH-*d^{4}*) δ 181.0, 152.38, 152.36, 150.44, 150.41, 137.0, 127.2, 123.94, 123.92, 123.36, 123.34, 80.7, 80.5, 79.7, 79.5, 76.7, 54.6, 39.4, 22.4. **HRMS (ESI):** $[M-H]^-$ calcd. for $C_{13}H_{17}NO_5$, 266.1034; found, 266.1033.

2k – Synthesis of (2*S*)-2-amino-3-(3-hydroxytetrahydrothiophen-3-yl)propanoic acid

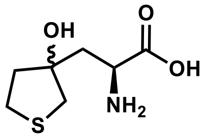

Prepared from **1k** using procedure **P1**. **Enzyme:** 7G11 **Isolated yield:** 59%, d.r. = 1:1, e.r. >99:1 **^{1}H NMR** (500 MHz, Deuterium Oxide) δ 3.89 – 3.80 (m, 1H), 3.07 – 2.86 (m, 4H), 2.39 – 2.09 (m, 3H), 1.95 (dddd, *J* = 12.9, 10.1, 8.1, 2.0 Hz, 1H). **^{13}C NMR** (126 MHz, D_2O) δ 176.8, 176.7, 83.2, 82.9, 53.76, 53.71, 42.3, 42.1, 41.0, 40.8, 39.6, 28.25, 28.21. **HSQC:** note a high-resolution HSQC is included to differentiate ^{13}C signals for each diastereomer. **HRMS (ESI):** $[M-H]^-$ calcd. for $C_7H_{13}NO_3S$, 192.0689; found, 192.0687. **Note:** Isolated yield is corrected for known impurities.

2l – Synthesis of ((*S*)-2-((((9*H*-fluoren-9-yl)methoxy)carbonyl)amino)-3-(3-hydroxyoxetan-3-yl)propanoic acid

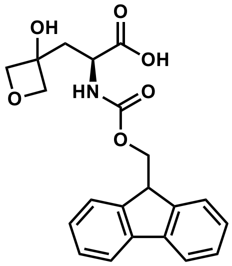

Prepared from **1l** using procedure **P2**. **Enzyme:** 7G11 **Isolated yield:** 62%, e.r. = 98:2 **^{1}H NMR** (500 MHz, DMSO-*d*$_6$) δ 7.93 – 7.86 (m, 3H), 7.68 (d, *J* = 7.5 Hz, 2H), 7.42 (t, *J* = 7.5 Hz, 2H), 7.34 (t, *J* = 7.4 Hz, 2H), 4.75 (d, *J* = 7.8 Hz, 1H), 4.68 – 4.62 (m, 2H), 4.60 (d, *J* = 7.8 Hz, 1H), 4.45 (dd, *J* = 18.1, 9.4 Hz, 1H), 4.39 – 4.31 (m, 2H), 4.23 (t, *J* = 6.7 Hz, 1H), 2.86 (dd, *J* = 13.0, 9.5 Hz, 1H), 2.32 (dd, *J* = 13.0, 10.0 Hz, 1H). **^{13}C NMR** (126 MHz, DMSO) δ 173.9, 155.6, 143.72, 143.67, 140.7, 127.6, 127.1, 125.09, 125.05, 120.1, 82.0, 80.9, 80.7, 65.7, 49.7, 46.6, 35.9. **HRMS (ESI):** $[M-H]^-$ calcd. for $C_{21}H_{21}NO_6$, 382.1296; found, 382.1299. **Note:** Isolated yield is corrected for known impurities.

2m – (*S*)-2-((((9*H*-fluoren-9-yl)methoxy)carbonyl)amino)-4,5-dihydroxy-4-(hydroxymethyl)pentanoic acid

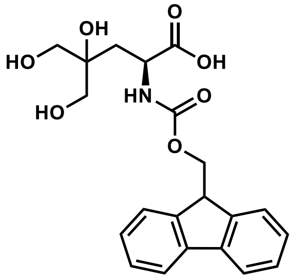

Prepared from **1m** using procedure **P3**. **Enzyme:** 7G11 **Isolated yield:** 71%, e.r. = 99:1 **^{1}H NMR** (500 MHz, Methanol-*d*$_4$) δ 7.79 (d, *J* = 7.5 Hz, 2H), 7.65 (d, *J* = 7.5 Hz, 2H), 7.39 (t, *J* = 7.5 Hz, 2H), 7.31 (t, *J* = 7.1 Hz, 2H), 4.66 (t, *J* = 10.2 Hz, 1H), 4.41 – 4.32 (m, 2H), 4.22 (t, *J* = 7.0 Hz, 1H), 3.75 (d, *J* = 12.0 Hz, 1H), 3.68 – 3.58 (m, 3H), 2.52 (dd, *J* = 12.7, 10.0 Hz, 1H), 2.13 (dd, *J* = 12.7, 10.5 Hz, 1H). **^{13}C NMR** (126 MHz, MeOD) δ 177.4, 158.3, 145.22, 145.20, 142.6, 128.8, 128.2, 126.22, 126.21, 120.9, 87.9, 68.1, 65.7, 65.5, 52.6, 48.3, 33.4. **HRMS (ESI):** $[M-H_2O+NH_4]^+$ calcd. for $C_{21}H_{23}NO_7$, 401.1707; found, 401.1700. $[M-H_2O+CH_3CO_2]^-$ calcd. for $C_{21}H_{23}NO_7$, 442.1507; found, 442.1508. **Notes:** Isolated yield is corrected for known impurities.

Ammonium acetate was added to samples during preparation for collecting HRMS data, the adducts detected reflect this addition.

NMR Spectra

2d

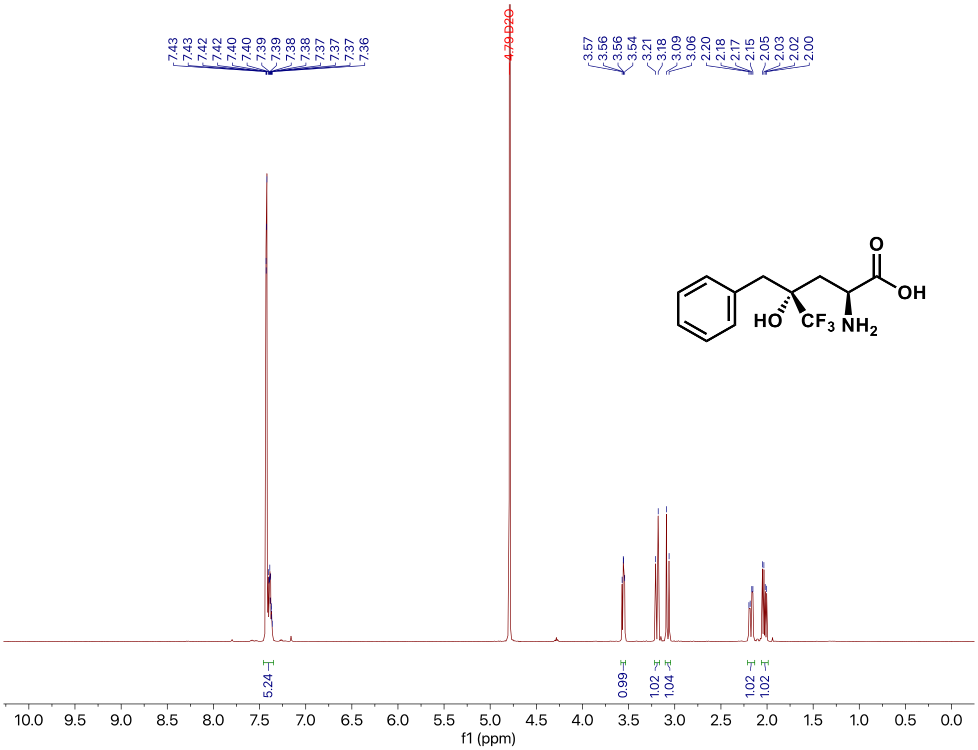

^{1}H NMR spectrum in D_2O at 500 MHz

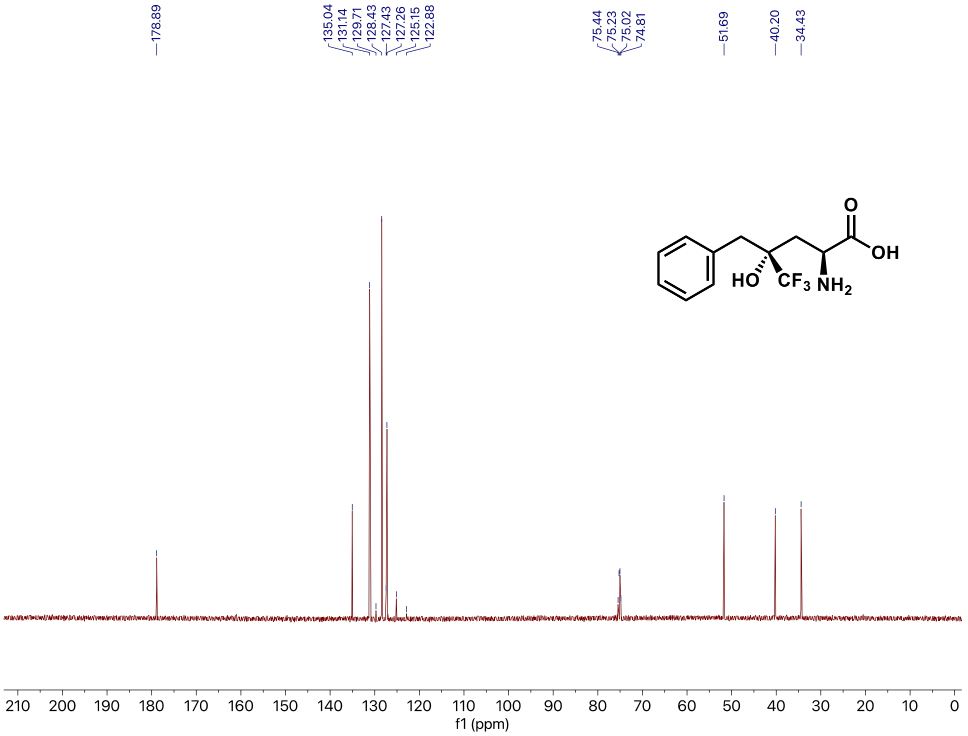

^{13}C NMR spectrum in D_2O at 126 MHz

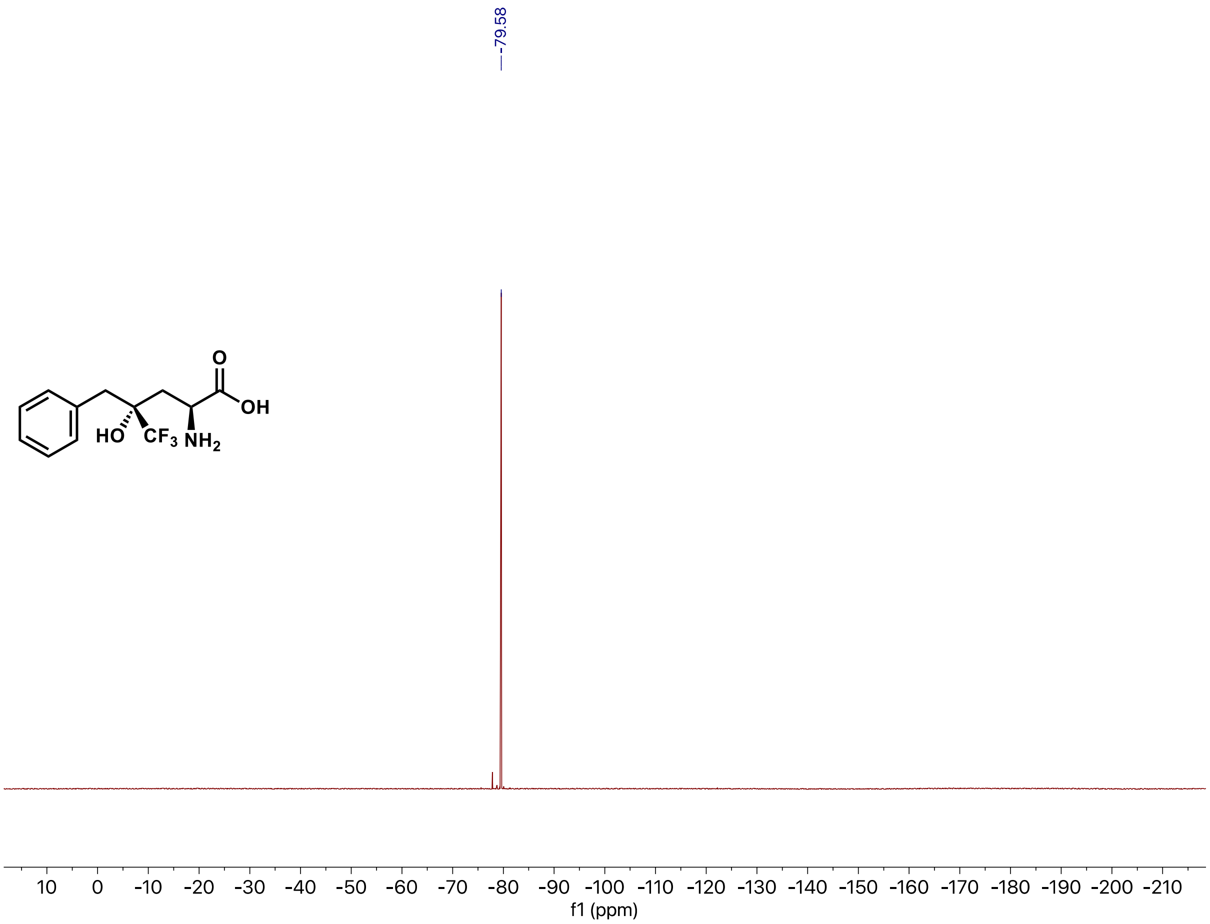

^{19}F NMR spectrum in D_2O at 377 MHz

2f

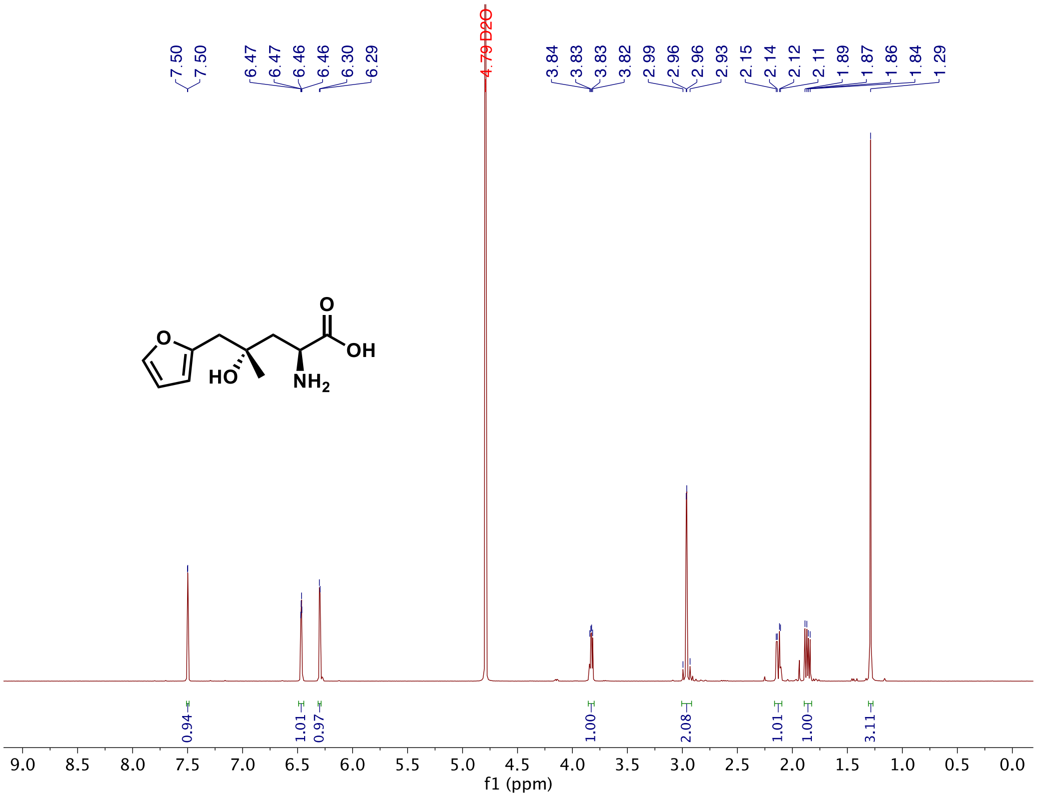

^{1}H NMR spectrum in D_2O at 500 MHz

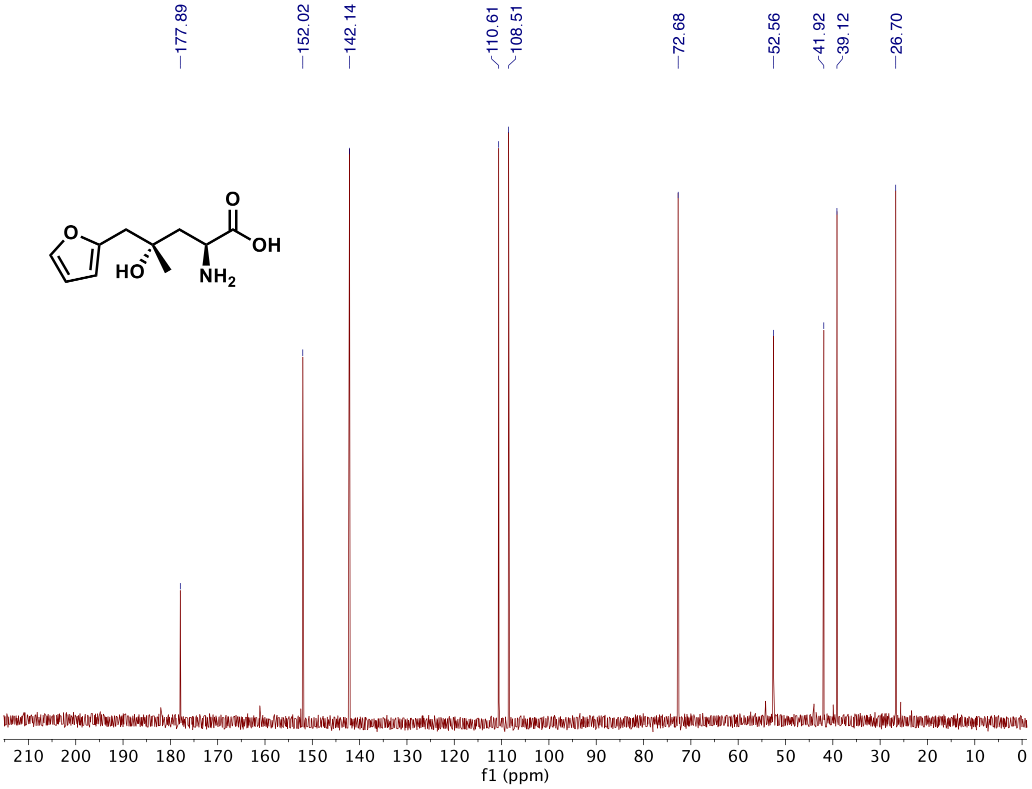

^{13}C NMR spectrum in D_2O at 126 MHz

2h

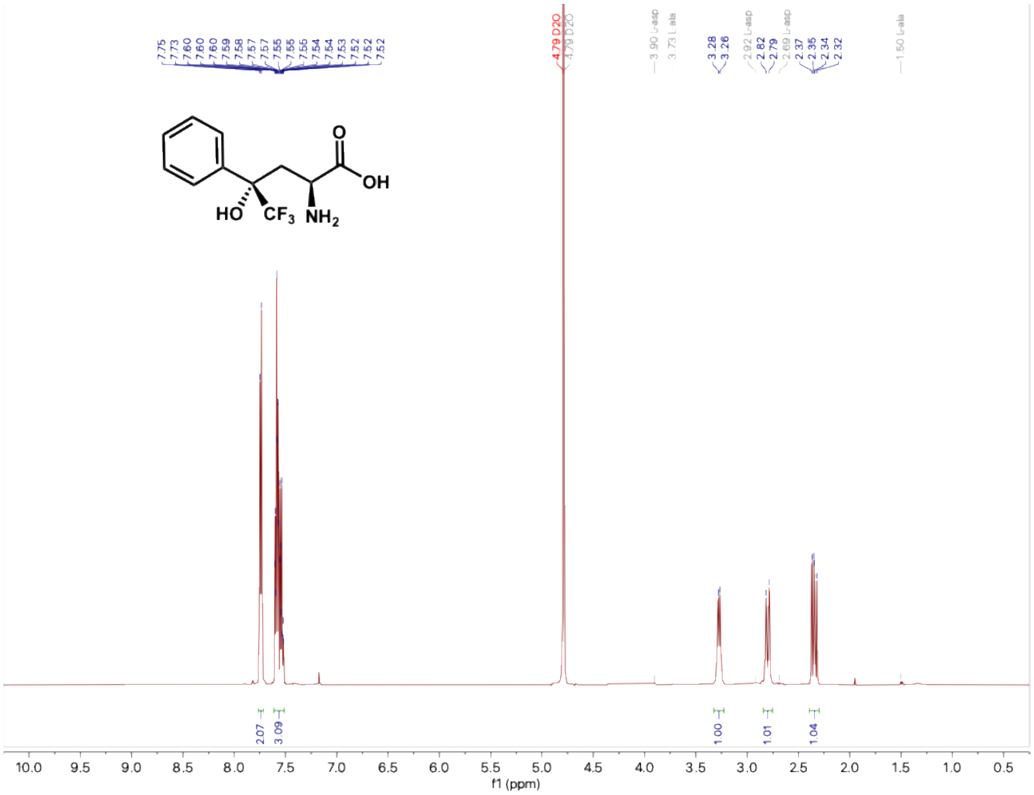

^1H NMR spectrum in D_2O at 500 MHz

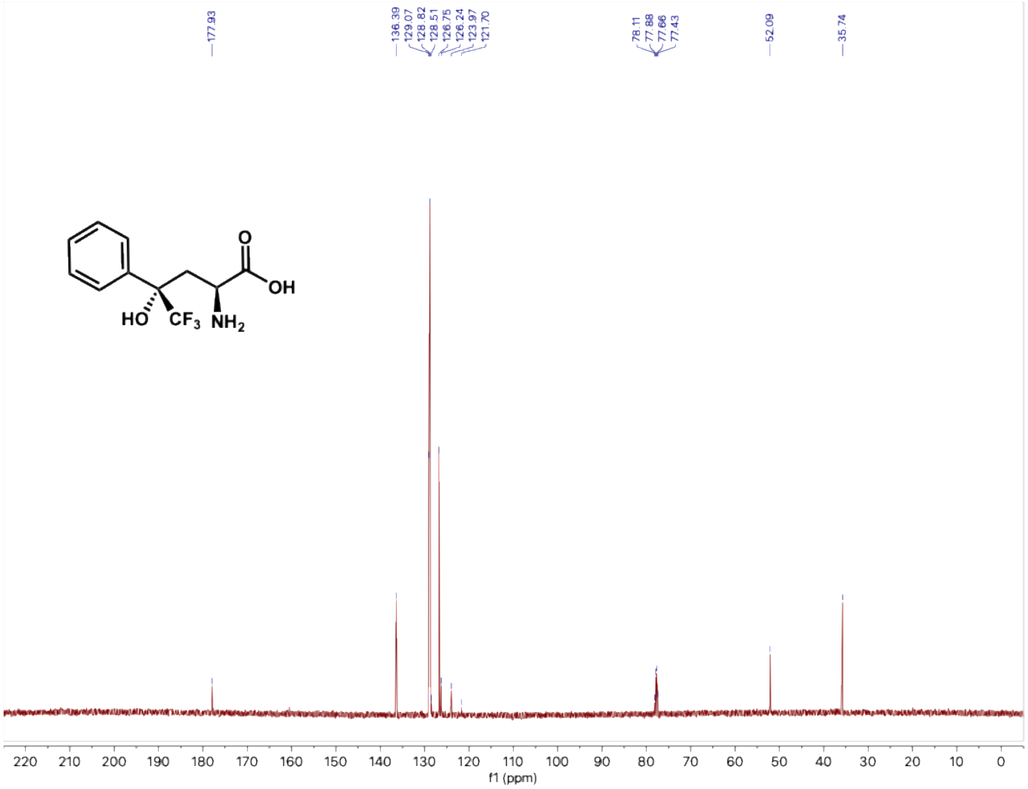

^{13}C NMR spectrum in D_2O at 126 MHz

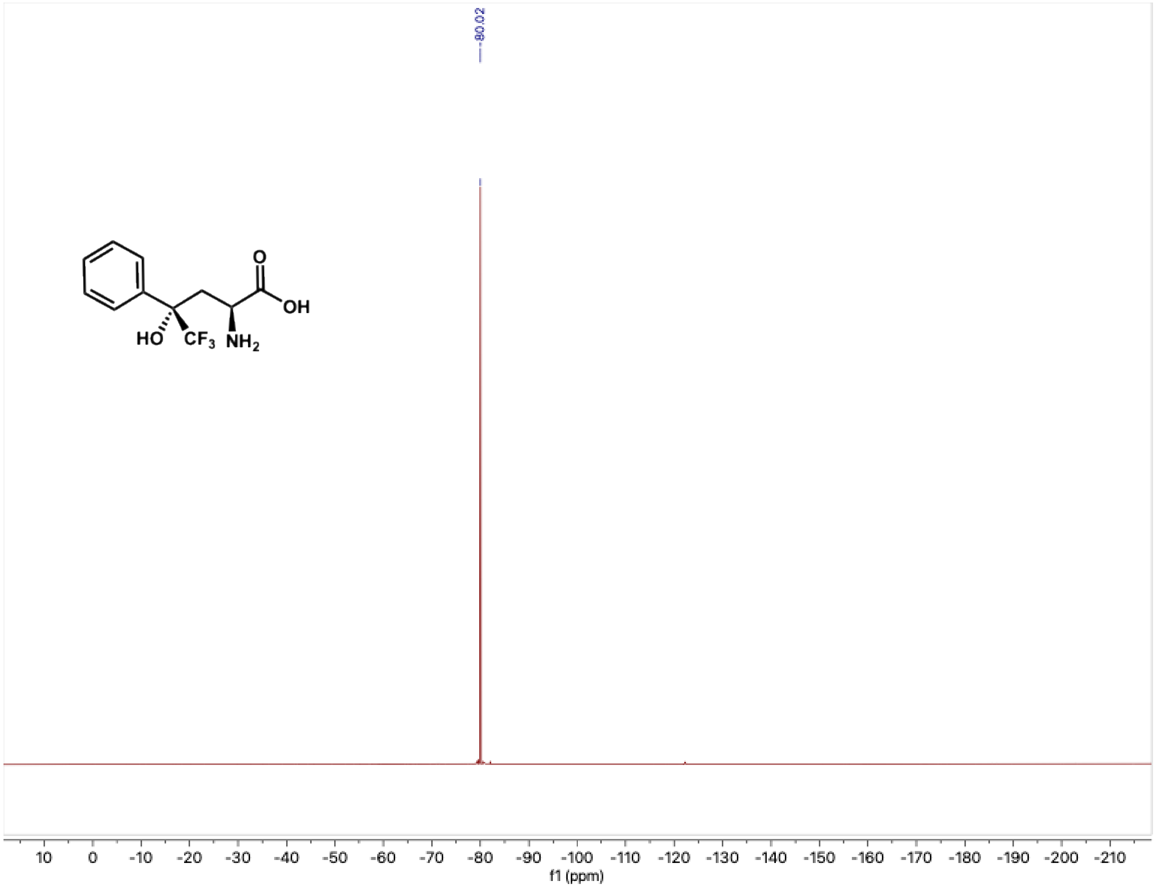

^{19}F NMR spectrum in D_2O at 377 MHz

2i

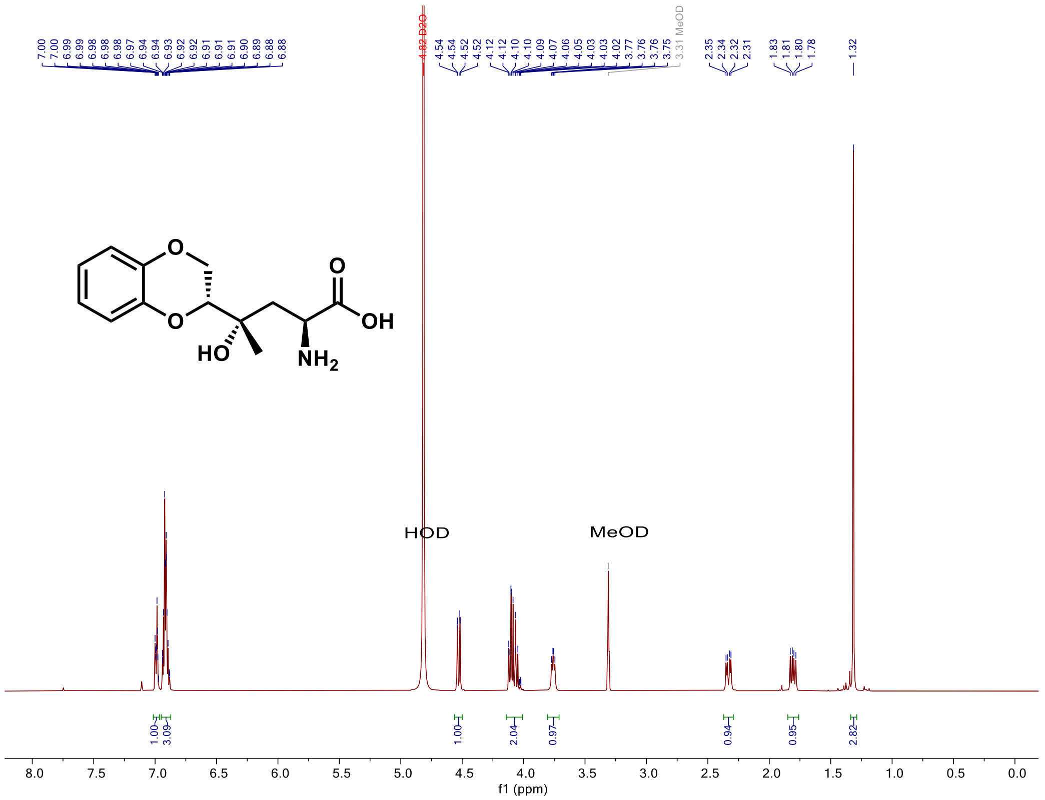

^{1}H NMR spectrum in D_2O:MeOD-*d*^{4} at 500 MHz

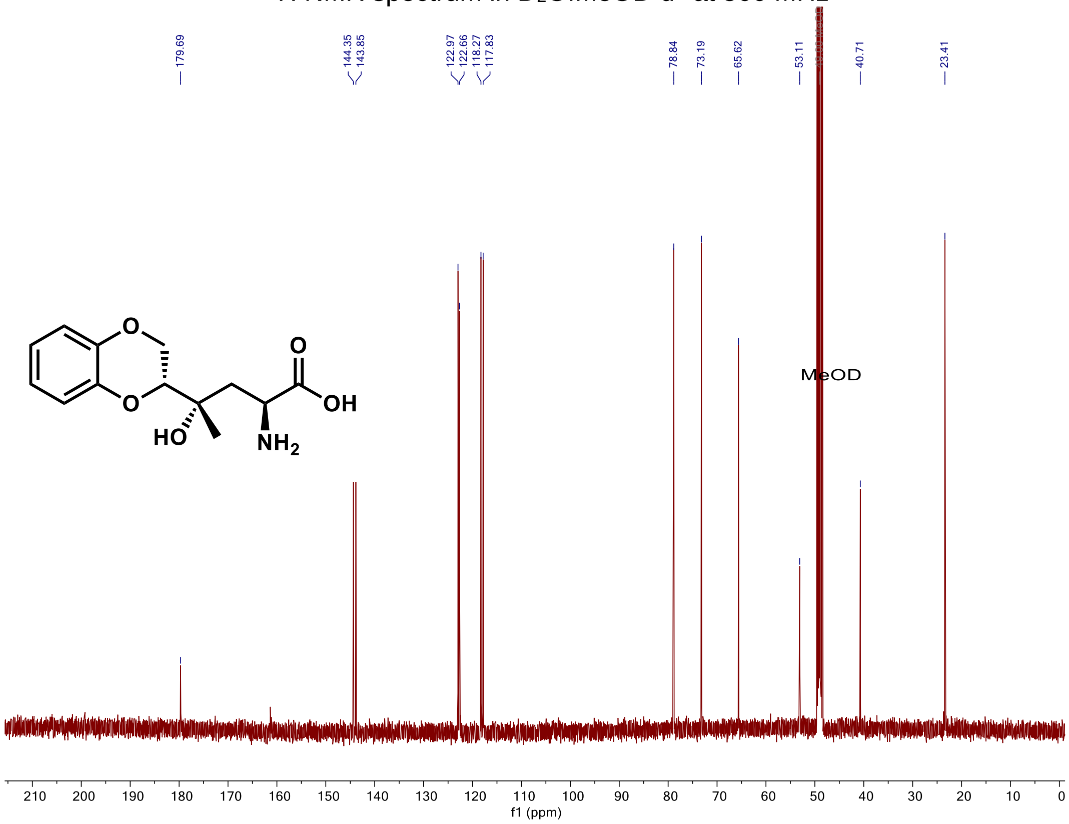

^{13}C NMR spectrum in D_2O:MeOH-*d*^{4} at 126 MHz

2j

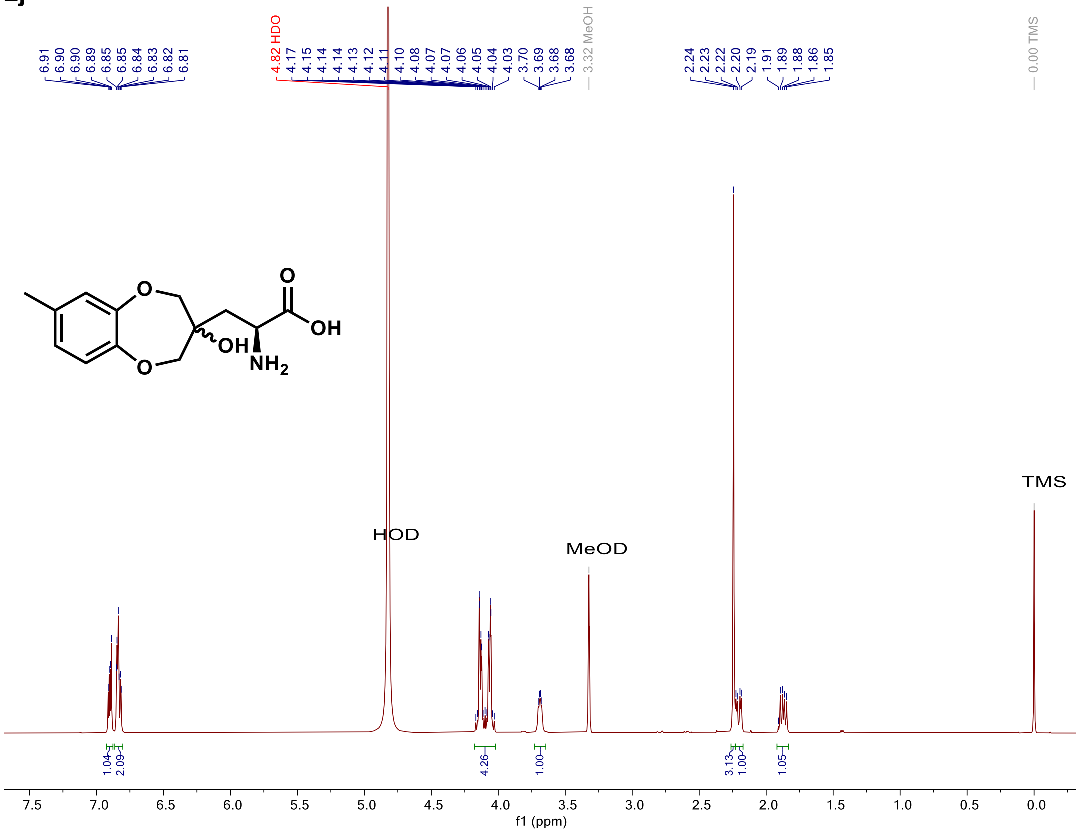

^1H NMR spectrum in D_2O:MeOH-d^4 at 500 MHz

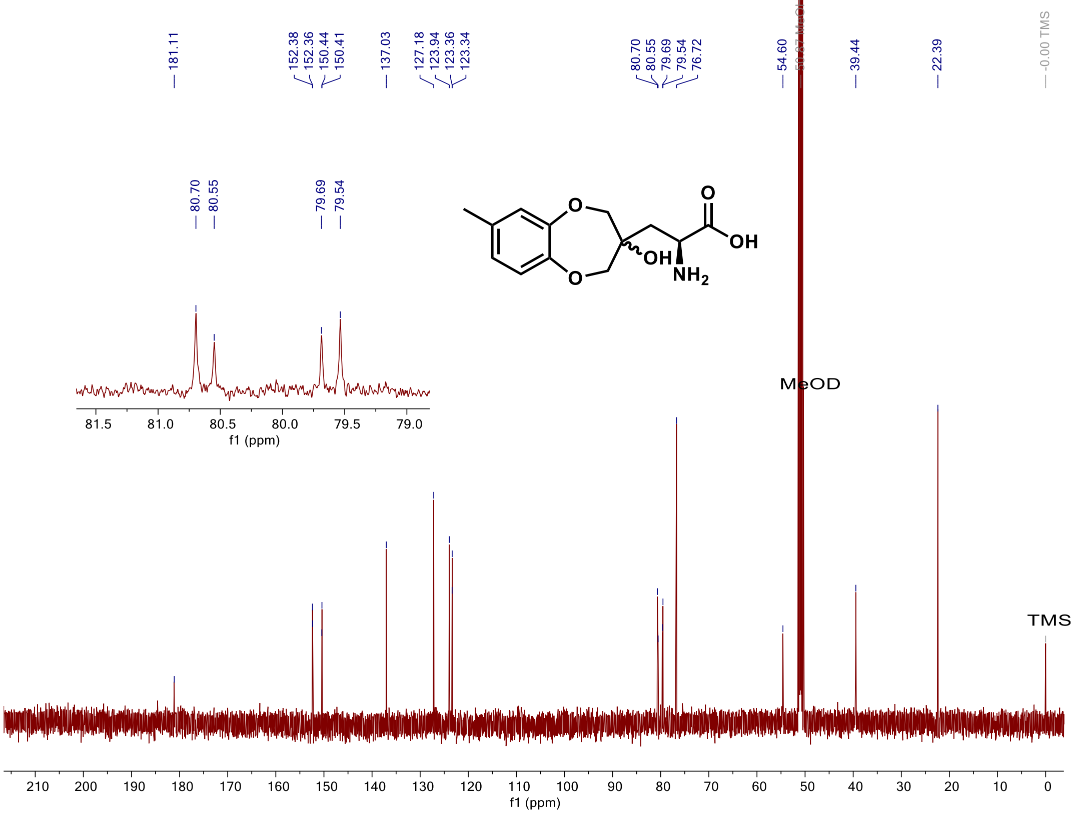

^{13}C NMR spectrum in D_2O:MeOH-d^4 at 126 MHz

2k

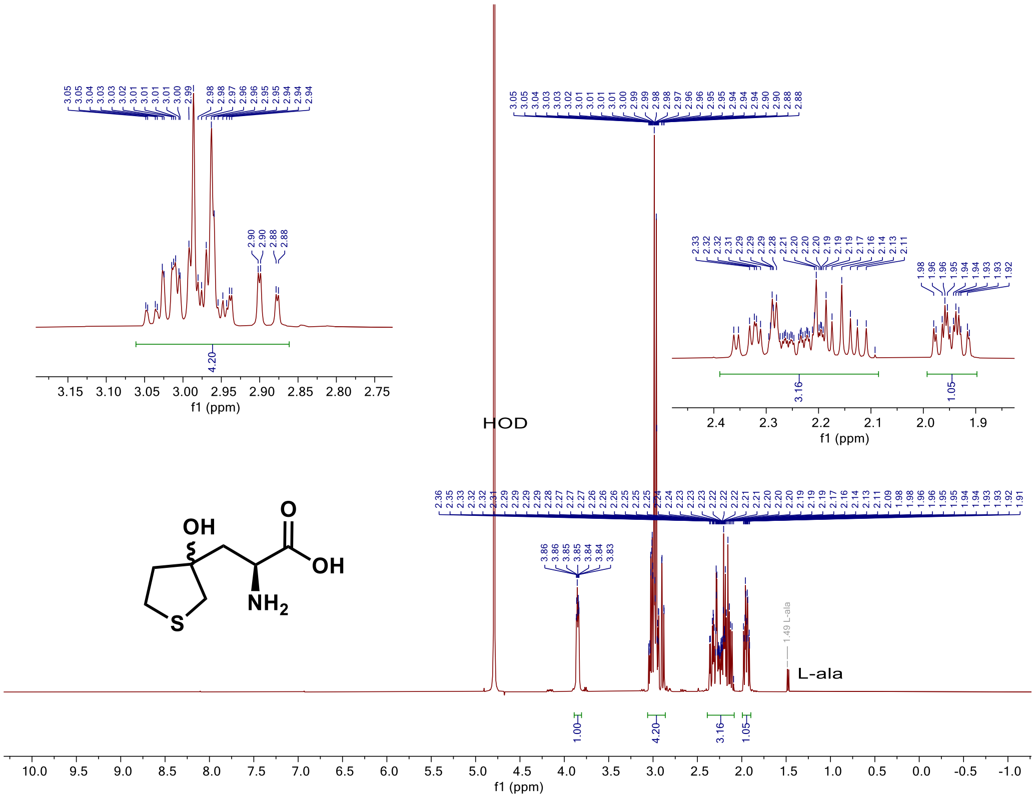

^{1}H NMR spectrum in D_2O at 500 MHz

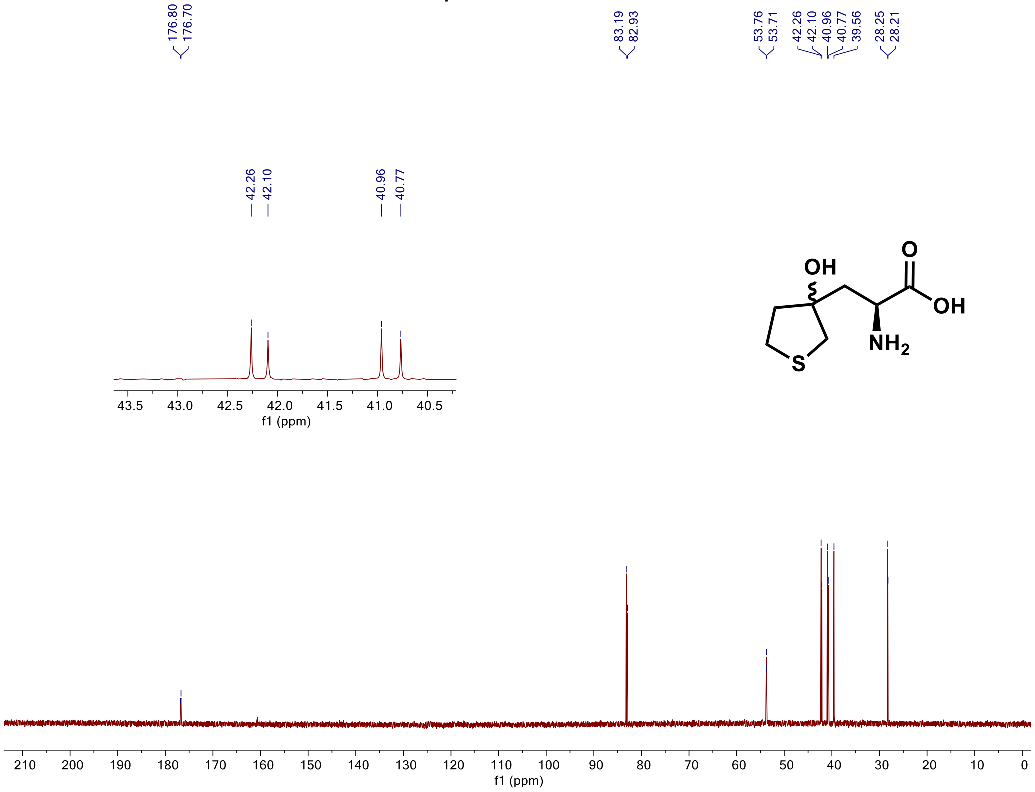

^{13}C NMR spectrum in D_2O at 126 MHz

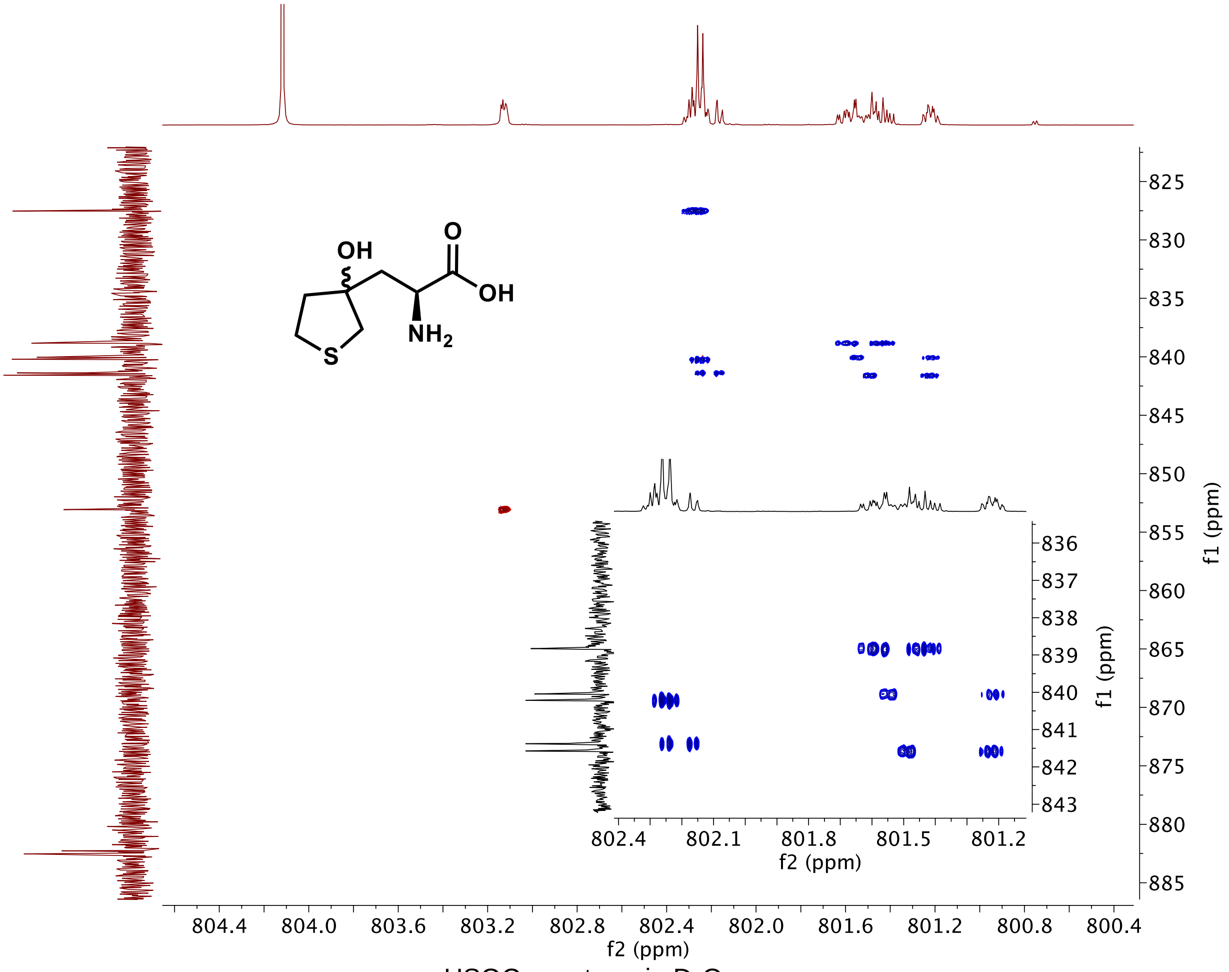

HSQC spectrum in D_2O

2l

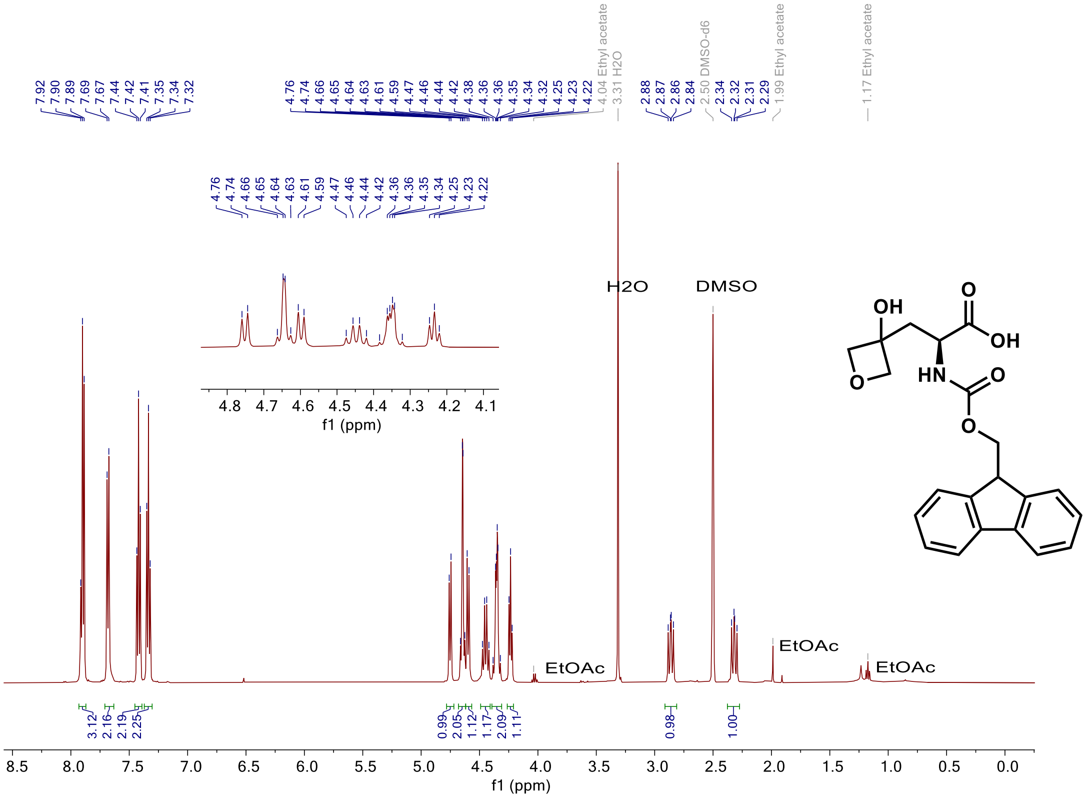

^{1}H NMR spectrum in DMSO-d^6 at 500 MHz

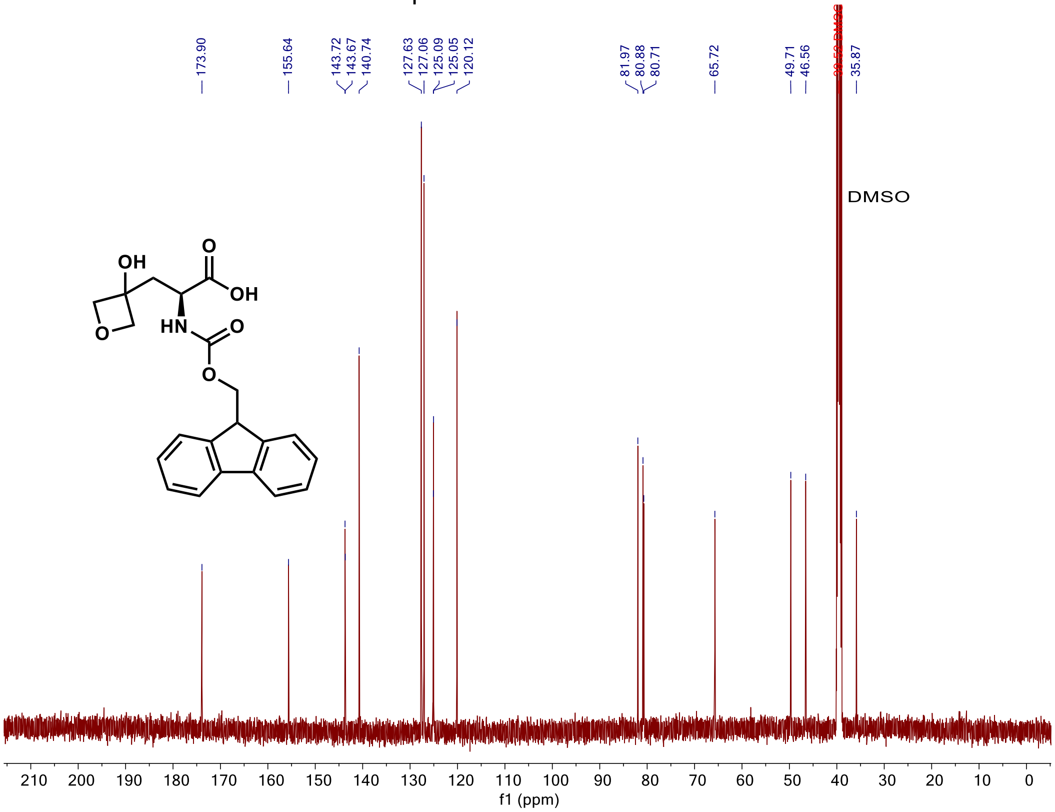

^{13}C NMR spectrum in DMSO-d^6 at 126 MHz

2m

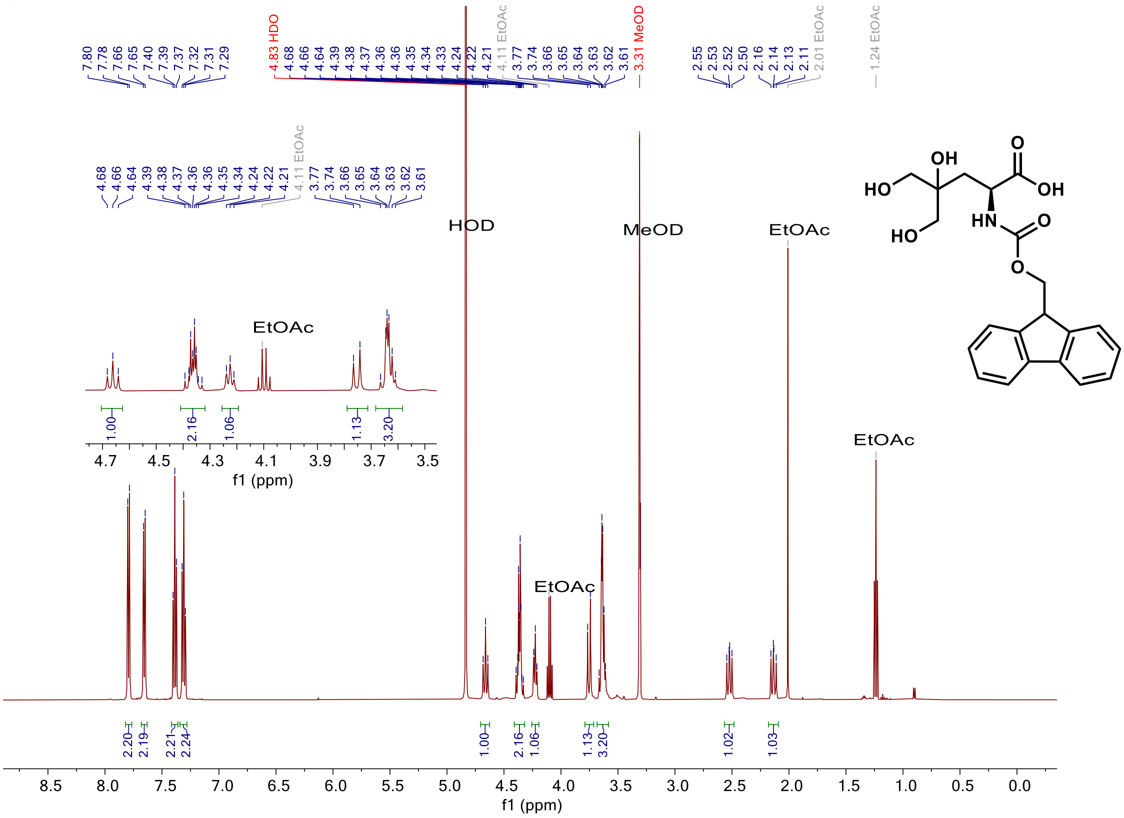

^{1}H NMR spectrum in MeOH-d^4 at 500 MHz

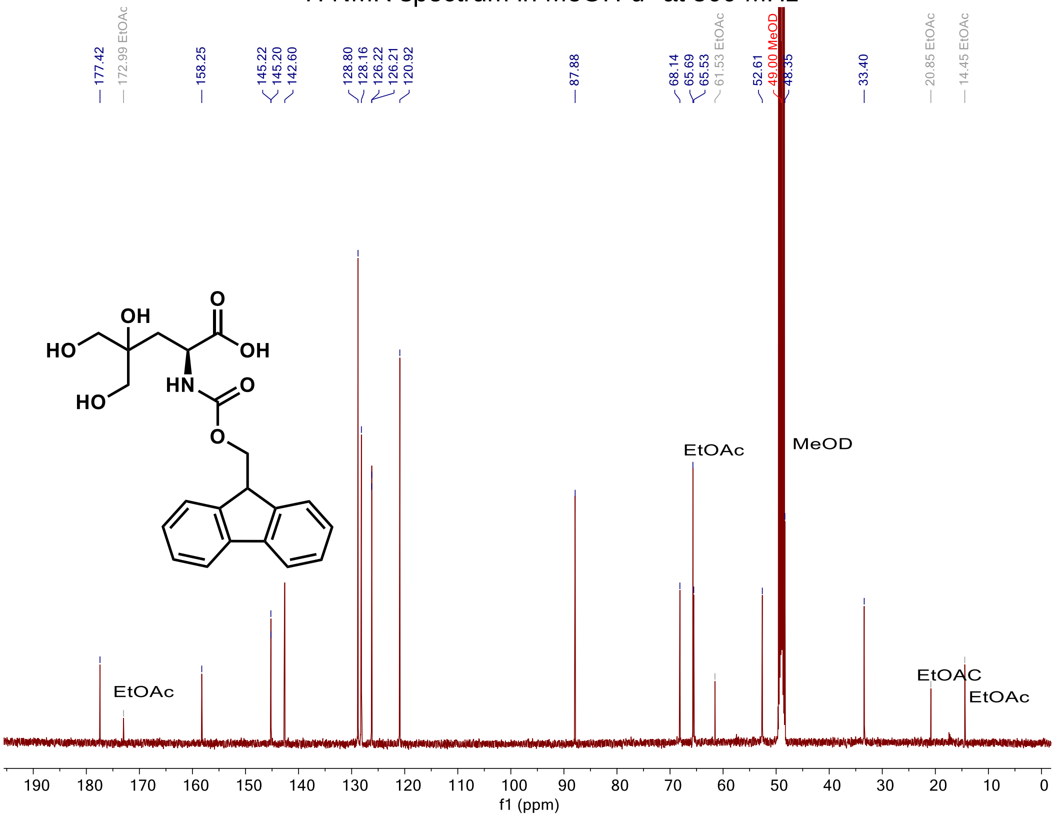

^{13}C NMR spectrum in MeOH-d^4 at 126 MHz

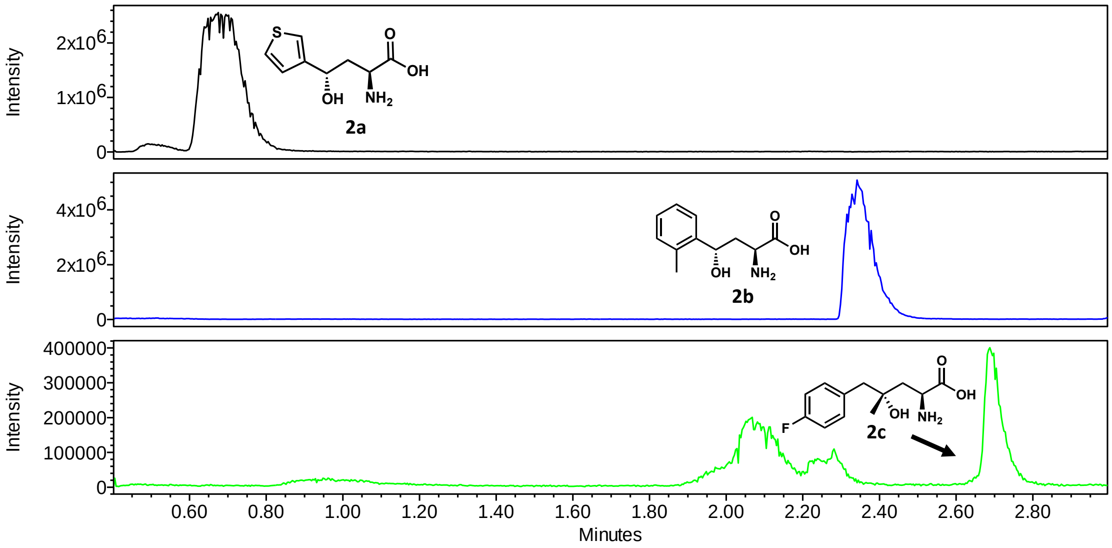

Figure S1. Product mass traces (**2a-c**), indicated by the structure, for the electrophile screening mixture. The aldehydes (**1a**, **1b**) are added in equal concentration (4.2 mM each) while ketone (**1c**) is added at 5-fold higher concentration (41.6 mM). **Conditions:** 50 mM L-asp, 50 mM total electrophiles, 50 µM PLP, 5% DMSO, 100 mM NaCl, 100 mM potassium phosphate pH 7.0, UstD$^{2.0}$ (whole cell), 37 °C, 200 rpm, 1 h reaction time.

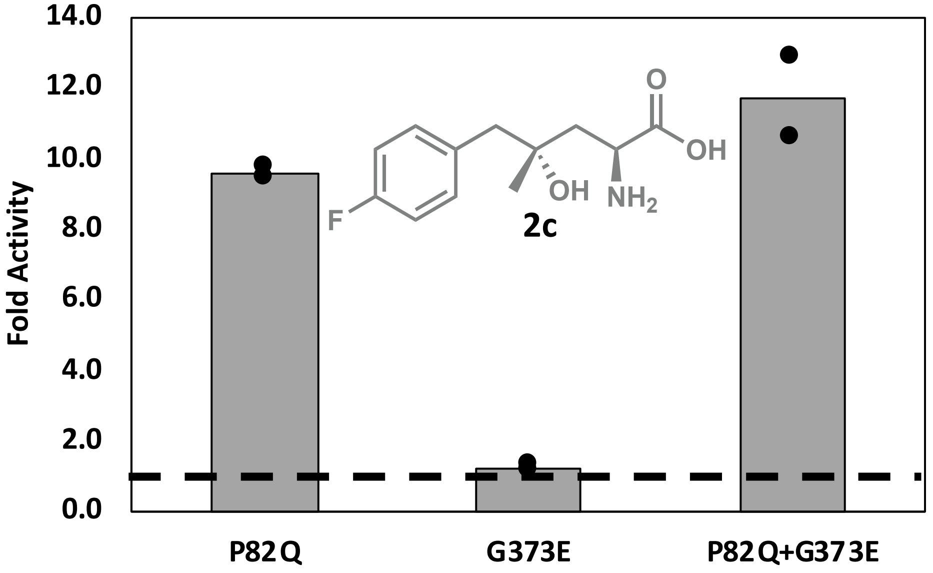

Figure S2. Single substrate reactions of P82Q and G373E SSM libraries, and QE double mutant under challenging conditions. Reactions were performed in duplicate, and activity was compared to UstD$^{2.0}$ reactions that were run simultaneously. The bars represent the average fold change of the replicates while the dots represent each individual measurement. The dotted line represents UstD$^{2.0}$ activity which is set to one. **Conditions:** 50 mM L-asp, 50 mM **1c,** 5 µM PLP, 5% DMSO, 100 mM NaCl, 100 mM potassium phosphate pH 7.0, 0.01 mol% cat (10,000 max turnovers), 37 °C, 16 h reaction time.

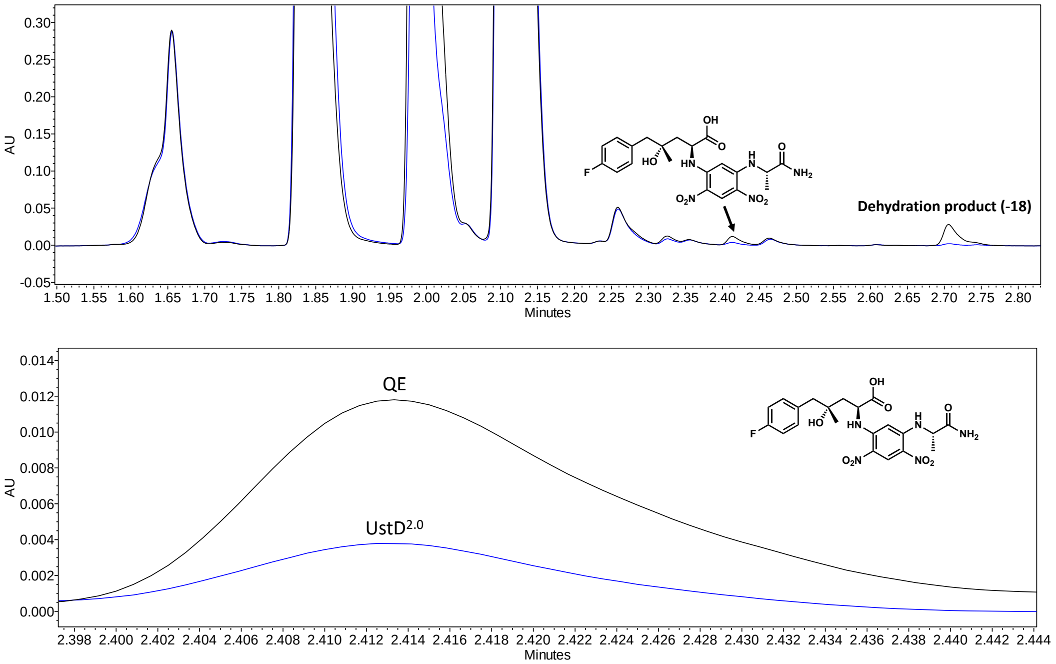

Figure S3. Overlap figure of UstD$^{2.0}$ and QE for product **2c**. The blue trace is UstD$^{2.0}$ activity while the black trace is QE activity.

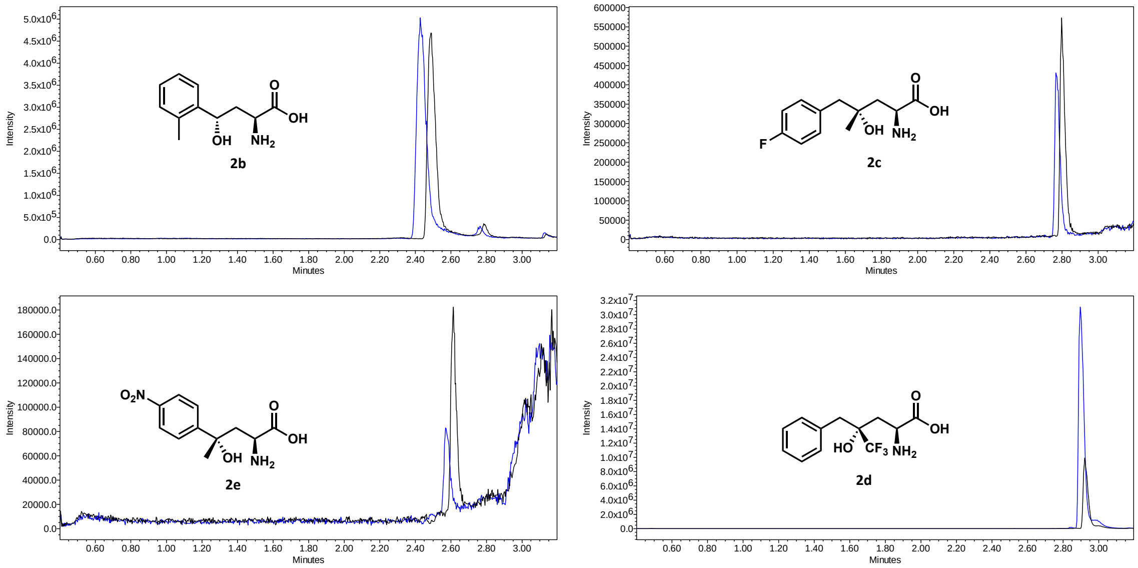

Figure S4. Overlayed product mass traces, indicated by the structure, for the different electrophile mixtures. In blue, substrates are added in equal amounts (12.5 mM each). In black, the substrates are added in differing amounts according to their electrophilicity (5 mM **1b**, 10 mM **1c**, 2.5 mM **1d**, 32.5 mM **1e**). **Conditions:** 50 mM L-asp, 50 mM total electrophiles, 5 µM PLP, 5% DMSO, 100 mM NaCl, 100 mM potassium phosphate pH 7.0, QE (0.01 mol% catalyst, 10,000 Max TON), 37 °C, 1 h reaction time.

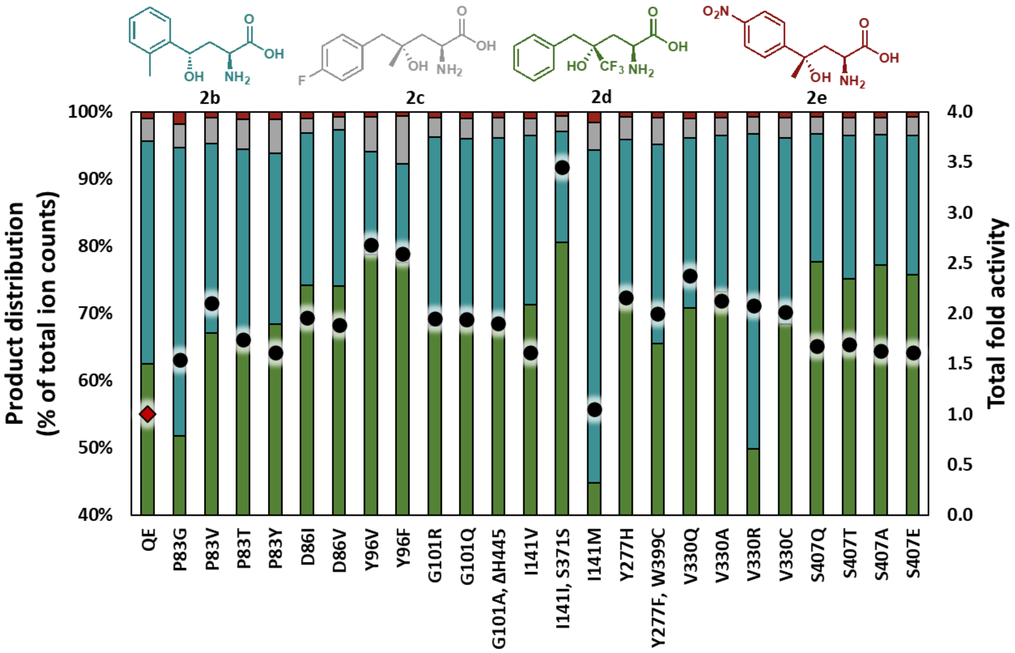

Figure S5. SSM results for libraries using QE as parent. All sequenced variants are displayed on the x-axis. The stacked bars depict the product distribution with each color representing the corresponding chiral alcohol product. The black dots show the total fold activity for all products. The total fold activity for QE is represented by the red diamond and is set to one. The QE product distribution is the average QE product distribution across all library plates. **Conditions:** 50 mM L-asp, 5 mM **1b**, 10 mM **1c**, 2.5 mM **1d**, 32.5 mM **1e**, 50 µM PLP, 5% DMSO, 100 mM NaCl, 100 mM potassium phosphate pH 7.0, *E. coli* whole cells over-expressing QE variants, 37 °C, 200 rpm, 1 h reaction time.

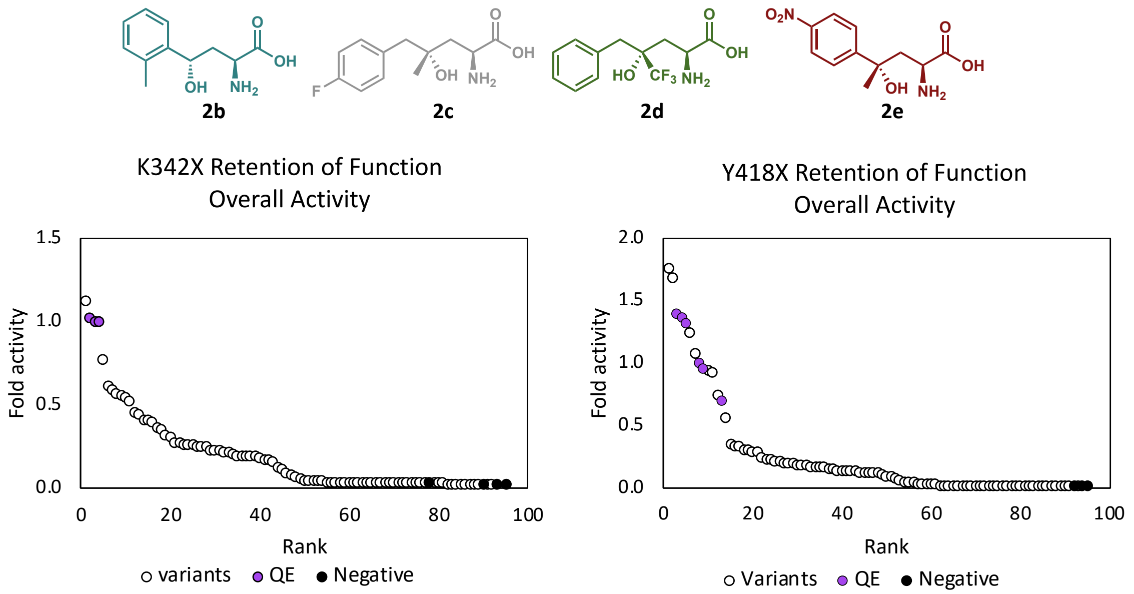

Figure S6. Retention of function curves for total activity at sites K342 and Y418 for their respective site saturation libraries. Neither library shows any activated variants (white dots) compared to the QE parent enzyme (purple dots). Negative controls and the sterile well are shown in black. **Conditions:** 50 mM L-asp, 5 mM **1b**, 10 mM **1c**, 2.5 mM **1d**, 32.5 mM **1e**, 50 µM PLP, 5% DMSO, 100 mM NaCl, 100 mM potassium phosphate pH 7.0, *E. coli* whole cells over-expressing QE variants, 37 °C, 200 rpm, 1 h reaction time.

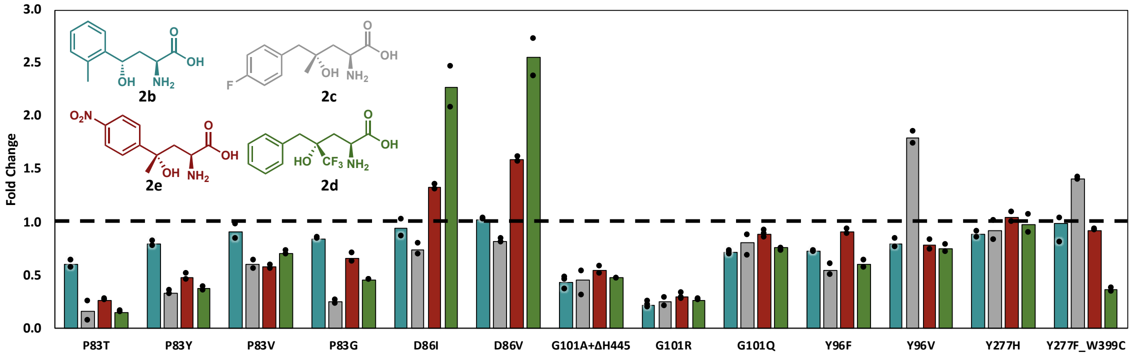

Figure S7. Subset of variants from SSM libraries re-screened in lysate against single substrates. Reactions were performed in at least duplicate, and activity was compared to QE reactions that were run simultaneously to determine fold change (y-axis). The bars represent the average fold change of the replicates while the dots represent each individual measurement. The dotted line represents QE activity which is set to one. The color of the bars corresponds to the product structure displayed on the chart. **Conditions:** 50 mM L-asp, 50 mM electrophile, 50 µM PLP, 5% DMSO, 100 mM NaCl, 100 mM potassium phosphate pH 7.0, 40-50 mg/mL lysate, 37 °C, 16 h reaction time, QE parent enzyme.

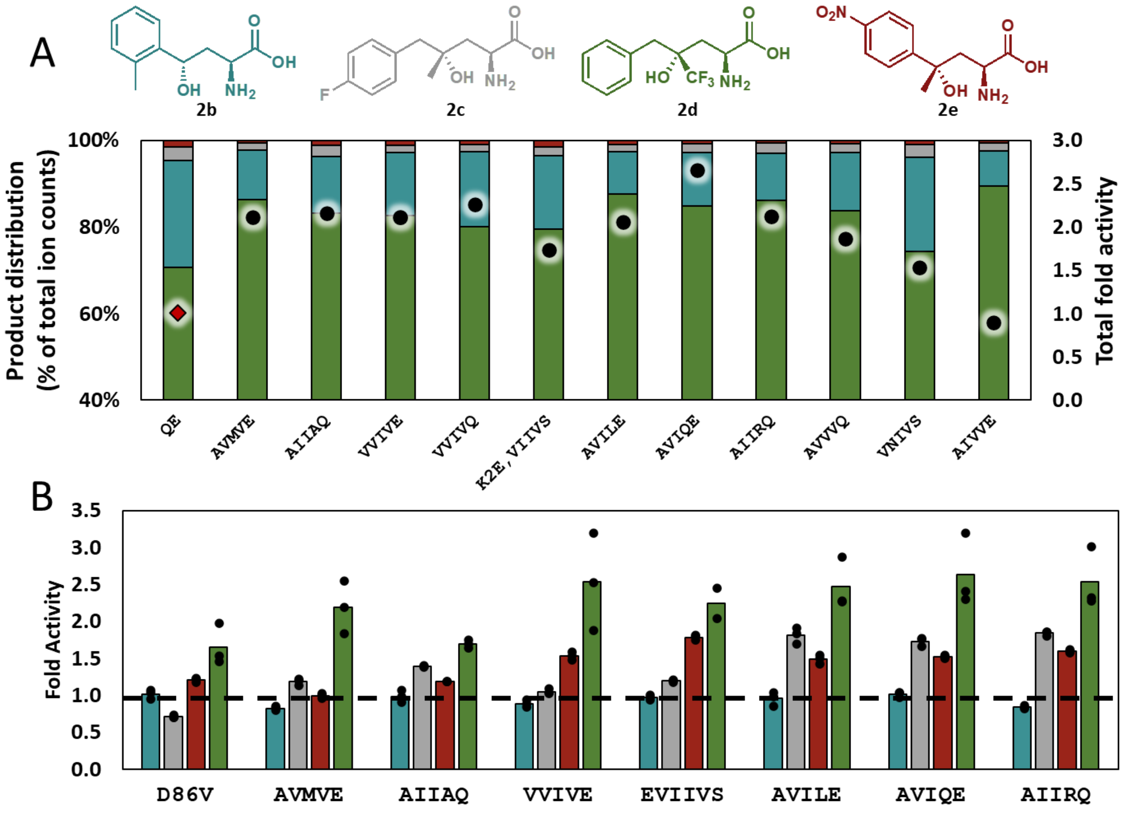

Figure S8. A. All sequenced variants are displayed on the x-axis. The stacked bars depict the product distribution with each color representing the corresponding chiral alcohol product. The black dots show the total fold activity for all products. The total fold activity for QE is represented by the red diamond and is set to one. The QE product distribution is the average QE product distribution across all library plates. **Conditions:** 50 mM L-asp, 5 mM **1b**, 10 mM **1c**, 2.5 mM **1d**, 32.5 mM **1e**, 50 µM PLP, 5% DMSO, 100 mM NaCl, 100 mM potassium phosphate pH 7.0, *E. coli* whole cells over-expressing QE variants, 37 °C, 200 rpm, 8 h reaction time. **B.** Subset of variants from recombination libraries re-screened against single substrates with purified protein. Reactions were performed in triplicate, and activity was compared to QE reactions that were run simultaneously to determine fold change (y-axis). The bars represent the average fold change of the replicates while the dots represent each individual measurement. The dotted line represents QE activity which is set to one. The color of the bars corresponds to the product structure displayed on the chart. **Conditions:** 50 mM L-asp, 50 mM electrophile, 5 µM PLP, 5% DMSO, 100 mM NaCl, 100 mM potassium phosphate pH 7.0, 0.01 mol% catalyst (10,000 Max turnovers), 37 °C, 16 h reaction time.

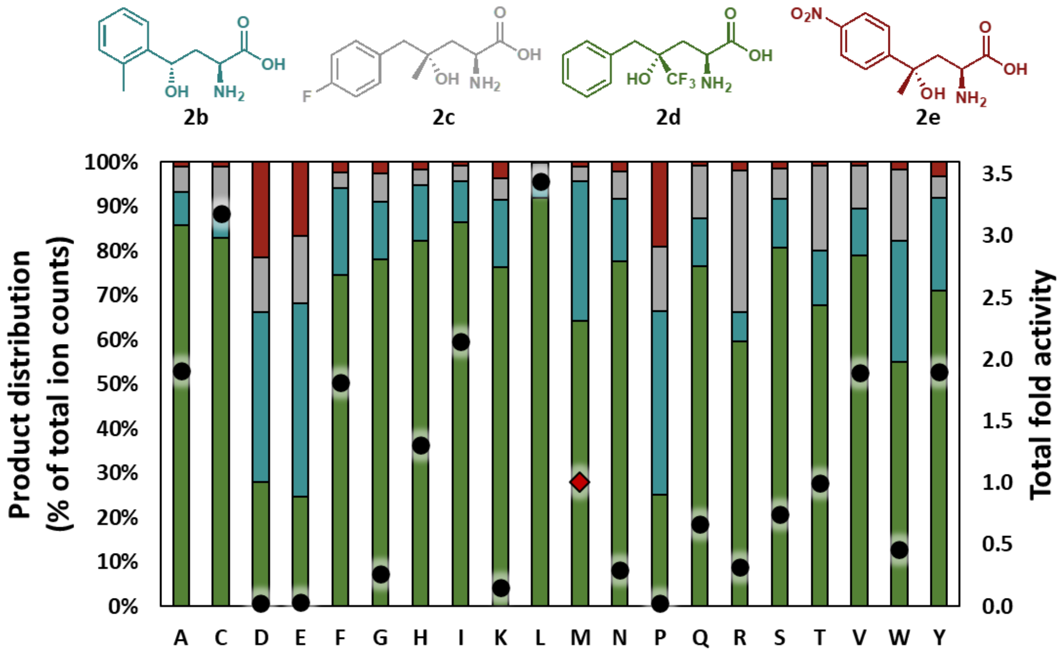

Figure S9. M299X SSM library data. All sequenced variants are displayed on the x-axis. The stacked bars depict the product distribution with each color representing the corresponding chiral alcohol product. The black dots show the total fold activity for all products. The total fold activity for QE is represented by the red diamond and is set to one. **Conditions:** 50 mM L-asp, 5 mM **1b**, 10 mM **1c**, 2.5 mM **1d**, 32.5 mM **1e**, 50 µM PLP, 5% DMSO, 100 mM NaCl, 100 mM potassium phosphate pH 7.0, *E. coli* whole cells over-expressing QE variants, 37 °C, 200 rpm, 1 h reaction time.

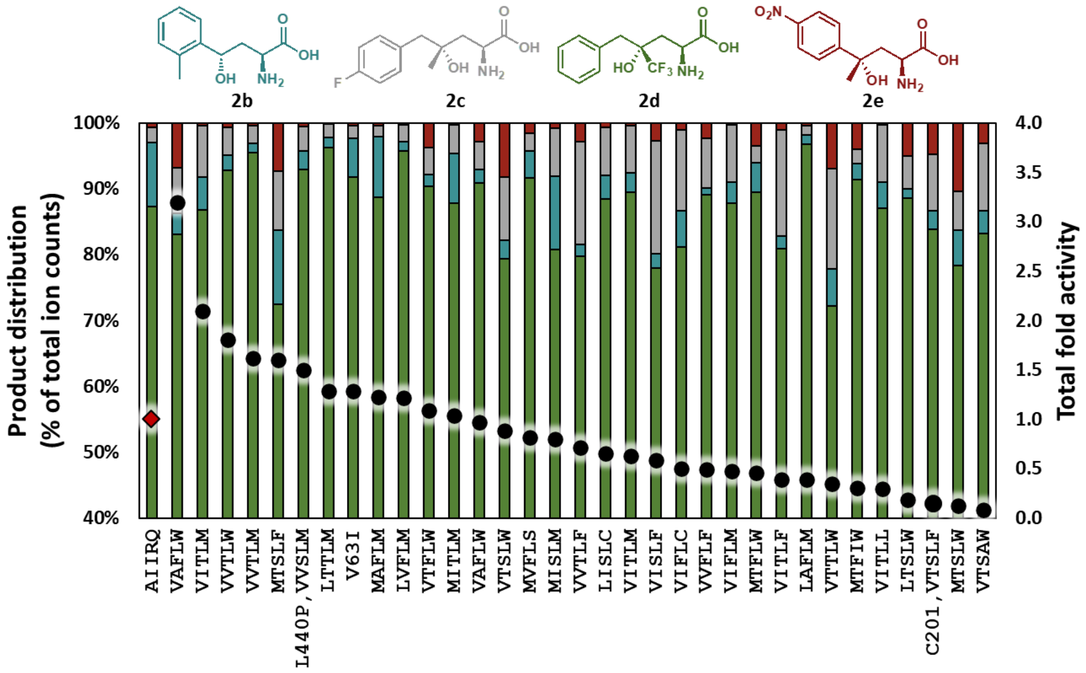

Figure S10. Library data of all sequenced active site recombination variants. All sequenced variants are displayed on the x-axis. The stacked bars depict the product distribution with each color representing the corresponding chiral alcohol product. The distribution is shown starting at 40% to make visualization of the promiscuity shifts clearer for **2c** and **2e**. The black dots show the total fold activity for all products. The total fold activity for AIIRQ is represented by the red diamond and is set to one. The AIIRQ product distribution is the average product distribution across all library plates. **Conditions:** 50 mM L-asp, 5 mM **1b**, 10 mM **1c**, 2.5 mM **1d**, 32.5 mM **1e**, 50 µM PLP, 5% DMSO, 100 mM NaCl, 100 mM potassium phosphate pH 7.0, *E. coli* whole cells over-expressing QE variants, 37 °C, 200 rpm, 6 h reaction time.

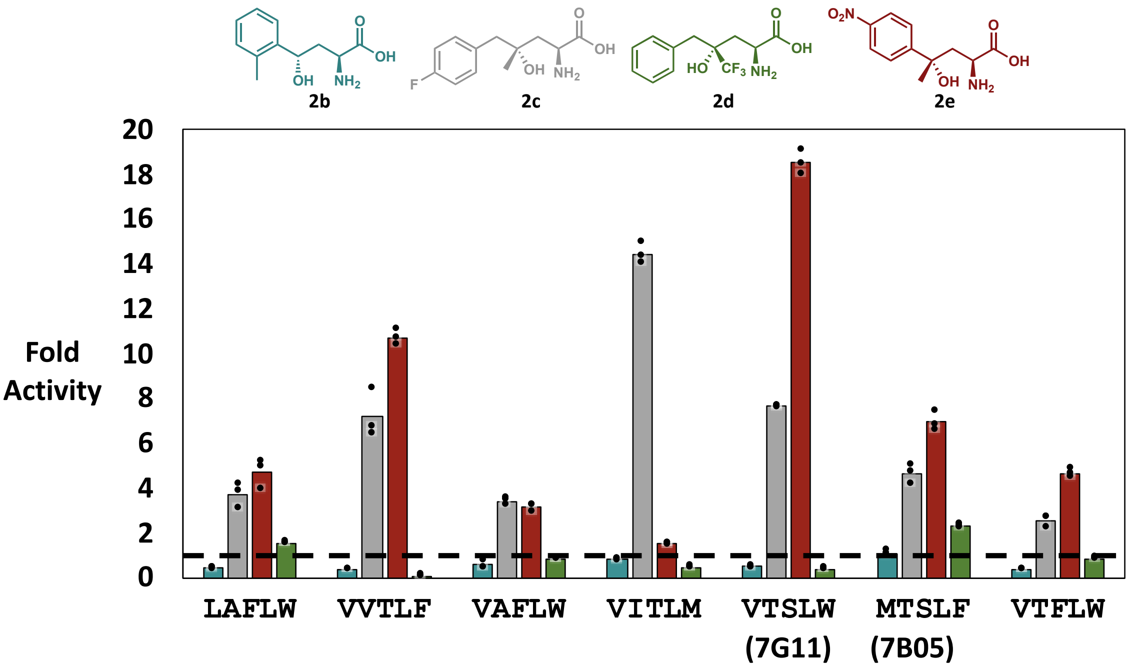

Figure S11. Subset of variants from active-site recombination libraries re-screened against single substrates with purified protein. Reactions were performed in triplicate, and activity was compared to AIIRQ reactions that were run simultaneously to determine fold change (y-axis). The bars represent the average fold activity of the replicates while the dots represent each individual measurement. The dotted line represents AIIRQ activity which is set to one. The color of the bars corresponds to the product structure displayed above the chart. **Conditions:** 50 mM L-asp, 50 mM electrophile, 5 µM PLP, 5% DMSO, 100 mM NaCl, 100 mM potassium phosphate pH 7.0, 0.01 mol% catalyst (10,000 Max turnovers), 37 °C, 16 h reaction time.

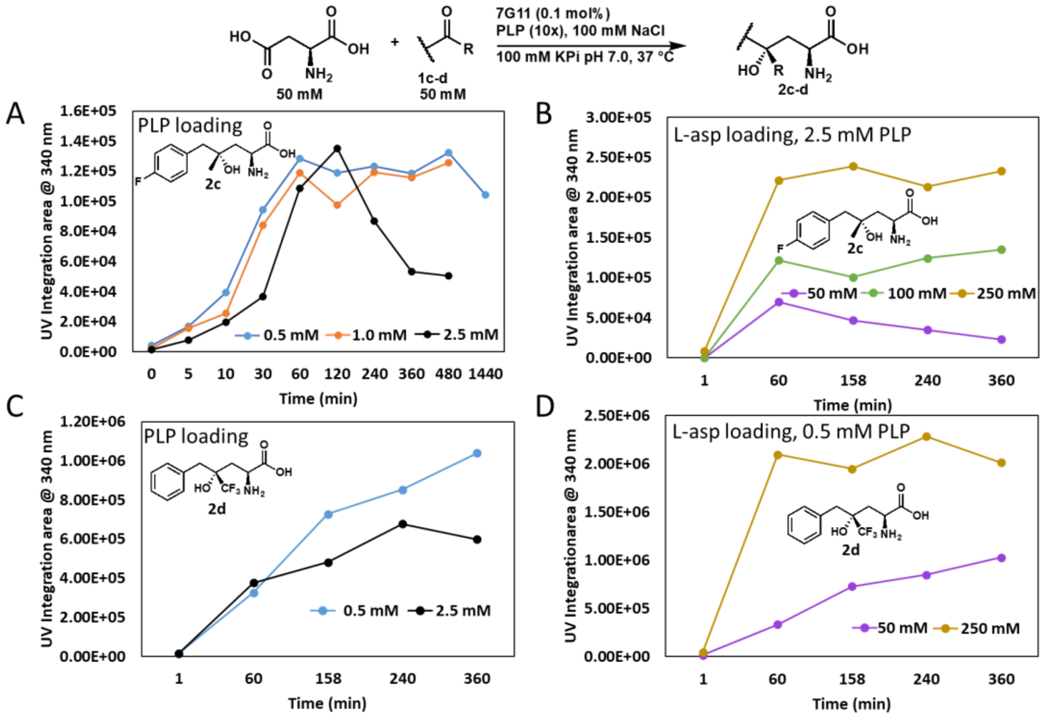

Figure S12. Reaction condition optimization of 7G11. Initial reaction conditions are depicted in the top scheme. Conditions were optimized sequentially: **A.** 2.5 mM (50x) PLP relative to catalyst for **1c**, **B.** 250 mM (5x) L-aspartate for **1c**, **C.** 0.5 mM (10x) PLP relative to catalyst for **1d** **D.** 250 mM (5x) L-aspartate for **1d**.

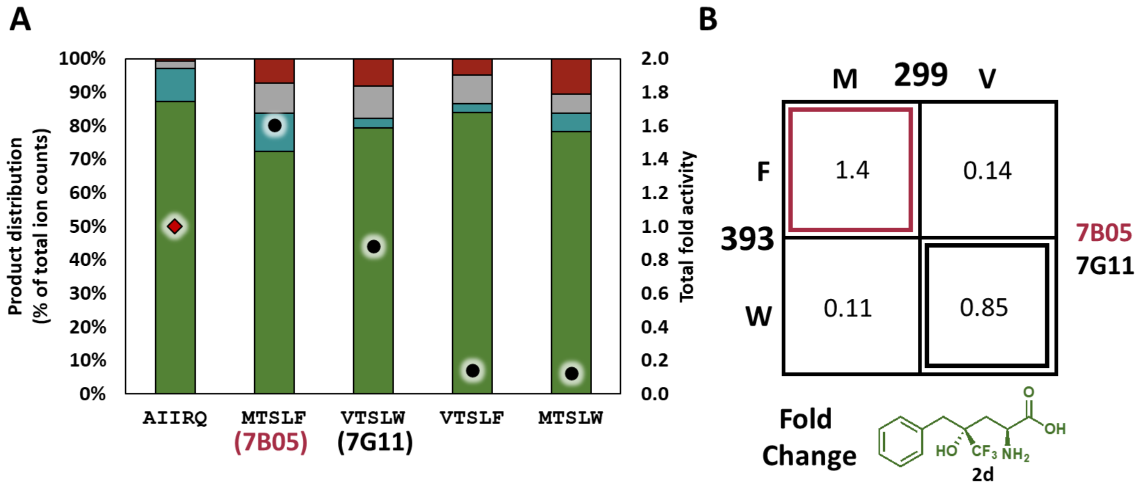

Figure S13. Active site variants that are a single stepwise mutational walk between the 7G11 and 7B05. A. Library screening data comparing the two final variants against variants that are intermediate mutational walks. **B.** Mutations at each residue are displayed outside the square. The number inside each box represents the fold change of **2d.** The colored boxes indicate which variants are 7B05 (pink) and 7G11 (black).

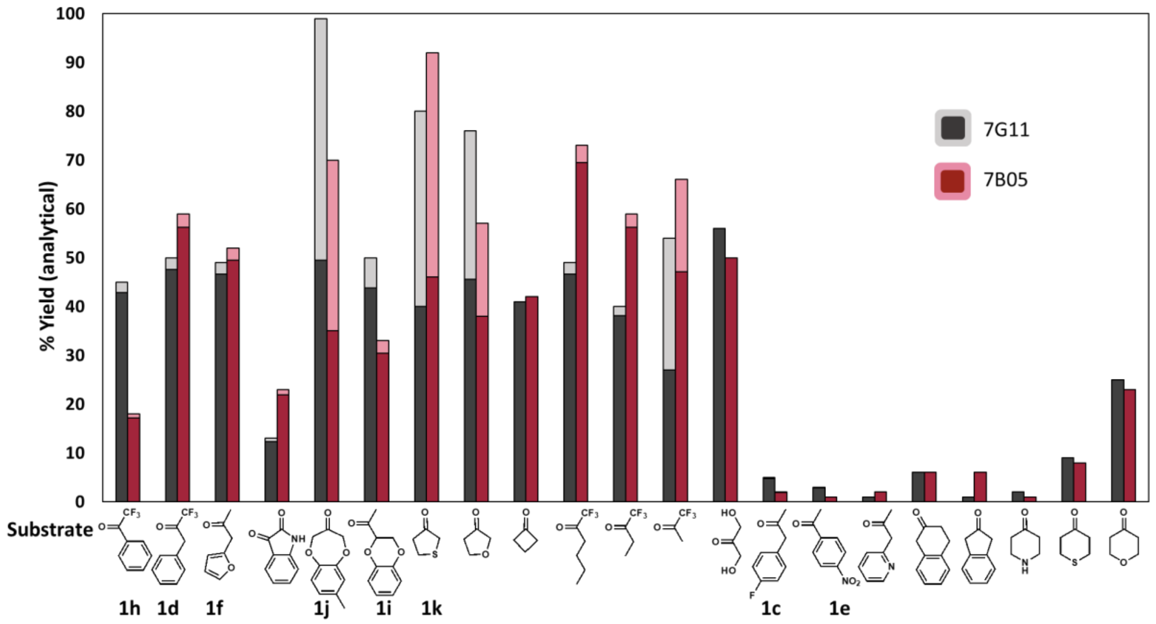

Figure S14. Analytical evaluation of substrate scope. All yields were quantified by Marfey's derivatization against a standard curve as described in the SI for the lineage analysis. The lighter colored bars represent the amount of syn-diastereomer formed with the assumption the anti-diastereomer is the major isomer. Each entry represents only a single reaction. The black bars are reactions with 7G11 and the pink bars are reactions with 7B05. **Conditions:** 250 mM L-asp, 50 mM electrophile, 10x or 50x PLP compared to enzyme, 5% DMSO, 100 mM NaCl, 100 mM potassium phosphate pH 7.0, 7B05 or 7G11 (0.1 mol% cat, 1000 Max TON, 37 °C, 4 h reaction time.

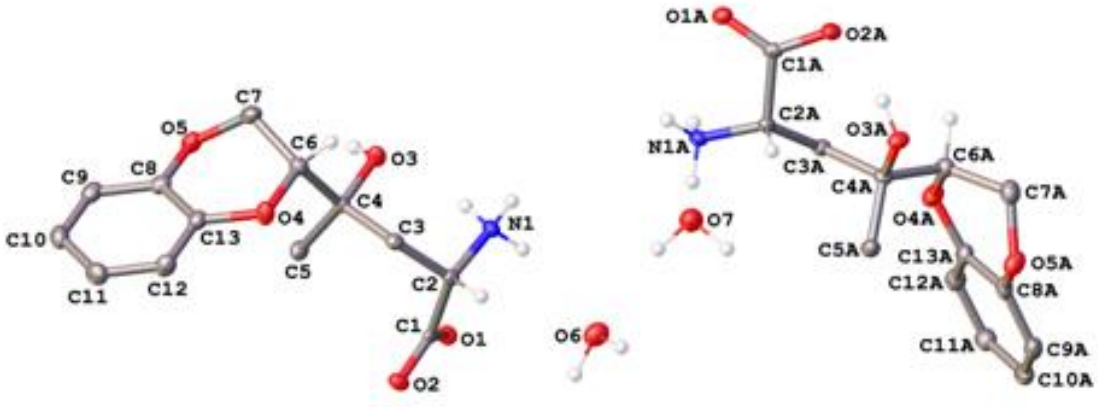

Figure S15. A molecular drawing of the asymmetric unit in **2i** shown with 50% probability ellipsoids. All H atoms (except those bound to N/O atoms or chiral centers) are omitted. The absolute configuration of the chiral atoms is C2–S, C4–S, C6–R, C2A–S, C4A–S, and C6A–R.

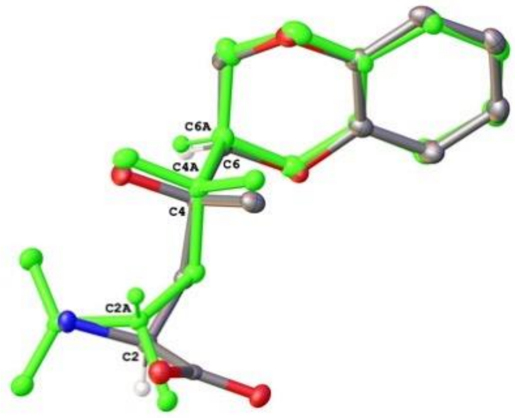

Figure S16. A molecular drawing overlaying the two symmetry independent molecules in **2i** shown with 50% probability ellipsoids. All H atoms (except those bound to chiral centers) and solvent molecules are omitted. The second symmetry independent molecule is shown in green. The absolute configuration of the chiral atoms is C2–S, C4–S, C6–R, C2A–S, C4A–S, and C6A–R.

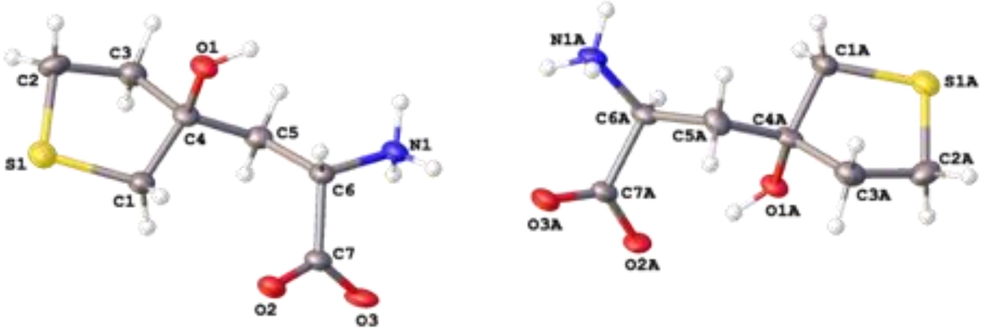

Figure S17. A molecular drawing of **2k** shown with 50% probability ellipsoids. The two molecules are diastereomers.

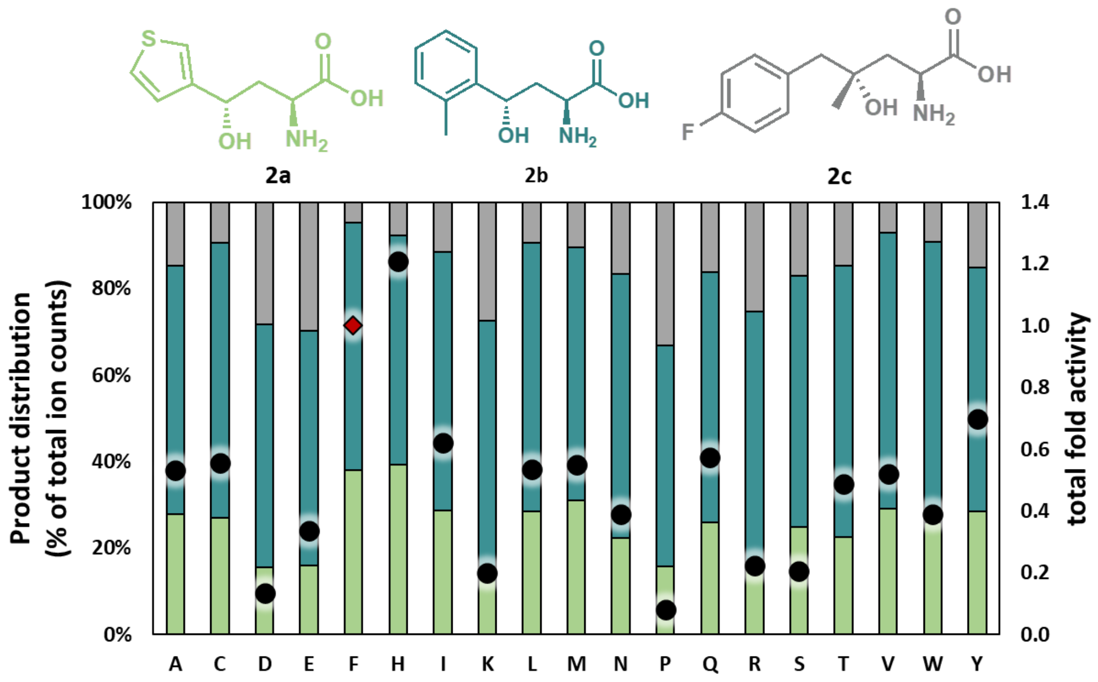

Figure S18. F75X SSM library data. All sequenced variants are displayed on the x-axis. The stacked bars depict the product distribution with each color representing the corresponding chiral alcohol product. The black dots show the total fold activity for all products. The total fold activity for UstD$^{2.0}$ is represented by the red diamond and is set to one. **Conditions:** 50 mM L-asp, 4.2 mM **1a**, 4.2 mM **1b**, 41.6 mM **1c**, 50 µM PLP, 5% DMSO, 100 mM NaCl, 100 mM potassium phosphate pH 7.0, *E. coli* whole cells over-expressing UstD$^{2.0}$ variants, 37 °C, 200 rpm, 1 h reaction time.

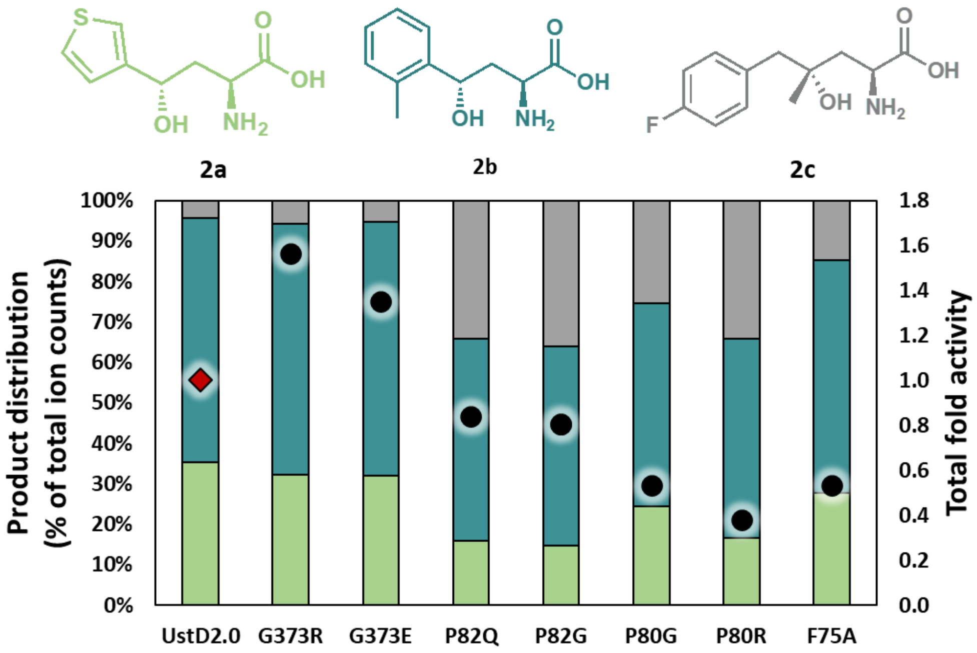

Figure S19. Promiscuity-shifting variants of interest from site saturation mutagenesis at sites F75, P80, P82, G373. Total fold activity for all products is represented by the dots. The total fold activity for UstD$^{2.0}$ is represented by the red diamond and is set to one. Product distribution is represented by the bars. The mutations found in each variant are displayed on the x-axis. **Conditions:** 50 mM L-asp, 4.2 mM **1a**, 4.2 mM **1b**, 41.6 mM **1c**, 50 µM PLP, 5% DMSO, 100 mM NaCl, 100 mM potassium phosphate pH 7.0, *E. coli* whole cells over-expressing UstD$^{2.0}$ variants, 37 °C, 200 rpm, 1 h reaction time.

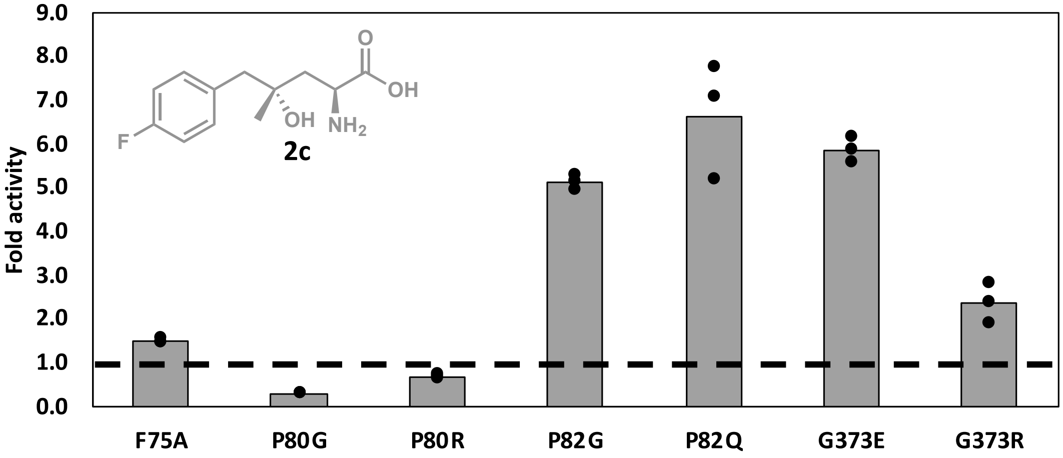

Figure S20. Single substrate reactions for F75X, P80X, P82X, and G373X at early timepoints. Reactions were performed in at least duplicate, and activity was compared to UstD$^{2.0}$ reactions that were run simultaneously. The bars represent the average fold change of the replicates while the dots represent each individual measurement. The dotted line represents UstD$^{2.0}$ activity which is set to one. **Conditions:** 50 mM L-asp, 50 mM **1c**, 50 µM PLP, 5% DMSO, 100 mM NaCl, 100 mM potassium phosphate pH 7.0, 20 mg/mL *E. coli* whole cells over-expressing UstD$^{2.0}$ variants, 37 °C, 200 rpm, 1 h reaction time.

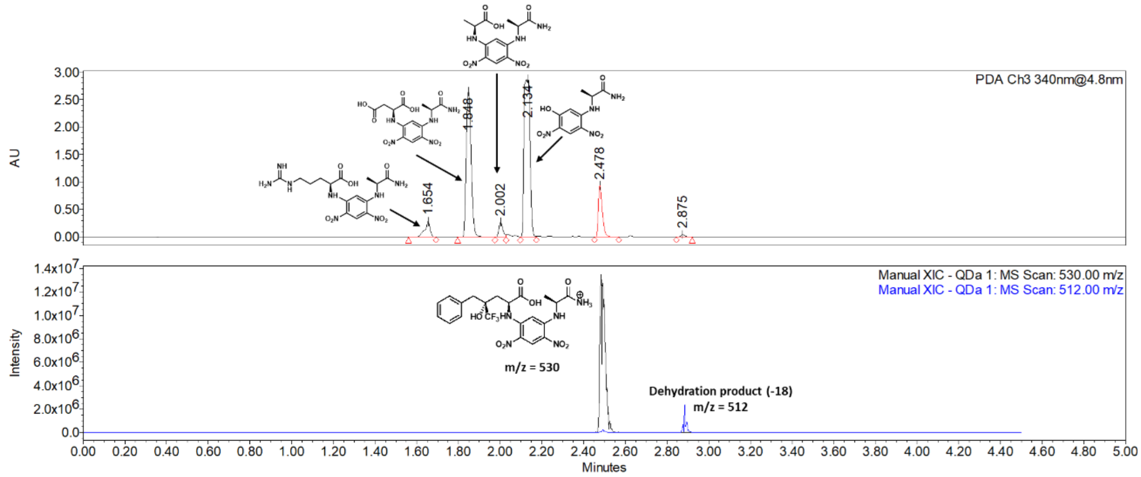

Figure S21. Representative UPLC trace for **2d** following Marfey's derivatization. The top trace displays the absorbance at 340 nm while the bottom trace displays the relevant product masses and structures. The common side products of the derivatization (L-ala, L-asp, hydrolyzed Marfey's reagent) and the internal standard (L -arg) are displayed on the trace. These additional peaks are present for Marfey's reactions performed on crude reaction mixtures. **Note:** Marfey's reactions are quenched with 60 mM HCl which causes dehydration of the amino acid products resulting in two product peaks with masses differing by 18 mass units. The molecular ion is shown in black.

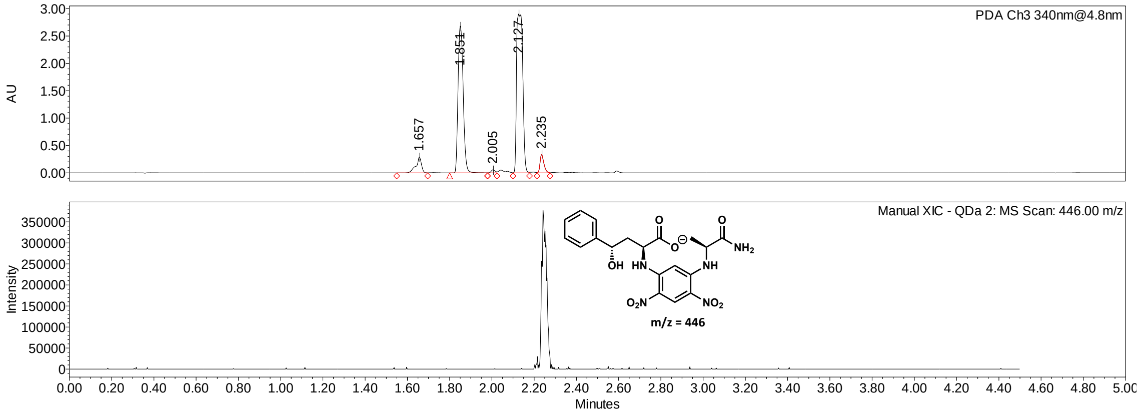

Figure S22. Representative UPLC trace for **2g** following Marfey's derivatization. The top trace displays the absorbance at 340 nm while the bottom trace displays the relevant product masses and structures. The molecular ion is shown in black.

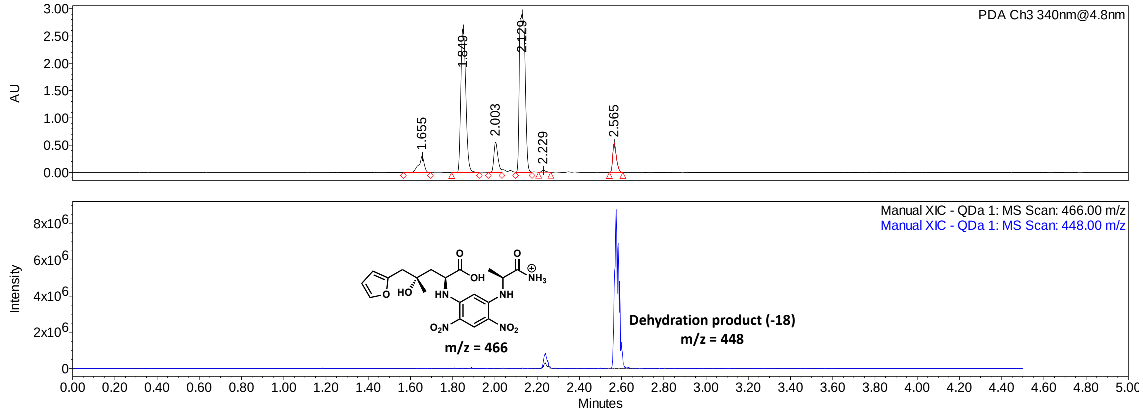

Figure S23. Representative UPLC trace for **2f** following Marfey's derivatization. The top trace displays the absorbance at 340 nm while the bottom trace displays the relevant product masses and structures. **Note:** Marfey's reactions are quenched with 60 mM HCl which causes lactonization of the amino acid products resulting in two product peaks with masses differing by 18 mass units. The molecular ion is shown in black.

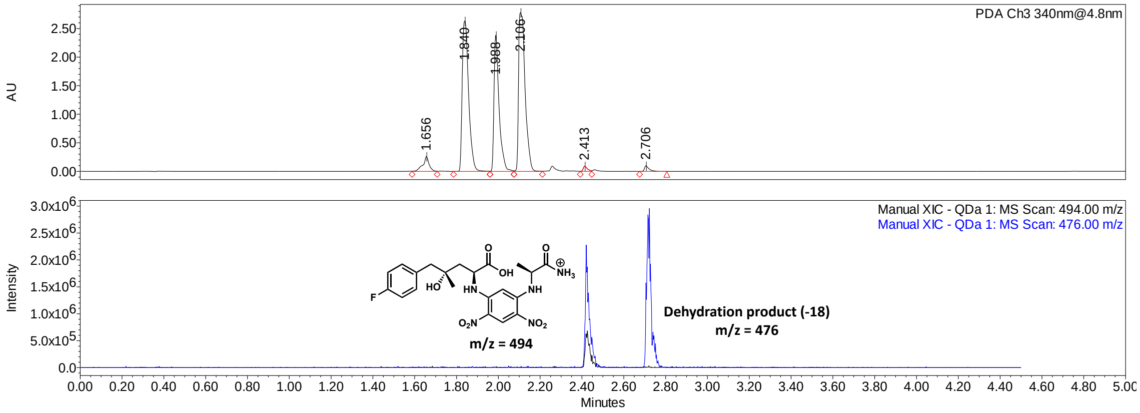

Figure S24. Representative UPLC trace for **2c** following Marfey's derivatization. The top trace displays the absorbance at 340 nm while the bottom trace displays the relevant product masses and structures. **Note:** Marfey's reactions are quenched with 60 mM HCl which causes lactonization of the amino acid products resulting in two product peaks with masses differing by 18 mass units. The molecular ion is shown in black.

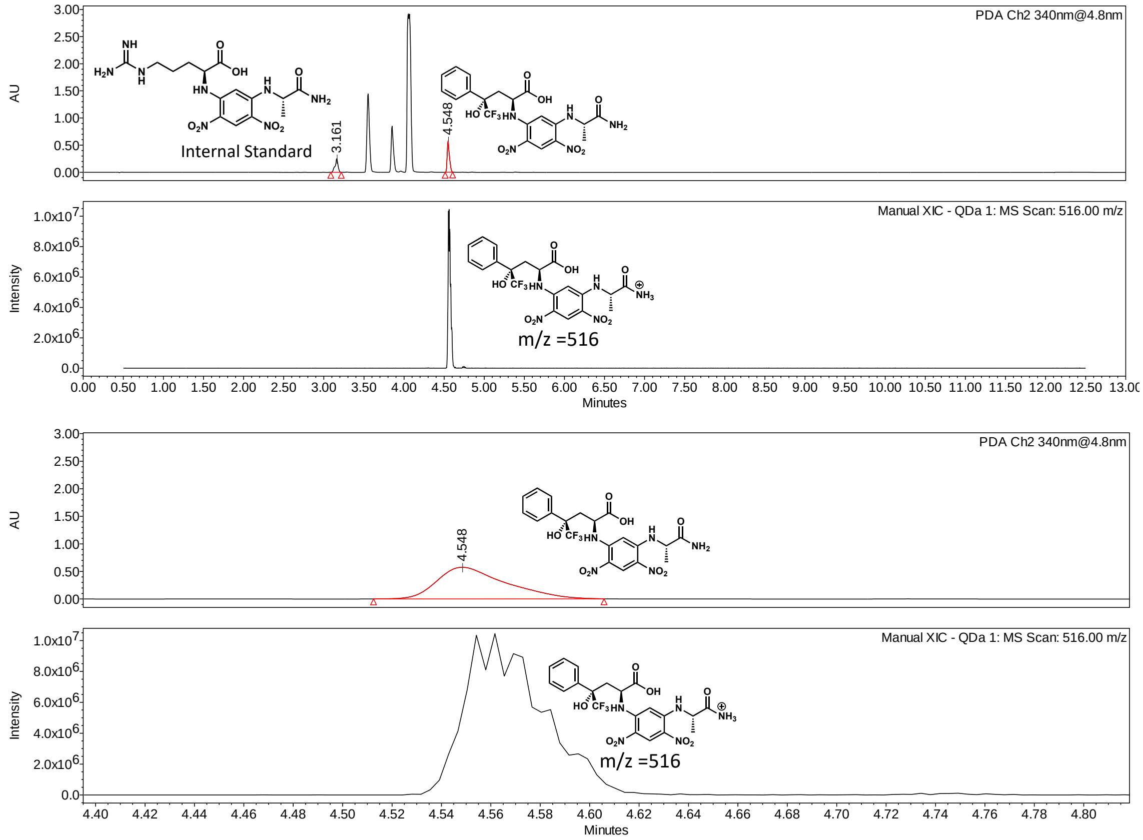

Figure S25. UPLC-MS trace of Marfey's derivatization for **2h**. The bottom pane shows an enlarged view of the **2h** peak. Conditions: 50 mM 1h, 250 mM L-asp, PLP (10x to catalyst), 7G11 (0.1 mol% cat, 1000 Max TON), 5% MeOH, 100 mM potassium phosphate buffer, pH 7.0, 100 mM NaCl, 37 °C, 4 h.

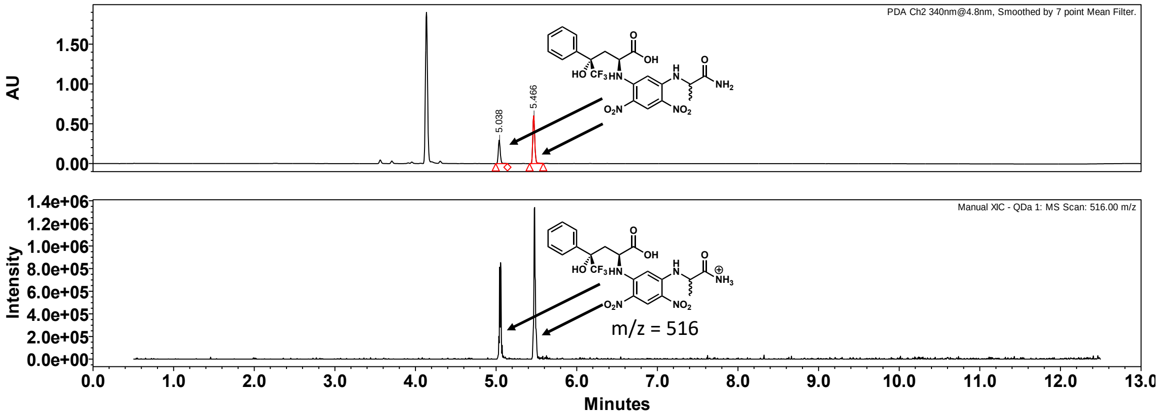

Figure S26. UPLC-MS trace of racemic Marfey's derivatization for **2h.**

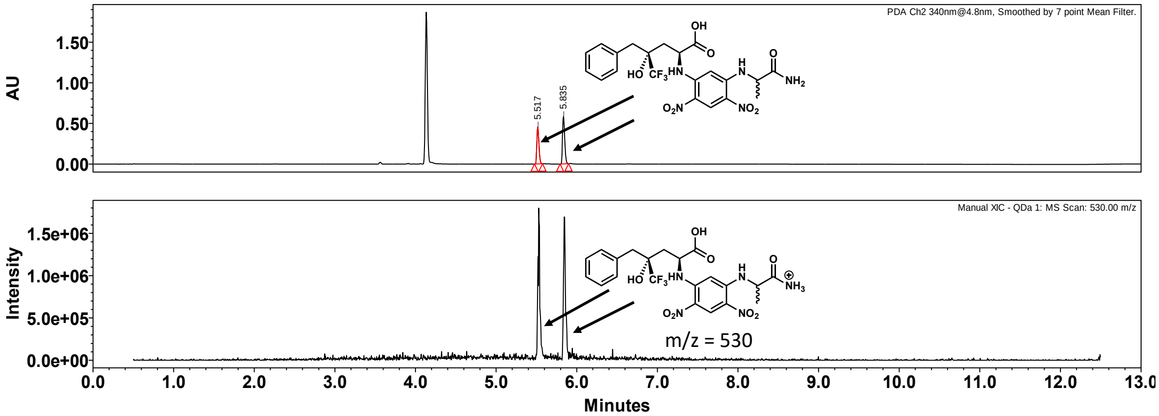

Figure S27. UPLC-MS trace of racemic Marfey's derivatization for **2d.**

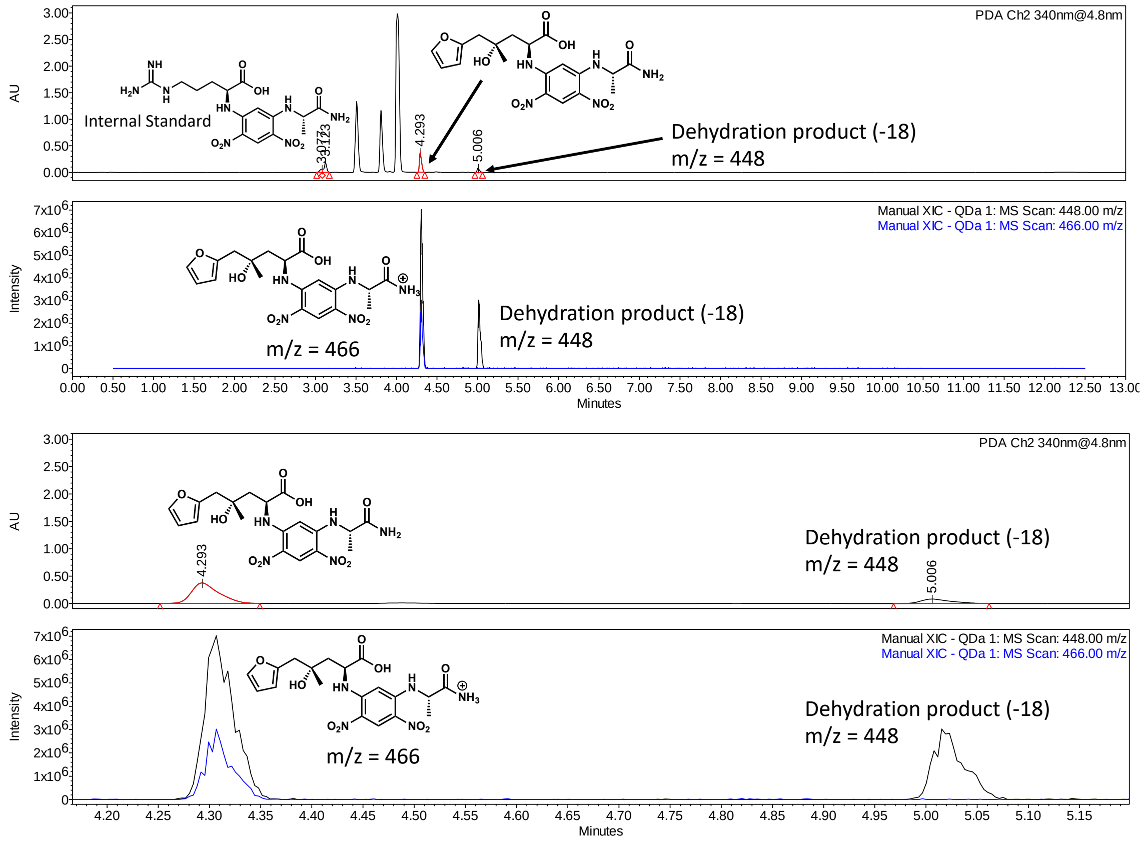

Figure S28. UPLC-MS trace of Marfey's derivatization for **2f.** The bottom pane shows an enlarged view of the **2f** peak and dehydration product peak. **Conditions:** 50 mM **1f**, 250 mM L-asp, PLP (50x to catalyst), 7G11 (0.1 mol% cat, 1000 Max TON), 5% MeOH, 100 mM potassium phosphate buffer, pH 7.0, 100 mM NaCl, 37 °C, 4 h.

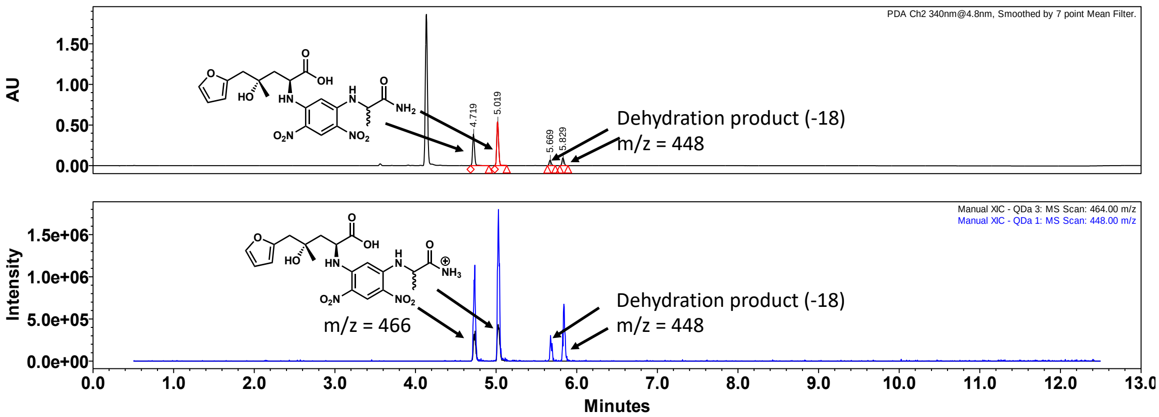

Figure S29. UPLC-MS trace of racemic Marfey's derivatization for **2f**.

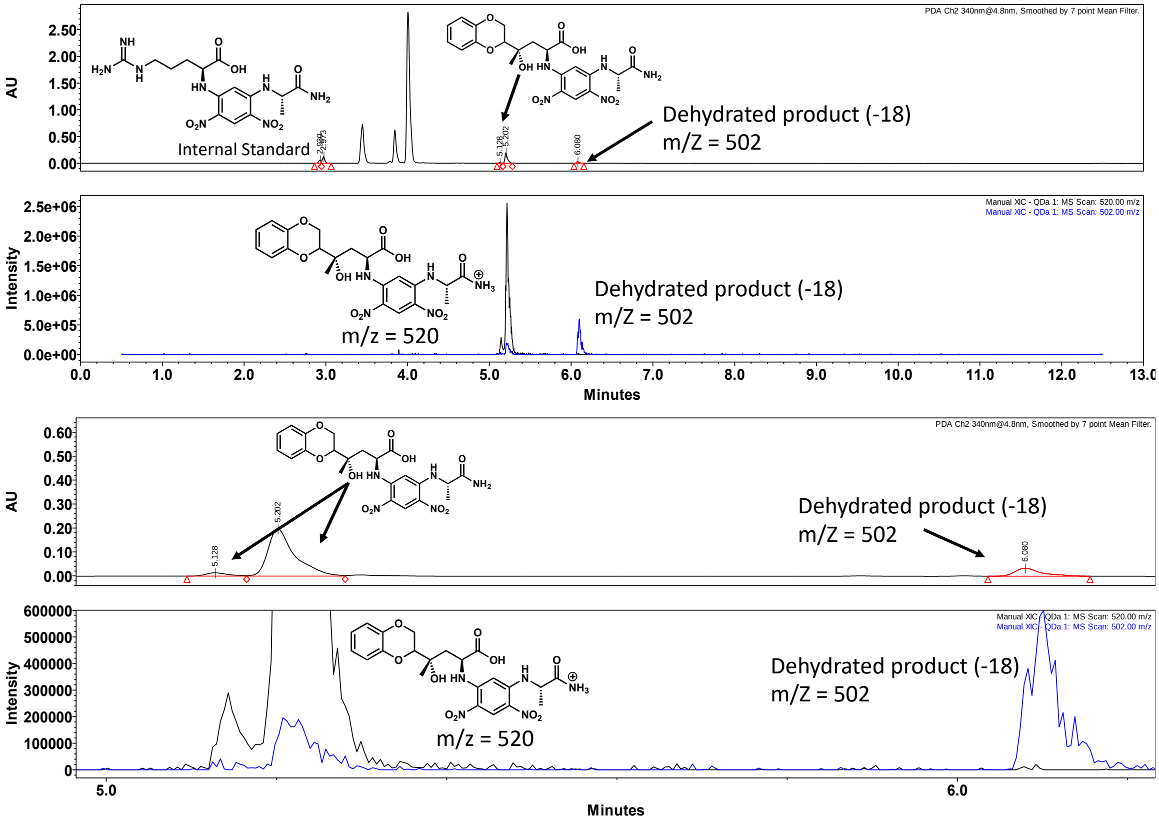

Figure S30. UPLC-MS trace of Marfey's derivatization for **2i.** The bottom pane shows an enlarged view of the **2i** derived product peaks indicating the presence of two diastereomers with the depicted diastereomer as the major isomer. **Conditions:** 50 mM **1i**, 250 mM L-asp, PLP (50x to catalyst), 7G11 (0.1 mol% cat, 1000 Max TON), 5% MeOH, 100 mM potassium phosphate buffer, pH 7.0, 100 mM NaCl, 37 °C, 4 h.

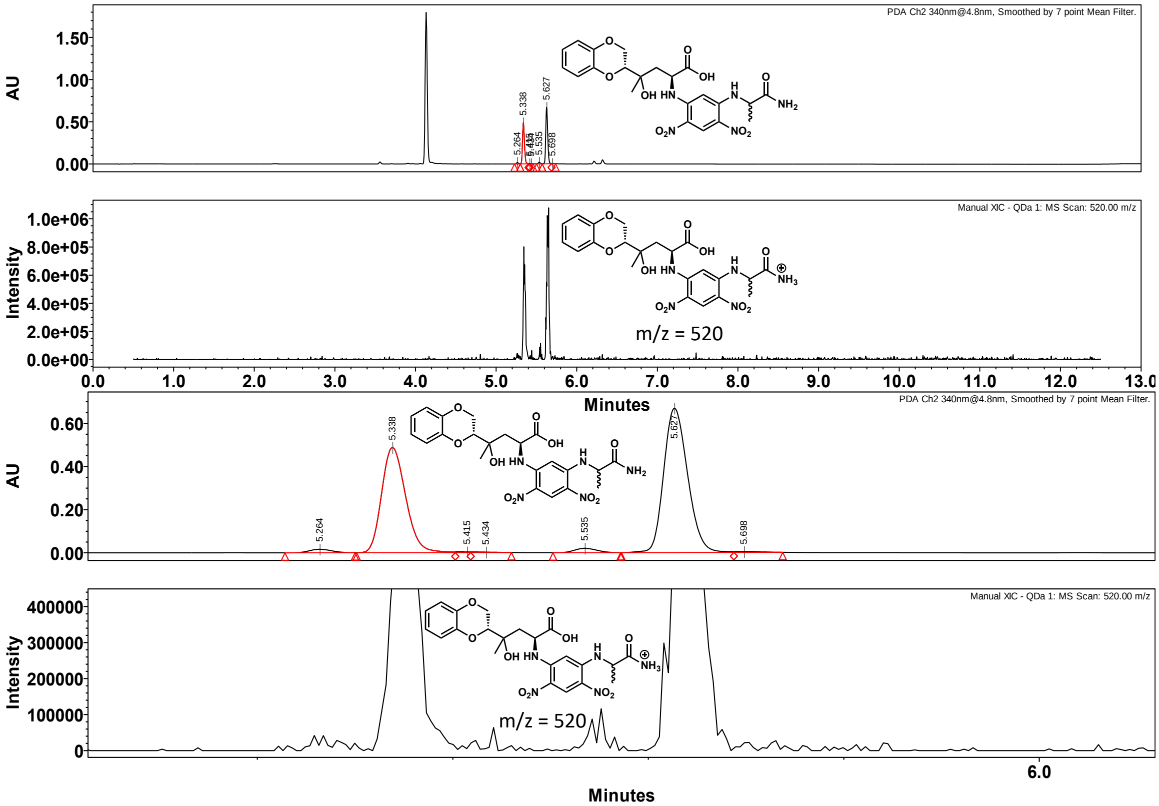

Figure S31. UPLC-MS trace of racemic Marfey's derivatization for **2i.** The bottom pane shows an enlarged view of the **2i** peaks to display the presence of the minor diastereomer.

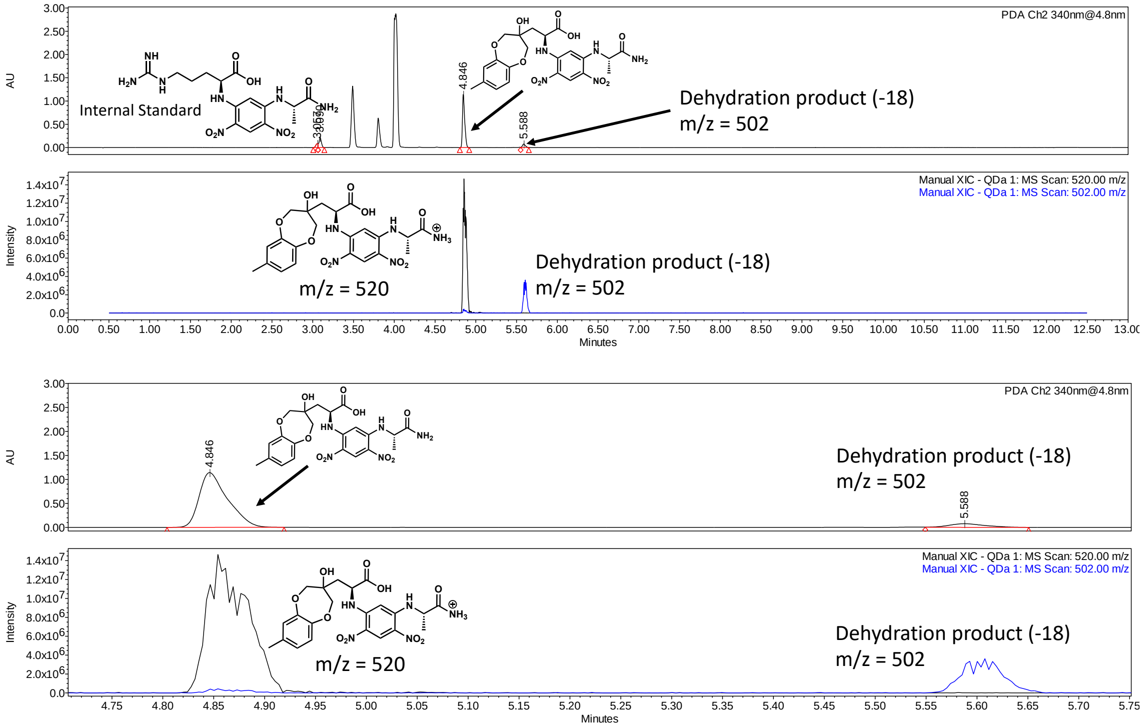

Figure S32. UPLC-MS trace of Marfey's derivatization for **2j**. The bottom pane shows an enlarged view of the **2j** product derived peaks. **Conditions:** 50 mM **1j**, 250 mM L-asp, PLP (10x to catalyst), 7G11 (0.1 mol% cat, 1000 Max TON), 5% MeOH, 100 mM potassium phosphate buffer, pH 7.0, 100 mM NaCl, 37 °C, 4 h.

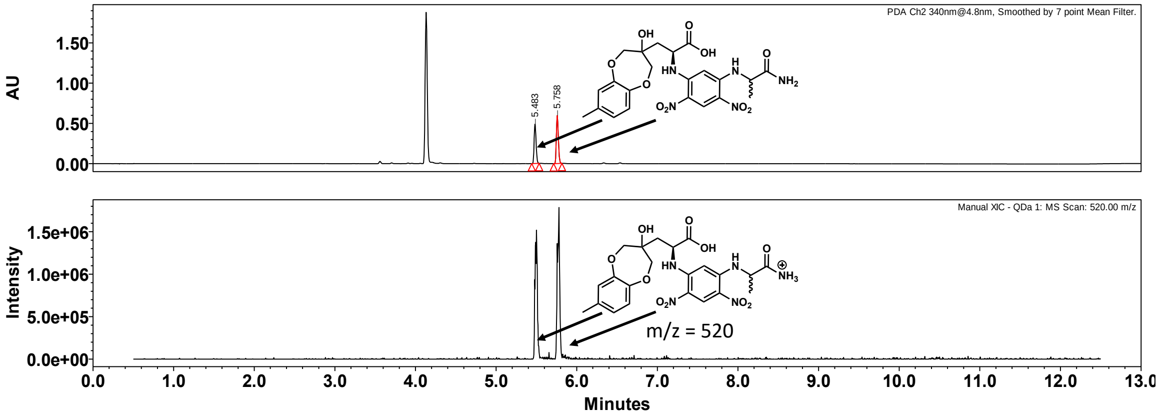

Figure S33. UPLC-MS trace of racemic Marfey's derivatization for **2j.**

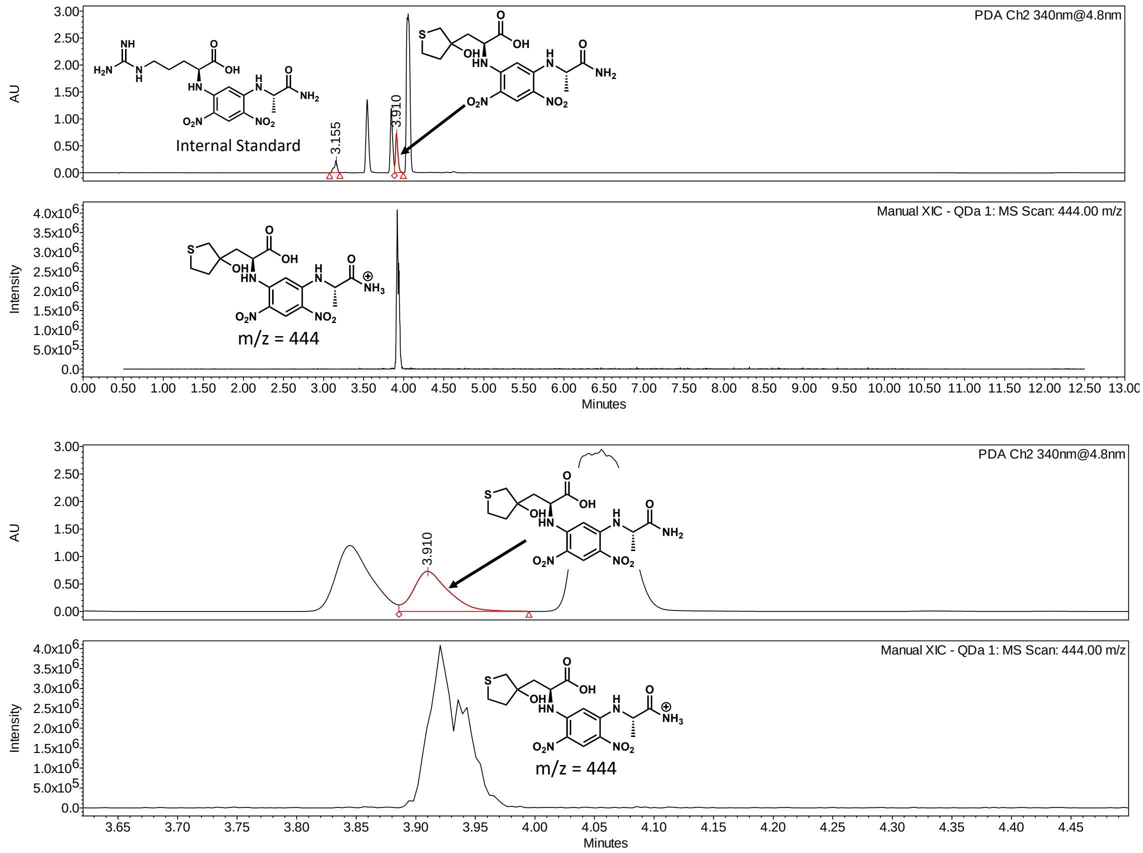

Figure S34. UPLC-MS trace of Marfey's derivatization for **2k.** The bottom pane shows an enlarged view of the **2k** product peak. **Conditions:** 50 mM **1k**, 250 mM L-asp, PLP (50x to catalyst), 7G11 (0.1 mol% cat, 1000 Max TON), 5% MeOH, 100 mM potassium phosphate buffer, pH 7.0, 100 mM NaCl, 37 °C, 4 h.

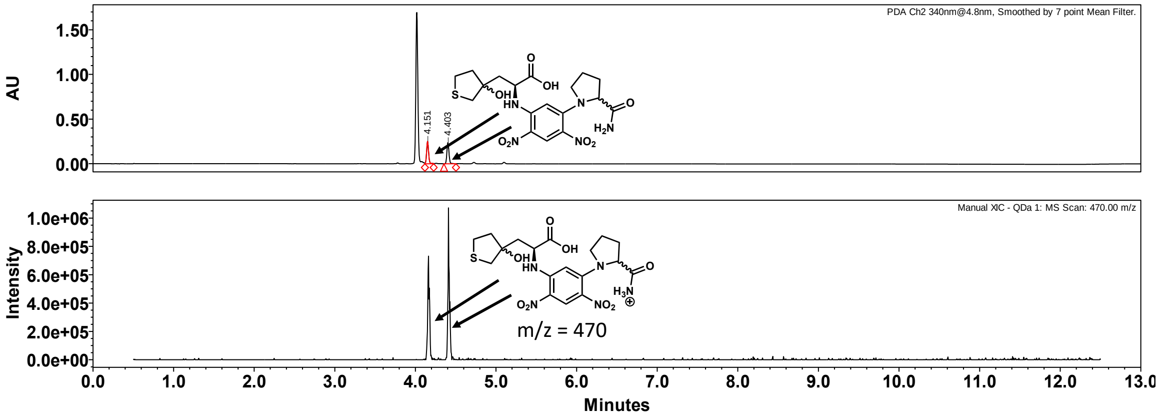

Figure S35. UPLC-MS trace of racemic Marfey's derivatization for **2k.**

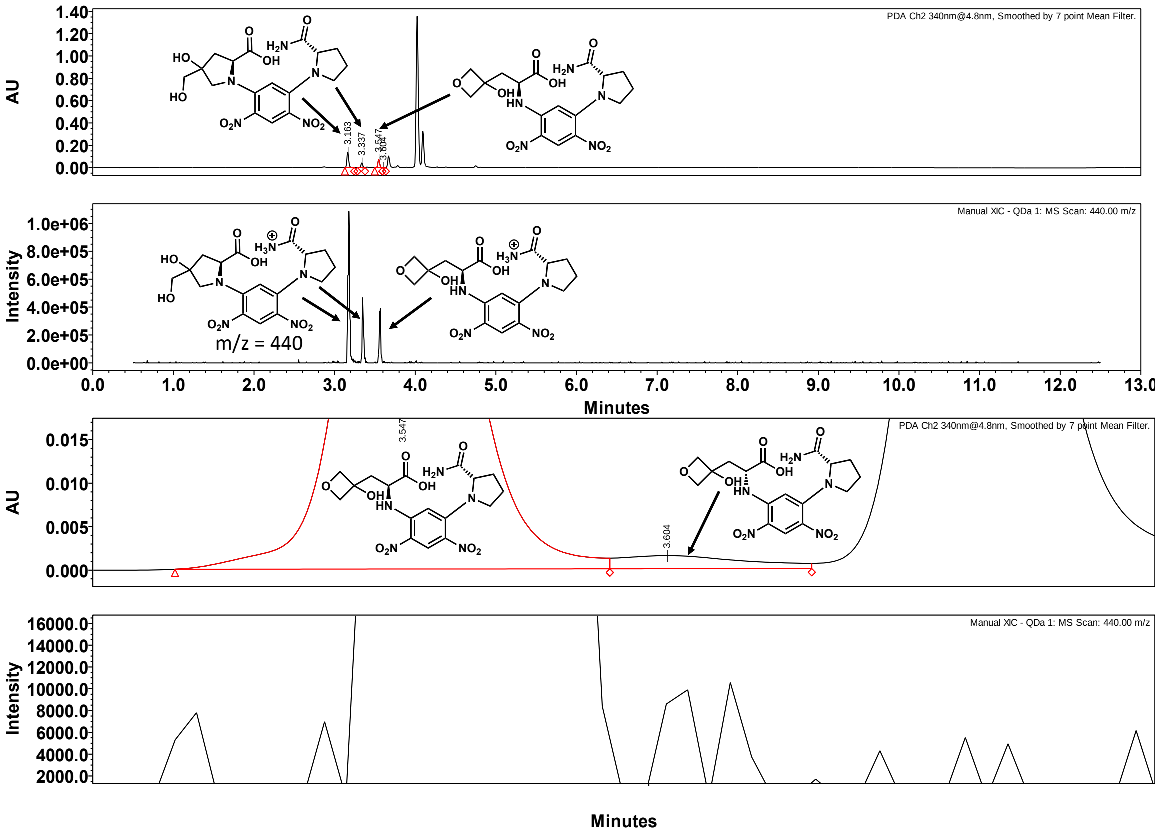

Figure S36. UPLC-MS trace of racemic Marfey's derivatization for **2l.** The bottom pane shows an enlarged view of the **2l** product peak to display the small amount of the *R* enantiomer observed. **Note:** Under Marfey's conditions, **2l** is prone to an intramolecular ring opening rearrangement giving rise to two diastereomeric proline derivatives that correspond to the first two peaks.

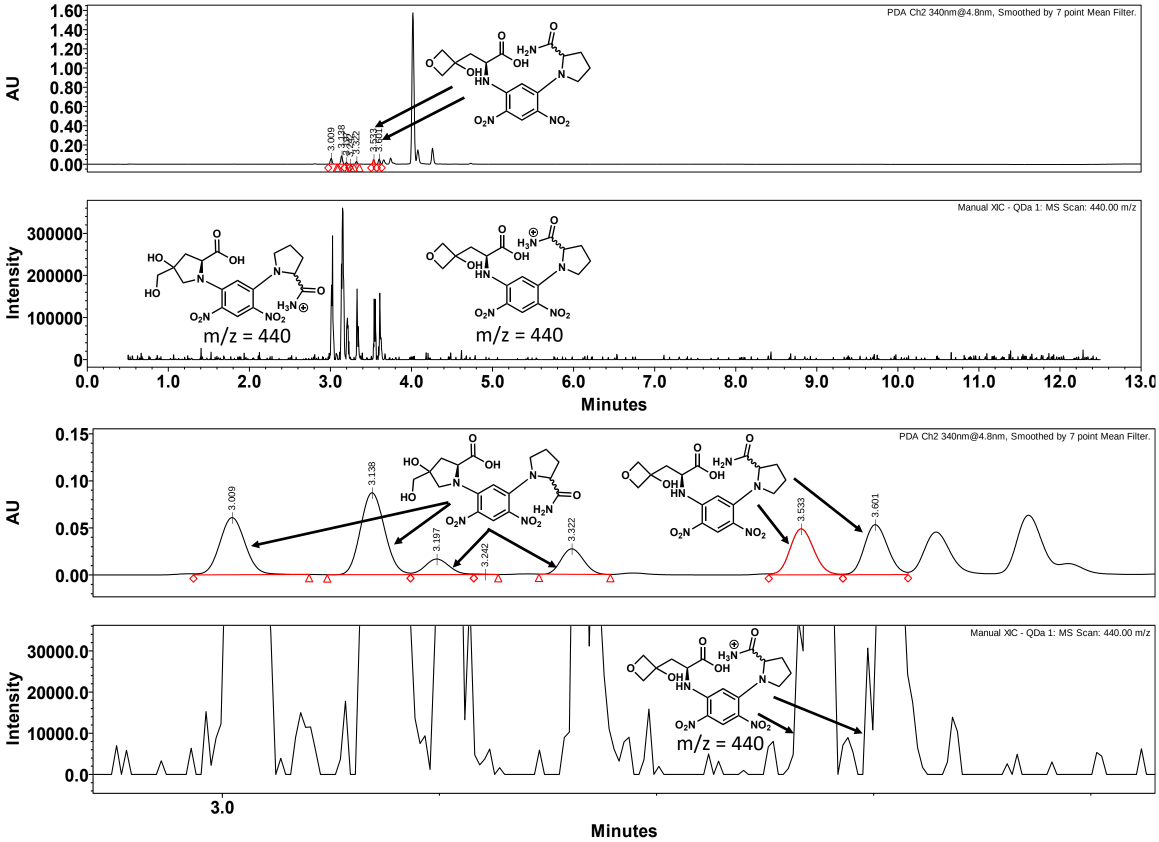

Figure S37. UPLC-MS trace of racemic Marfey's derivatization for **2l**. The bottom pane shows an enlarged view of the **2l** product peaks. **Note:** Under Marfey's conditions, the product is prone to an intramolecular ring opening rearrangement giving rise to two diastereomeric proline derivatives that correspond to the first four peaks.

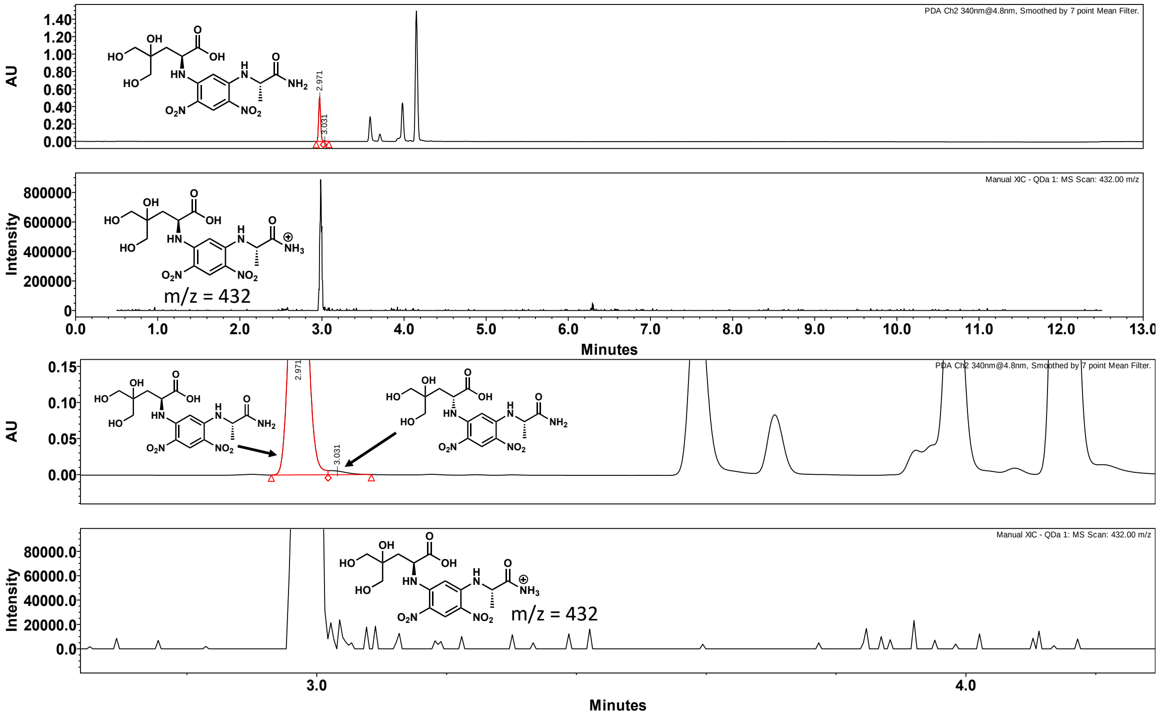

Figure S38. UPLC-MS trace of Marfey's derivatization for **2m**. The bottom pane shows an enlarged view of the **2m** product peak to display the small amount of the *R* enantiomer observed. Conditions: 50 mM **1m**, 250 mM L-asp, PLP (50x to catalyst), 7G11 (0.1 mol% cat, 1000 Max TON), 5% MeOH, 100 mM potassium phosphate buffer, pH 7.0, 100 mM NaCl, 37 °C, 4 h.

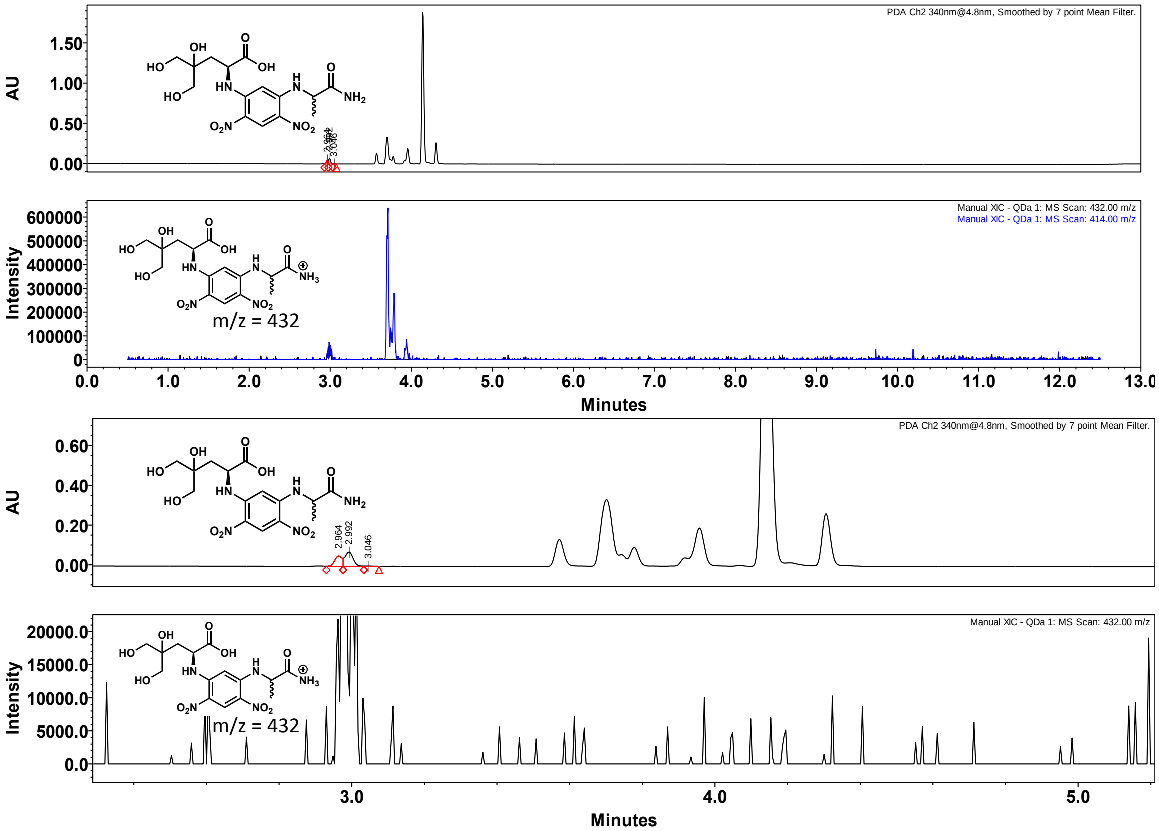

Figure S39. UPLC-MS trace of racemic Marfey's derivatization for **2m**. The bottom pane shows an enlarged view of the **2m** product peaks.

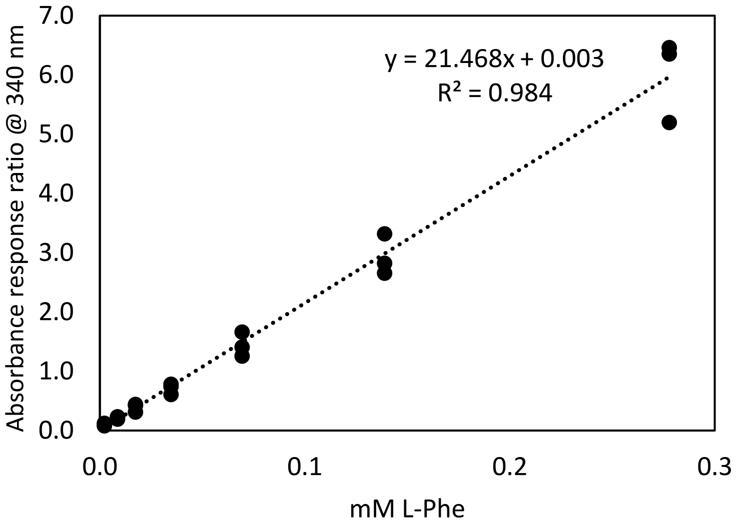

Figure S40. Marfey's calibration curve by absorbance at 340 nm. Standard curve used for quantification of TTN in the lineage analysis. Marfey's derivatization is performed using L-phe as a product mimic. The absorbance response ratio of labeled L-phe to labeled internal standard (L-arg) was measured at 340 nm. Points on the curve represent the individual measurements of technical triplicate measurements.

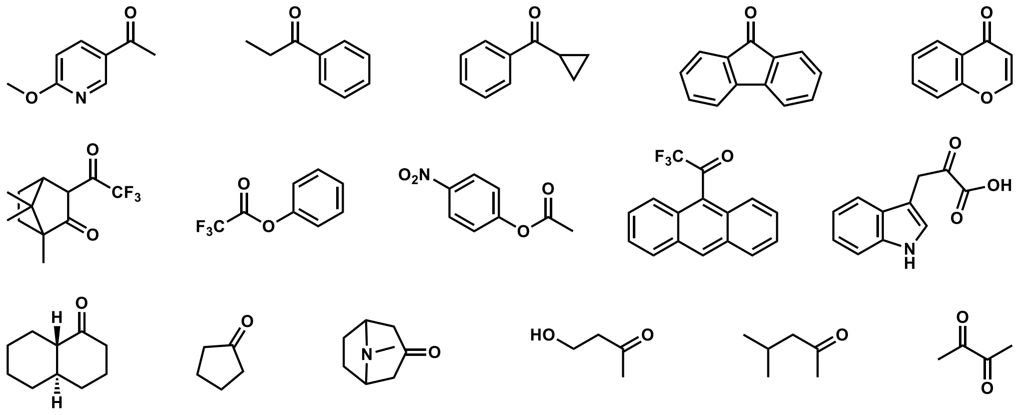

Figure S41. Substrates that are inactive with 7G11 and 7B05.

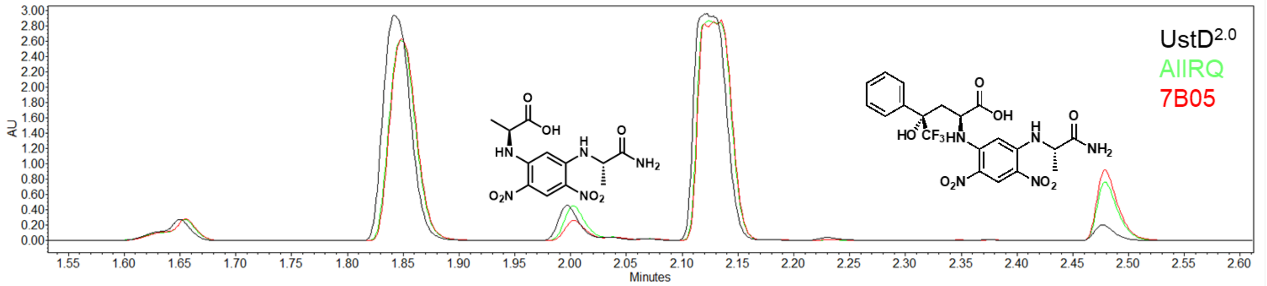

Figure S42. Overlay of UstD$^{2.0}$ (black), AIIRQ (green), and 7B05 (red) lineage reactions for product **2d**.

Table S1. Distal recombination library amino acid residues possible at each site.

**site**	**F75**	**D86**	**I141**	**V330**	**S407**
mutation	F<br>A<br>V	D<br>I<br>V<br>N	I<br>V<br>M	Q<br>A<br>R<br>E<br>P<br>L<br>G<br>V	S<br>T<br>Q<br>A<br>E
size	3	4	3	8	5

Table S2. Active site recombination library amino acid residues possible at each site.

**site**	**M299**	**T388**	**T391**	**L392**	**M393**
mutation	M<br>L<br>V	T<br>V<br>I<br>A	T<br>F<br>S<br>L<br>P<br>I	L<br>V<br>A<br>S	M<br>W<br>F<br>L<br>R<br>C<br>I<br>S
size	3	4	6	4	8

Table S3. Complied directed evolution results from all rounds of evolution.

Round	Description	Clones Screened	Variants with altered promiscuity and/or activity in screening	Best Variant(s)
1	Random mutagenesis of the entire gene	880	G373R, D86V, F75S, Y96C+G101R, P82S+V330A, P83R, Y277C+K342E+S407N, P80L, H263R, Y418H	UstD$^{2.0}$ All variants were generally deactivated but changed promiscuity. No mutations were fixed this round.
2	Site saturation of 'hotspots' with UstD$^{2.0}$: F75, P80, P82, G373	352	F75: A, C, H, I, K, L, M, N, Q, R, S, T, W, Y<br>P80: G, R<br>P82: G, Q<br>G373: E, R	P82Q<br>G373E<br>F75A
3	Double mutant P82Q+G373E	1	P82Q+G373E	QE = P82Q+G373E
4	Site saturation of 'hotspots' with QE as parent: P83, D86, Y96, G101, I141, Y277, V330, K342, S407, Y418,	880	P83: G, V, T, Y<br>D86: I, V<br>G101: F, Q, A+ΔH445<br>I141: V, M,<br>Y277: H, F+W399C<br>V330: Q, A, R, C<br>K342: none<br>S407: Q, T, A, E<br>Y418: none<br>* silent mutations were fixed through primer design I141 (ATC→ ATT)	None. Used mutational information from D86, I141, V330, and S407 in subsequent library
5	Recombination at sites F75, D86, I141, V330, S407	704	F75A+D86I+I141V+S407E<br>F75A+D86I+V330R+S407Q<br>F75V+D86N+V330V<br>F75A+D86V+I141V+S407Q<br>F75A+D86V+I141M+S407E<br>F75V+D86V+S407Q<br>F75A+D86V+V330L+S407E<br>F75A+D86I+V330A+S407Q<br>F75V+D86V+S407E<br>F75A+D86I+V330A+S407Q<br>K2E+F75V+D86I+V330V<br>F75A+D86V+V330Q+S407E<br><br>*synonymous mutations not included	AIIRQ= QE+ F75A+ D86I+ I141I(ATC→ATT)+ V330R+ S407Q
6	Recombination at active site residues M299, T388, T391, L392, M393	968	M299L+T388V+T391F<br>M299V+T388V<br>M299V+T388V+T391S+L440P<br>M299V+T388I+T391F+M393C<br>M299L+T388I+T391S+M393C<br>M299V+T388I<br>T388V+T391F+M393S<br>T388I+T391S<br>T391S+M393W<br>C201C+M299V+T391S+M393F<br>M299L+T388A+T391F<br>M299V+M393W<br>M299V+T388V+M393W<br>M299V+T388V+M393W<br>T391S+M393F<br>M299V+T388A+T391F+M393W	7G11 = AIIRQ+ M299V+ T391S+ M393W<br><br>7B05 = AIIRQ+ T391S+ M393F

			M299V+T388I<br>M299V+T391S+M393W<br>M299V+T388V+T391F+M393F<br>M299L+T391S+M393W<br>M299V+T388I+T391S+M393F<br>M299V+T388I+T391F<br>M299V+T391S+L392A+M393W<br>T388I<br>V63I<br>M299V+T391F+M393W<br>M299V+T388I+M393F<br>M299V+T388I+M393L<br>M299V+T388A+T391F+M393W<br>M299L<br>T391F+M393W<br>T391F+L392I+M393W<br>T388A+T391F	
7	Site saturation of various residues both in and out of active site: Y257, H263, F285, P300, S389, H283. Each library was generated with 7G11 and 7B05 as the parent enzyme.	440	none	No variants surpassed 7G11 or 7B05 activity, so no further engineering was pursued.

Table S4. Lineage reaction conditions by electrophile.

Electrophile	Molar equivalents PLP	Max TON	mol% catalyst
Benzaldehyde **(1g)**	10	30000	0.003
1,1,1-trifluoro-3-phenyl-2-propanone **(1d)**	10	5000	0.02
2-furylacetone **(1f)**	50	2500	0.04
(4-fluorophenyl)acetone **(1c)**	50	1000	0.1

Table S5. Turnover numbers for ketone lineage analysis.

**Variant**	**UstD$^{2.0}$**	**SA**	**Q**	**QE**	**AIIRQ**	**7G11**	**7B05**
Product	Avg TTN	Avg TTN	Avg TTN	Avg TTN	Avg TTN	Avg TTN	Avg TTN
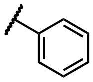 R = H	9700 ± 500	3900 ± 200	4300 ± 100	4300 ± 300	3800 ± 200	1800 ± 100	4900 ± 200
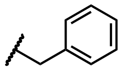 R = CF_3	480 ± 50	1110 ± 90	1000 ± 100	1070 ± 40	1900 ± 100	1560 ± 50	2120 ± 40
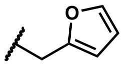 R = CH_3	126 ± 6	160 ± 10	160 ± 10	200 ± 20	370 ± 30	740 ± 10	580 ± 10
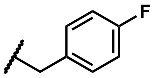 R = CH_3	trace	~ 10	~ 10	17 ± 1	18 ± 1	67 ± 6	29 ± 2

Table S6. Crystal data and structure refinement for **2i.**

Empirical formula	$C_{13}H_{17}NO_5 \bullet H_2O$
Formula weight	285.29
Temperature/K	100.00
Crystal system	triclinic
Space group	*P*1
a/Å	5.7856(6)
b/Å	6.2026(6)
c/Å	20.699(2)
α/°	94.081(6)
β/°	90.513(6)
γ/°	115.481(6)
Volume/Å^3	668.19(13)
Z	2
ρ_{calc}g/cm^3	1.418
μ/mm^{-1}	0.951
F(000)	304.0
Crystal size/mm^3	0.055 × 0.041 × 0.011
Radiation	Cu Kα (λ = 1.54178)
2Θ range for data collection/°	8.574 to 144.67
Index ranges	-7 ≤ h ≤ 7, -7 ≤ k ≤ 7, -25 ≤ l ≤ 25
Reflections collected	22465
Independent reflections	5087 [R_{int} = 0.0300, R_{sigma} = 0.0235]
Data/restraints/parameters	5087/56/399
Goodness-of-fit on F^2	1.060
Final R indexes [I>=2σ (I)]	R_1 = 0.0260, wR_2 = 0.0651
Final R indexes [all data]	R_1 = 0.0266, wR_2 = 0.0656
Largest diff. peak/hole / e Å^{-3}	0.20/-0.21
Flack parameter	-0.02(6)

Table S7. Crystal data and structure refinement for **2k.**

Empirical formula	$C_{14}H_{26}N_2O_6S_2$
Formula weight	382.49
Temperature/K	100.00
Crystal system	triclinic
Space group	P1
a/Å	5.4808(7)
b/Å	5.6991(7)
c/Å	13.8474(14)
α/°	80.745(8)
β/°	87.828(6)
γ/°	79.666(9)
Volume/Å^3	419.96(9)
Z	1
ρ_{calc}g/cm^3	1.512
μ/mm^{-1}	3.187
F(000)	204.0
Crystal size/mm^3	0.08 × 0.02 × 0.02
Radiation	CuKα (λ = 1.54178)
2Θ range for data collection/°	6.468 to 160.754
Index ranges	-6 ≤ h ≤ 6, -7 ≤ k ≤ 7, -17 ≤ l ≤ 17
Reflections collected	15945
Independent reflections	3413 [R_{int} = 0.0636, R_{sigma} = 0.0514]
Data/restraints/parameters	3413/20/241
Goodness-of-fit on F^2	1.090
Final R indexes [I>=2σ (I)]	R_1 = 0.0423, wR_2 = 0.1111
Final R indexes [all data]	R_1 = 0.0436, wR_2 = 0.1121
Largest diff. peak/hole / e Å^{-3}	0.32/-0.38
Flack parameter	0.014(16)

SI References

(1) Kille, S.; Acevedo-Rocha, C. G.; Parra, L. P.; Zhang, Z.-G.; Opperman, D. J.; Reetz, M. T.; Acevedo, J. P. Reducing Codon Redundancy and Screening Effort of Combinatorial Protein Libraries Created by Saturation Mutagenesis. *ACS Synth Biol* **2013**, *2* (2), 83–92. https://doi.org/10.1021/sb300037w.

(2) Gibson, D. G.; Young, L.; Chuang, R.-Y.; Venter, J. C.; Hutchison, C. A.; Smith, H. O. Enzymatic Assembly of DNA Molecules up to Several Hundred Kilobases. *Nat Methods* **2009**, *6* (5), 343–345. https://doi.org/10.1038/nmeth.1318.

(3) Nath, A.; Atkins, W. M. A Quantitative Index of Substrate Promiscuity. *Biochemistry* **2008**, *47* (1), 157–166. https://doi.org/10.1021/BI701448P/SUPPL_FILE/BI701448P-FILE007.PDF.

(4) Weeks, A. M.; Wells, J. A. Engineering Peptide Ligase Specificity by Proteomic Identification of Ligation Sites. *Nature Chemical Biology 2017 14:1* **2017**, *14* (1), 50–57. https://doi.org/10.1038/nchembio.2521.

(5) Mcdonald, A. D.; Higgins, P. M.; Buller, A. R. Substrate Multiplexed Protein Engineering Facilitates Promiscuous Biocatalytic Synthesis. *Nat. Commun.* **2022**, *13*, 5242. https://doi.org/10.1038/s41467-022-32789-w.
